# Transcriptional Landscape and Splicing Efficiency in *Arabidopsis* Mitochondria

**DOI:** 10.3390/cells10082054

**Published:** 2021-08-11

**Authors:** Laura E. Garcia, M. Virginia Sanchez-Puerta

**Affiliations:** 1 IBAM, Facultad de Ciencias Agrarias, Universidad Nacional de Cuyo, Conicet, Almirante Brown 500, Chacras de Coria, Mendoza M5528AHB, Argentina; mvsanchezpuerta@fca.uncu.edu.ar; 2 Facultad de Ciencias Exactas y Naturales, Universidad Nacional de Cuyo, Padre Jorge Contreras 1300, Chacras de Coria, Mendoza M5502JMA, Argentina

**Keywords:** co-transcription, mitochondrial genomes, splicing efficiency, promoter, RNAseq

## Abstract

Plant mitochondrial transcription is initiated from multiple promoters without an apparent motif, which precludes their identification in other species based on sequence comparisons. Even though coding regions take up only a small fraction of plant mitochondrial genomes, deep RNAseq studies uncovered that these genomes are fully or nearly fully transcribed with significantly different RNA read depth across the genome. Transcriptomic analysis can be a powerful tool to understand the transcription process in diverse angiosperms, including the identification of potential promoters and co-transcribed genes or to study the efficiency of intron splicing. In this work, we analyzed the transcriptional landscape of the *Arabidopsis* mitochondrial genome (mtDNA) based on large-scale RNA sequencing data to evaluate the use of RNAseq to study those aspects of the transcription process. We found that about 98% of the *Arabidopsis* mtDNA is transcribed with highly different RNA read depth, which was elevated in known genes. The location of a sharp increase in RNA read depth upstream of genes matched the experimentally identified promoters. The continuously high RNA read depth across two adjacent genes agreed with the known co-transcribed units in *Arabidopsis* mitochondria. Most intron-containing genes showed a high splicing efficiency with no differences between cis and trans-spliced introns or between genes with distinct splicing mechanisms. Deep RNAseq analyses of diverse plant species will be valuable to recognize general and lineage-specific characteristics related to the mitochondrial transcription process.

## 1. Introduction

Mitochondria are essential organelles of eukaryotic organisms that originated from α-proteobacteria and carry their own genetic material and gene expression system. Since the endosymbiotic event, the mitochondrial genome has undergone severe reduction in gene content through the functional transfer of genes to the nucleus and the definitive loss of many of them [1]. Plant mitochondrial genomes exhibit highly variable genome sizes, but even when extraordinarily large (up to 11.7 Mb [2]), the gene content is conserved (usually less than 60 genes). Thus, most of the genome consists of non-coding regions [3,4,5,6,7,8,9,10] with a variable proportion of repeats [11] that may recombine resulting in rearranged genomes [12,13]. Recombination events across repeats may place non-coding regions near regulatory elements leading to the transcription of non-functional DNA [14]. The transcriptional control of plant mitochondrial genomes is relaxed, frequently with more than one promoter per gene and these promoters exhibiting non-canonical motifs [15]. *Arabidopsis* has been the focus of extensive experimental studies to understand the mitochondrial gene expression process, including intron splicing mechanisms [16,17,18], transcription initiation and termination [14,19] and identification of co-transcribed units [20,21,22]. However, a thorough analysis of the *Arabidopsis* mitochondrial transcriptional landscape is still lacking.

Over the past decade, several studies on plant mitochondrial transcriptomes have been published focusing mainly on the identification of RNA editing sites, the expression of unidentified ORFs or the extent of transcription across the genome [2,23,24,25,26]. Even though plant mitochondrial genomes consist predominantly of intergenic DNA, deep RNAseq experiments revealed that non-coding regions are fully or almost-fully transcribed [27], but with transcript levels so low over most of the genome as to be considered functionally insignificant [28,29,30,31,32]. Occasionally, non-coding regions show high RNA read depth suggesting a possible role as long non-coding RNAs. These regions are generally not conserved and have not been further characterized [33,34,35].

Furthermore, RNAseq could be valuable to investigate other aspects of mitochondrial transcription, such as identifying transcription boundaries and potential promoters, inferring which genes are co-transcribed or analyzing the efficiency of the splicing process. For example, co-transcription has been presumed when high RNA read depth was observed across two closely located genes in plant mitochondrial genomes [23,29]. However, only a few studies have unequivocally determined co-transcribed units [20,21,22,36], and such evidence is required to validate the inferences based on RNAseq data. In addition, intron splicing efficiency could be easily assessed by comparing the levels of spliced and unspliced RNA species. The splicing process of intron-containing genes in plant mitochondria has been analyzed for the most part in mutants in order to identify the nuclear factors involved [25,37,38,39]. However, the efficiency of the splicing activity has only rarely been studied in wild type plants [26,33] with no comparative analyses among introns, either group I or II, or cis- and trans-spliced introns that co-exist in plant mitochondrial genomes.

In this work, we analyzed the *Arabidopsis* mitochondrial transcriptional landscape based on large-scale RNAseq data sets to shed light on: (i) the transcription extent of the mitochondrial genome; (ii) the usefulness of the RNAseq profile to infer transcription start sites, 3′ ends and the co-transcription of gene clusters; and (iii) the splicing efficiency of the 23 cis- or trans-spliced introns in the *Arabidopsis* mitochondrial genome.

## 2. Results and Discussion

### 2.1. The Mitochondrial Genome of Arabidopsis Is Entirely Transcribed with Highly Variable Levels of Read Depth

Even though *Arabidopsis* has been the focus of numerous studies centered on plant mitochondria [40,41,42,43], a genome-wide analysis of the transcriptional landscape of the mitochondrial genome (mtDNA) has not been reported. In the past decade high-throughput RNAseq analyses provided new insights into the complexity of RNA metabolism in plant mitochondria and indicated that the regulation of mitochondrial RNA processing plays a critical role in plant organellar gene expression [14,20]. Detailed experimental assays were instrumental to identify transcription initiation sites for each mitochondrial gene and to characterize post-transcriptional processes in *Arabidopsis* mitochondria [14,20,36,42,44]. To gain insight into different aspects of the transcription profile in the *Arabidopsis* mtDNA, we analyzed publicly available stranded RNAseq data sets from three biological replicates (BioProject PRJEB15579). The data sets consist of total RNA depleted of ribosomal RNA, extracted from a 14-day seedling of wild type *Arabidopsis thaliana* ecotype Columbia. We analyzed each of the three data sets individually and combined, totaling 617,847,968 paired-end reads. This volume of data is comparable with previous, large-scale RNAseq studies on angiosperm mitochondria [23,33,45]. The pooled RNAseq reads were aligned strand specifically to the corrected sequence of the *A.* *thaliana* ecotype Columbia mtDNA, NCBI accession number BK010421 [46]. The *Arabidopsis* mtDNA is 367,808 bp in length and includes 33 protein, three rRNA and 22 tRNA coding genes [46]. The coding regions represent around 10.4% of the genome (5% when considering both strands), whereas ~90% are intergenic sequences or unidentified ORFs.

About 7 million reads map to either strand of the mtDNA, masking plastid derived regions to avoid mismapping of plastid RNA (Figure S1). Considering both strands, the mean read depth is 861 and the maximum is 194,844. Excluding known genes, the mean read depth of the intergenic regions is 335. We plotted the frequency of the read depth across the two strands of the mtDNA based on 100-bp windows at selected read depth intervals (Figure S2). About 10% of the mtDNA shows a read depth above 1000, 30% above 100 and 43% above 50 reads (Figure S2). A total of 98%, 95.6% and 88.55% of the mtDNA (considering both strands) is covered by at least one, two and five reads, respectively (Figure S2). The regions with poor or zero read depth are dispersed across the genome ranging from 1 to 6050 bp in length (Figure S1). The alignment of a large amount of sequence data from mitochondrial RNA shows that almost the whole mtDNA of *Arabidopsis*, although at low levels, is transcribed. This genome-wide transcription has also been reported in other species [27,31,33] in which deep RNAseq experiments were analyzed. The amount of RNAseq data may be limiting the extent of the genome with mapped RNA reads, as observed in other species in which non-coding regions were shown to be transcribed but not the full mtDNA [29,31]. We speculate that an even larger RNAseq data set would likely reveal that both strands of the *Arabidopsis* mtDNA are entirely transcribed, with low read depth regions representing the major fraction. This genome-wide transcription can be explained by the relaxed nature of the plant mitochondrial transcription process, involving multiple and variable start of transcription sites, frequent genome recombination across the multiple repeats that can relocate promoters to non-coding regions and post-transcriptional processing of the transcripts [14,44,47,48].

Given the genome-wide transcription in plant mitochondria, inferring biologically significant transcribed regions is fundamental to the identification of functional ORFs. A recent study of mitochondrial expression based on RNAseq proposed that a significant read depth, well above the background RNA read depth of the mtDNA, along with efficient RNA editing, and accurate intron splicing, constitutes strong evidence of functionality of mitochondrial genes [33]. The pervasive transcription of non-coding regions calls into question the evidence of mitochondrial gene expression based on transcripts recovered by non-quantitative techniques, given that transcripts from any region of the plant mtDNA could be observed. For example, based on total RNA extracted from multiple organs, transcription of the nuclear-derived mitochondrial-encoded orf164 was detected by RT-PCR at lower abundance than other mitochondrial genes [49]. The present transcriptional landscape analysis shows only a basal RNA read depth for orf164 (Figure S1), even lower than the average for the genome. Furthermore, expression of this ORF was not detected in translation analyses [50]. These results indicate the orf164 is not significantly transcribed and it is likely not functional, at least in the phenological stage studied here and by Planchard et al., 2018 [50].

### 2.2. Protein-Coding Genes Show Elevated RNA Read Depth

Despite the relaxed transcription process in plant mitochondria, the expression of functional genes is greatly efficient. With very few exceptions, only known coding regions show RNA read depths that are several times greater than those of non-coding regions [29,33,34]. In this study, we found that the RNA read depth of the majority of the protein-coding genes is five-fold higher than the average read depth for the whole genome, and even greater if the average read depth for non-coding regions is considered (Table S1). These results agree with a study of polysomal RNA in which only transcripts from all protein-coding mitochondrial genes of *Arabidopsis* were associated to ribosomes and none from the intergenic regions [50].

**Figure 1:**
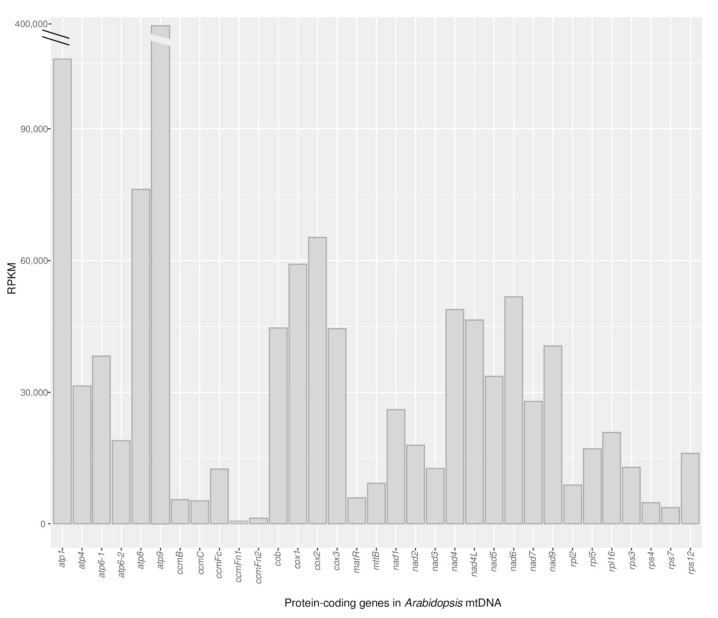
RPKM of the coding regions of protein-coding genes based on the combined RNAseq data sets of the three biological samples. The interrupted RPKM for *atp9* is 381,229.

The *Arabidopsis* mtDNA contains 33 protein-coding genes, including the two non-identical copies of *atp6* [51]. To assess the RNA read depth of each protein-coding gene, the three RNAseq data sets were analyzed individually and combined (Table S1). Here, we estimated the number of RNA molecules present in the cell in a precise moment (steady-state) without distinguishing between those that are being transcribed and those that belong to the population of RNA molecules that are being processed or degraded. The RNA read depth of each mitochondrial gene was normalized by the transcript length and by the total number of reads mapped to all genes in the *Arabidopsis* mtDNA [52]. A frequent unit to express the normalized RNA read depth per gene is RPKM (Reads Per Kilobase of transcript, per Million mapped reads). The RPKM values of the known protein-coding genes differ widely among (Figure 1) and within (Figure S3) genes. This variability in transcription levels has been reported before for *Arabidopsis* using other approaches as run-on transcription and Northern blots [20] and for other plant mitochondrial genomes based on RNAseq data [23,31,33,34]. The RNA read depth of each of the 33 protein-coding genes is consistent across the three samples (Table S1). In agreement with previous studies in *Arabidopsis* [20] and other plants [35], *atp9* shows the highest read depth (RPKM = 361,785–369,149), while *ccmFn1* (RPKM = 529–638) followed by *ccmFn2* (RPKM = 998–1421), *rps7* (RPKM = 3911–3580) and *rps4* (RPKM = 4576–4635) show the lowest read depth across the three samples. The two copies of *atp6* show different RNA read depth, with that of *atp6–1* being consistently greater than that of *atp6-2* in the three data sets. This observation agrees with the increased association of *atp6-1 m*RNA to ribosomes reported by Planchard et al., 2018 [50]. In addition, and in line with previous studies of *Arabidopsis* and other angiosperm mitochondria, genes coding for subunits of respiratory complexes showed much higher read depth compared to other mitochondrial protein-coding genes (Table S1, Figure 1).

Given that intron-containing genes are transcribed and further processed to remove introns, the RNA reads derive from unspliced and spliced versions of the transcripts. To evaluate the abundance of RNA reads at both stages, unspliced and spliced reference sequences of these genes were used for RNA alignment. Thus, RNA reads derived entirely from exons map to both references, while those that expand exon-intron or exon-exon junctions map only to the unspliced or spliced versions, respectively. These data are instrumental to calculate the splicing efficiency of each gene (see below). In general, there are more RNA reads derived from spliced than from unspliced transcripts (Figure S3).

Notably, there are differences in the read depth among exons in intron-containing genes of the *Arabidopsis* mtDNA (Figure S3); e.g., exon 4 of *nad5* (*nad5e4*) shows twice the read depth of exon 2. This variability has also been observed in other species [33,34] and reinforces the fact that the expression of mitochondrial genes in plants is regulated primarily at the post-transcriptional level [53]. In some cases, observations in other Brassicaceae [34] contrast with those in *Arabidopsis*. For example, the read depth of exon 5 of the gene *nad1* (*nad1e5*) is seventh-fold higher than that of exon 1 in *Arabidopsis*, while the opposite pattern was observed in *Brassica oleracea* [34].

By mapping RNAseq reads to the coding regions of each gene, we observed drops in the read depth near the end of *ccmC*, *mtttB* and *nad6* (Figure S3), as reported previously for *Arabidopsis* [50]. A drop in the read depth of the genes *ccmC* and *nad6* has also been reported for other angiosperms, such as *Silene noctiflora* [30], *Allium cepa* [24], *Solanum tuberosum* [23] and *Lophophytum mirabile* [33]. These drops are probably caused by 3′ endonucleolytic processing events signaled by tRNA-like elements embedded in the 3′ ends of these genes [36,54]. In addition, a late increase in read depth is observed inside the coding region of *rps4* (Figure S3), which agrees with the location of the promoter identified experimentally [36].

### 2.3. The RNAseq Transcriptional Landscape Is Useful to Infer the Region of Transcription Start

Given that promoters are not conserved at the sequence level and exhibit non-canonical motifs, and that multiple transcription start sites were reported for a single gene, the identification of transcription initiation sites in plant mitochondrial genomes is challenging [15,36,42,44]. Furthermore, even when cDNAs are studied it is difficult to rule out that the identified initiation sites are processing products [42]. Using CR-RT-PCR [36], RACE and ribonuclease protection analysis [42,44], more than one transcription start sites were reported for most protein-coding genes in the Arabidopsis mtDNA (Table S2). By contrasting with those reports, we evaluated whether the RNAseq transcription profile is useful to recognize the sites of transcription start of protein-coding genes in the Arabidopsis mtDNA. The agreement between RNAseq profile and the experimentally identified promoter regions in the Arabidopsis mtDNA would be a measure of the predictive ability of RNAseq alignment to identify the transcription start sites based on steep slopes in the RNA read depth upstream of each gene.

**Figure 2:**
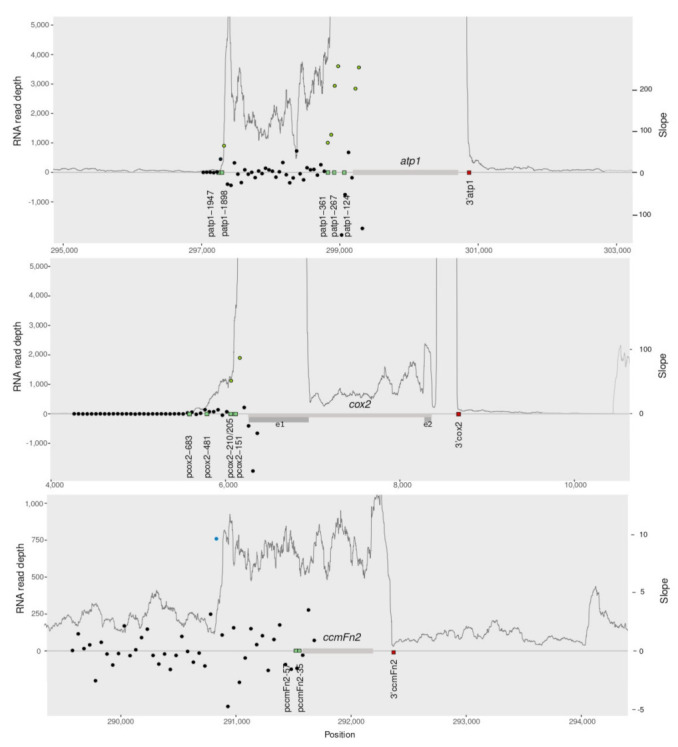
RNA read depth of three protein-coding genes and 2 kb flanking regions in the *Arabidopsis* mtDNA based on the combined data sets of the three biological samples. The slopes of the read depth (filled circles) were calculated every 50 bp in the 2 kb upstream of each gene. Gray bars represent genes and dark gray bars are exons. Green squares represent start of transcription and red squares represent 3′ termini, identified experimentally [36,42,44]. The higher values of the slopes that fall within a 100 bp window around the transcription start sites identified experimentally are shown as green circles. The higher values of the slopes found upstream of the reported transcription start sites are shown as blue circles. Below each graph, the mtDNA coordinates of the strand in which the gene is located are shown.

The combined RNAseq data sets were aligned over each of the 28 protein-coding genes or coding units (i.e., gene regions for those genes with trans-splicing introns or co-transcribed units) and their flanking sequences. Then, the slopes of the RNA read depth in the 2 kb upstream of each gene or coding unit were calculated every 50 bp. Furthermore, we analyzed spliced and unspliced versions for intron-containing genes (Figure S4). We repeated the comparisons with each of the three data sets and obtained similar results (not shown). Those regions with the highest slope values were considered putative transcription initiation sites. We then evaluated whether those putative transcription start sites based on RNAseq fell within a 100 bp window around the 85 transcription start sites identified experimentally [36,42,44] in the *Arabidopsis* mtDNA (Figure S4, Table S2). As an example, Figure 2 shows that putative transcription start sites in *atp1* and *cox2* coincide with previously reported promoters (green circles).

Overall, for 25 (89%) genes or co-transcribed units, putative transcription initiation sites match the location of at least one of the transcription start sites experimentally identified (green circles in Figure S4), although for a few genes, there are additional putative transcription start sites that do not match any known promoter, e.g., *atp6-2* and *nad3-rps12*. In two cases (*nad2e1e2* and *nad9*), some of the experimentally identified promoters are located within the transcribed region of an upstream gene (Figure S4). For the gene *nad9*, for example, four start transcription sites are located within the coding regions of the upstream genes *rps3* (pnad9-1730, pnad9-1400, pnad9-1371) and *rpl16* (pnad9-1241). In those cases, in which two genes are located in close vicinity in the genome, the transcription profile based on RNAseq data are not helpful to identify all alternative transcription start sites.

In some cases, the RNA read depth increases upstream of the locations of the transcription start sites identified experimentally (Figure S4; blue circles indicate the higher slopes located upstream of the start of transcription reported). These findings can be explained in two ways. In the first case, a yet unidentified promoter is located upstream of the reported transcription start sites, in the region exhibiting a sharp increase in read depth; for example, in *ccmFn2* (blue circle in Figure 2). In the second case, the spurious presence of a DNA sequence identical to a reported promoter is located upstream of the experimentally-identified transcription start and generates a premature increase in read depth. For example, there are sequences identical to promoters reported for other genes upstream of the dicistronic unit *nad4L-atp4* and of the gene region *nad1e1* that could be acting as spurious transcription initiation sites (Figure S4). The large number of promoters and the high recombination rate in plant mitochondria would be responsible for the random location of transcription start sites throughout the mtDNA. A search across both strands of the *Arabidopsis* mtDNA for sequences identical to the 85 transcription initiation sites described for *Arabidopsis* found 11 sequences identical to promoters randomly distributed throughout the genome (including the spurious promoters mentioned above).

For many genes multiple promoters have been described [36,42,44]. While in a few cases (e.g., *atp1*) an increase in read depth coincides with each of the transcription start sites reported (Figure 2), in other cases we only observe a match with a single promoter (e.g., *rps4*) or cannot evaluate other promoters because they are located within an upstream coding region (e.g., *nad9*) and RNAseq data is not informative. Previous studies of multiple promoters in maize using RT-PCR revealed that the genomic context influences promoter strength and suggested a role for genomic recombination in the regulation of the gene expression [55]. In addition, alternative promoters that are only active in specific nuclear backgrounds have been reported [56]. The role and strength of different promoters for a single coding region may vary across stages of the life cycle or among tissues. Here, we observed that in seedlings of *Arabidopsis* one of the promoters seems to be stronger than the alternative ones, leading to greater RNA read depth than the others.

We conclude that, if there is no overlap in the transcription bubbles of two genes or co-transcribed units, it is possible to infer the regions of transcription start sites based on RNAseq mapping, although with limited accuracy and precision. Identifying the transcription start sites in plant mitochondrial genomes may help to uncover the signals recognized by the transcription machinery and to improve our understanding of the transcription process. Given that promoters are not conserved at the sequence level, even within the same genome or in homologous genes across species [6], DNA sequence comparisons are not useful to infer transcription start sites. Thus, the patterns of RNAseq read depth may serve as a valuable start point for predicting the region of transcription start.

Noticeably, mature protein-coding mRNAs tend to have heterogeneous 5′ ends but generally well-defined 3′ ends [36]. The 3′ termini have been identified experimentally in *Arabidopsis* mitochondrial genes or gene regions [36,42,44]. We evaluated whether the transcription profile based on RNAseq is informative to determine the 3′ termini for the *Arabidopsis* protein-coding genes. Of the 27 drops in the RNA read depth observed after the stop codon of a gene or coding unit, 24 (88%) matched a 3′ termini reported (Figure S4). Those cases in which the described 3′ termini do not coincide with a drop in the read depth are mainly explained by the fact that the 3′ ends are located near or within the transcribed region of a downstream gene (e.g., *rps3-rpl16*, *rps4*; Figure S4) preventing the identification of a drop of the read depth. Read depth drops downstream of the gene regions *nad1e1*, *nad2e1e2* and *nad5e1e2* (Figure S4) could reflect the limits of the trans-splicing introns.

### 2.4. RNAseq Mapping Predicts Co-Transcription except When Genes Are Too Closely Located

The close spatial arrangement of two or more genes on the same DNA strand of plant mitochondria raises the possibility that these genes could be co-transcribed [3]. A continuously elevated RNA read depth across two genes in plant mitochondrial genomes has been interpreted as evidence of co-transcription [23,29] but this type of evidence was not validated. Here, we evaluate the reliability of the RNAseq transcription profile of *Arabidopsis* mtDNA as a predictor of co-transcribed genes by comparing those predictions with experimentally confirmed co-transcribed units based on cDNA analyses [20], Northern blots [21] or CR-RT-PCR [36].

Co-transcription of protein-coding genes has been experimentally shown for five units in the *Arabidopsis* mtDNA: *rps3-rpl16*, *nad4L-atp4*, *nad3-rps12*, *rpl5-ψrps14-cob* and *rpl2-mttB* [21,36]. The first three of those are conserved in angiosperms [57] and all five are found in the Brassicaceae. In addition, co-transcription has been proposed for other units because they were found in the same cDNA, i.e., *rps3-rpl16-nad9*, *rps4-nad2e1e2* [21,22,43]. The identification of transcription initiation sites for each gene within a co-transcribed unit demonstrates that both forms of transcription, either individual or co-transcription, are possible in some cases, such as in *rpl2-mttb* [36], *rps3-rpl16-nad9* [36,42] and *rpl5-ψrps14-cob*. Thus, the mitochondrial genetic system exhibits enough flexibility to tolerate changes in gene order and the disruption of some gene clusters [57].

**Figure 3:**
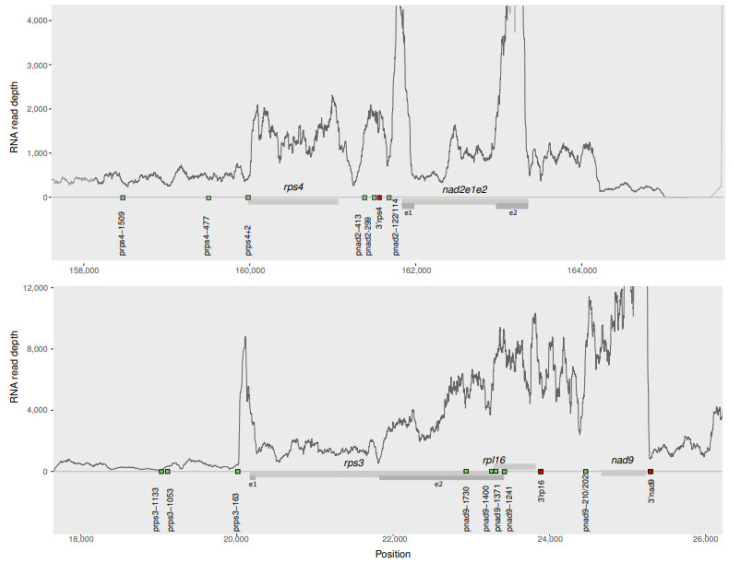
RNA read depth of protein-coding genes located at a distance < 2 kb in the *Arabidopsis* mitochondria based on the combined data sets of the three biological samples. A drop in the read depth between the genes *rps4* and *nad2e1e2* suggests independent transcription. A continuously elevated read depth across *rps3*, *rpl16* and *nad9* suggests that these genes are co-transcribed. Gray bars represent genes and dark gray bars are exons. Green and red squares represent start of transcription and 3′ termini, respectively, identified experimentally [36,42,44]. Below each graph, the mtDNA coordinates of the strand in which the genes are located are shown.

In this case, 11 pairs of genes that are located at a maximum distance of 2 kb in the *Arabidopsis* mtDNA could be potentially co-transcribed: *rps3-rpl16*, *rpl16-nad9*, *nad9-nad5e4e5*, *rps4-nad2e12*, *rpl5-ψrps14-cob*; *rpl2-mttb*, *nad1e4e5-nad5e1e2*, *atp8-nad7*, *nad3-rps12*, *nad5e3-nad4L* and *nad4L-atp4*. However, those cases in which two genes are located very close to each other (i.e., genes within the pairs *nad3-rps12* and *nad1e4e5-nad5e1e2* are separated by less than 150 bp) or even with overlapping coding regions (*rps3-rpl16*) cannot be evaluated using RNAseq data as the overlapping elevated RNA read depth of each gene precludes distinguishing independent versus co-transcription; thus, these gene pairs were not further analyzed. We aligned all RNA reads of the three data sets combined on the remaining eight pairs of genes. Three of the eight gene pairs exhibit a continuous high read depth, *rpl16-nad9*; *rpl5-ψrps14-cob* and *nad4L-atp4*, while the rest show a drop between the genes of a gene pair (Figure 3 and Figure S5).

The observations on the eight gene pairs generally agree with the experimentally confirmed co-transcribed units and independent transcribed genes reported in previous studies. Similar to *rpl5-ψrps14-cob* and *nad4L-atp4*, the continuously elevated read depth across the *rps3-rpl16-nad9* cluster (Figure 3) is consistent with the presence of these genes in a single cDNA [43]. However, given that three of the promoters described for *nad9* are located within the coding region of *rpl16* (see above and Figure S4), a continuously elevated RNA read depth between *rpl16* and *nad9* is expected even if *nad9* is independently transcribed using those promoters located in the upstream coding region. In contrast, a drop in the read depth between the gene pairs *rpl2-mttb* and *rps4-nad2_e1_2* is consistent with the independent transcription reported for *rpl2*, *rps4* and *nad2e1_2* [36,42], although there is also evidence for co-transcription of these units [22,36]. Finally, the gene pairs *nad9-nad5e4e5*, *nad5e3-nad4L* and *atp8-nad7* show a drop in the read depth within the intergenic space in agreement with their independent transcription using different transcription start sites [36,42]. Overall, mapping RNAseq data to the mitochondrial genome is useful to predict if two genes are co-transcribed only when the genes (and their promoters) are located sufficiently apart in the genome to avoid an overlap of the RNA read depths in case of independent transcription.

### 2.5. Splicing Efficiency of Cis and Trans-Splicing Introns and Those with Different Splicing Mechanisms

Intron splicing is essential for the correct expression of several mitochondrial genes and requires precise coordination with the nucleus given that the splicing machinery is encoded in the nuclear genome [16,58]. The restructuring of angiosperm mitochondrial genomes caused by recombination events can contribute to disparate evolutionary directions of the introns that interrupt coding regions [58]. Two types of intron splicing processes are present in the *Arabidopsis* mtDNA. Cis-splicing is the intramolecular ligation of exon sequences on the same precursor RNA, and trans-splicing involves the intermolecular ligation of exon sequences from different primary transcripts [3,46]. Due to their degenerate nature and the fact that most organellar introns have lost their cognate maturase factors, both cis and trans-splicing reactions of plant mitochondrial group II introns rely on the action of different catalytic enzymes, most of which are encoded by nuclear loci [16,59].

In *Arabidopsis* mtDNA, there are 23 group II introns in genes related to respiratory complexes I (*nad1*, *nad2*, *nad4*, *nad5 and nad7*) and IV (*cox2*), and in the genes *ccmFc*, *rpl2* and *rps3.* Most introns undergo cis-splicing, while trans-spliced introns are found in the genes *nad1*, *nad2* and *nad5* [58]. Group II introns exhibit a secondary structure with six double-helical domains (dI-dVI). The splicing of classical group II introns involves two sequential transesterifications. In the first, the dVI unpaired adenosine, named ‘bulging A’, is covalently joined to the intron 5′ end resulting in the formation of a lariat shaped intermediate. In the second transesterification the two exons are joined, releasing the intron lariat [60]. This splicing mechanism is known as branching pathway [16]. In an alternative group II intron splicing mechanism (termed hydrolytic pathway) the first step occurs by hydrolysis [61]. The splicing mechanisms of the introns in the *Arabidopsis* mtDNA have been previously elucidated (Table 1) enabling the comparison of the splicing efficiency based on the RNA profile of spliced and unspliced versions of genes with cis or trans-splicing considering the different types of splicing mechanisms.

In the present work, we analyzed the splicing efficiency in the *Arabidopsis* mtDNA by comparing the read depth at exon-exon and exon-intron junctions (Figure S3). The splicing efficiency (SE) was calculated as the number of RNAseq reads supporting the mature conformation (exon-exon junctions, EE) divided by the sum of RNAseq reads that support exon-exon junctions and the average number of reads that support either of the immature conformations (exon-intron junctions: EI5′ or I3′E). The splicing efficiency of introns in the *Arabidopsis* mitochondria ranges from 0.54 to 0.98 with a global average of 0.82 (Table 1). The three biological replicates show highly consistent results with averages of 0.81, 0.83 and 0.81 for the three runs ERR1665120, ERR1665119 and ERR1665115, respectively (Table S3). The splicing efficiency is variable among genes (Table 1 and Table S3). The single intron of *cox2* and the second intron of *nad4* exhibit the highest splicing efficiencies (0.98). In contrast, the *rpl2* intron shows low SE in the individual and combined analyses (0.49–0.58). Notoriously, the SE also differs among introns of those genes that contain more than one intron (Table 1 and Table S3). For example, *nad1i1* shows SE = 0.58 and *nad1i2* SE = 0.93. Thus, both the RNA read depth and the splicing efficiency are variable within and among genes (Table S3).

Comparisons between cis and trans-splicing introns do not reveal significant differences in splicing efficiency. Furthermore, no correlation in SE is observed for the different splicing mechanisms (branching versus hydrolytic pathways, or both). This indicates that the SE is not related to the splicing mechanism and it is not affected by the split of introns that now require trans-splicing. Noticeably, in all but two introns (*rps3* and *nad4_intron2*), the number of reads supporting the unspliced form of the 3′ end of the intron with the downstream exon (I3′E) is higher (up to 10-fold) than that of the 5′ end of the same intron with the upstream exon (EI5′) (Table 1). This is likely the result of the splicing process, either via the branching or hydrolytic pathway, where the junction I3′E remains for a longer period than the junction EI5′, and thus more reads support this conformation [16].

This study serves as a comparative measure of mitochondrial intron splicing in free living angiosperms. In recent years, a large number of angiosperm transcriptomes have been sequenced and await comprehensive studies to shed light on the splicing efficiency of group I and II introns present in the mitochondrial genomes of diverse plants. In addition, the splicing efficiency could be compared across different tissues and different phenological stages of a single species, or even across land plants with different lifestyles, such as free-living and parasitic species. In agreement with the results shown here, an earlier study of the tobacco transcriptome [29] found that the abundance of the exonic steady-state level was higher than that of the introns, but the splicing efficiency was not measured. These observations contrast with those in the holoparasitic plant *Lophophytum mirabile* in which similar levels of exonic and intronic RNA read depth were noted suggesting a low splicing efficiency [33]. A comparative study of diverse angiosperms would unveil the evolution of intron splicing in mitochondrial genes of plants.

## 3. Materials and Methods

### 3.1. RNA Data Sets

To analyze the transcriptional landscape of the mtDNA of *Arabidopsis*, RNA Illumina reads were downloaded from the NCBI Sequence Read Archive (SRA) under the Bioproject accession PRJEB15579 using the fastq-dump tool in the NCBI SRA Toolkit v2.9.6. The RNAseq data sets analyzed here correspond to the sequencing of total RNA treated with Ribozero extracted from three biological replicates of wild-type *A. thaliana* ecotype Columbia submitted by University of Dundee (UK). Libraries were prepared using Illumina TruSeq stranded Ribozero plant kit and paired-end sequencing was carried out with Illumina HiSeq 2000. The three RNA sequencing runs analyzed include ERR1665220 (biosample SAMEA4475146), ERR1665219 (biosample SAMEA4475145) and ERR1665215 (biosample SAMEA4475141) of 222,419,248; 213,387,956; and 222,124,790 reads of 101 bp, respectively. The quality of the three runs was assessed using FastQC version 0.10.1. Low-quality sequences and the first 12 nucleotides of each read were trimmed using Trimmomatic [62], leading to 208,850,768; 199,739,120; and 209,258,080 paired-end sequences of 89 bp for runs ERR1665220, ERR1665219 and ERR1665215, respectively. For some analyses, we pooled the three data sets of trimmed reads obtaining a total amount of 617,847,968 paired-end sequences.

After exploring the RNAseq data sets available in public databases, we selected this particular data set based on the following: (i) the extracted RNA was depleted of rRNA instead of poly-A selected, which is relevant for mitochondrial expression studies given that poly-A tracts represent a signal for RNA degradation in plant mitochondria [14,63,64]; (ii) the volume of the three data sets combined is large and comparable to recent large-scale studies on angiosperm mitochondrial expression [23,33]; (iii) the data are of high quality as reported by FastQC; and (iv) they were obtained from a wild-type plant of *A.* *thaliana* ecotype Columbia, for which a curated mtDNA is available.

### 3.2. RNA Read Alignments

The RNA alignments were performed using Bowtie2 [65] with the sensitive, end-to-end mode, the no-discordant, no-mixed and R = 10 options, and the fr and nofw options to enforce strand-specific alignment. To avoid the mismapping of RNA reads derived from chloroplast genes, the five plastid-like regions present in the *Arabidopsis* mtDNA were masked using Beedtools v2.29.2 [66]. Individual and combined RNAseq data sets were aligned to three reference DNA sequences: (i) both strands of the mtDNA of *A.* *thaliana* ecotype Columbia (BK010421); (ii) all intact protein-coding genes annotated in the *Arabidopsis* mtDNA with these sequences extended by 100 bp at each end to obtain a good read depth at the ends of the genes; (iii) for intron-bearing genes, we also used as reference a spliced version extended by 100 bp at each end. The normalized read depth of each protein-coding gene was calculated as RPKM [52] by diving the product of the number of reads mapped to a gene and the scaling factor (109) by the length of the gene in base pairs and by the total number of reads mapped to all protein-coding genes in *Arabidopsis* mtDNA.

### 3.3. Initiation and Termination of Transcription Analysis

For the transcription start site prediction, the slopes of the RNA read depth were calculated in 50 bp windows within the 2 kb region upstream of each gene. For this, the alignments of the combined RNAseq data sets over the unspliced and spliced versions of each gene were analyzed. The location of the experimentally verified transcription start sites and 3′ termini on the *Arabidopsis* mtDNA was compared to that of the higher slope values (putative transcription start sites) using BEDtools [66] and to the drops in RNA read depth downstream of each gene, respectively.

### 3.4. Co-Transcription Analysis

Gene pairs located up to 2 kb apart in the *Arabidopsis* mtDNA were subjected to co-transcription analyses. For this, we examined the alignments of the combined RNAseq data sets over the unspliced and spliced versions of each gene pair to distinguish whether the read depth was continuously elevated or showed a drop in between the gene pair.

### 3.5. Splicing Efficiency

The splicing efficiency was calculated by applying a method developed for yeast nuclear genes [67] except for the correction factor that they used for polyadenylated samples that was not used here. We analyzed the RNA alignments of the spliced and unspliced versions of the intron-containing genes described above. Considering 30 bp at each side of the splicing site, the number of reads that mapped to the exon-intron junction (EI5’), the intron-exon junction (I3′E) and the exon-exon junction (EE) were recorded. The splicing efficiency was calculated according to the following formula SE = N_EE_/(N_Total_), where N_Total_ = N_EE_ + 0.5 × N_EI5_ + 0.5 × N_I3E_. One-way Anova (Rstudio) was used to compare the splicing efficiency of introns with different mechanisms of splicing (branching and hydrolytic pathways, or both) or alternative types of splicing (cis and trans-splicing).

## Figures and Tables

**Table 1:** Splicing efficiency of introns in the *Arabidopsis* mtDNA based on the combined RNAseq data sets of the three biological samples.

	Cis or Trans-Splicing ^a^	Splicing Mechanism ^b^	RNA Read Depth			I3′E/EI5′	Splicing Efficiency (SE)
			EI5′ ^c^	I3′E ^d^	EE ^e^		
ccmFci	*Cis-splicing*	Branching pathway	192	356	1149	1.85	0.81
cox2i	*Cis-splicing*	Branching pathway	129	282	12,433	2.19	0.98
nad1i1	*Trans-splicing*	Hydrolytic pathway	180	1197	944	6.65	0.58
nad1i2	*Cis-splicing*	Hydrolytic pathway	151	174	2322	1.15	0.93
nad1i3	*Trans-splicing*	Both pathways were reported	187	945	3210	5.05	0.85
nad1i4	*Cis-splicing*	Branching pathway	240	1280	1982	5.33	0.72
nad2i1	*Cis-splicing*	Hydrolytic pathway	141	437	919	3.10	0.76
nad2i2	*Trans-splicing*	Branching pathway	54	476	1958	8.81	0.88
nad2i3	*Cis-splicing*	Branching pathway	59	581	1471	9.85	0.82
nad2i4	*Cis-splicing*	Branching pathway	389	460	2688	1.18	0.86
nad4i1	*Cis-splicing*	Both pathways were reported	480	633	2498	1.32	0.82
nad4i2	*Cis-splicing*	Hydrolytic pathway	144	125	6924	0.87	0.98
nad4i3	*Cis-splicing*	Branching pathway	163	468	8402	2.87	0.96
nad5i1	*Cis-splicing*	Branching pathway	278	817	1452	2.94	0.73
nad5i4	*Cis-splicing*	Branching pathway	189	1031	17,008	5.46	0.97
nad7i1	*Cis-splicing*	Hydrolytic pathway	75	135	639	1.80	0.86
nad7i2	*Cis-splicing*	Branching pathway	177	1379	1169	7.79	0.60
nad7i3	*Cis-splicing*	Branching pathway	104	332	4001	3.19	0.95
nad7i4	*Cis-intron*	Branching pathway	209	304	2685	1.45	0.91
rpl2i	*Cis-splicing*	Both pathways were reported	122	685	465	5.61	0.54
rps3i	*Cis-splicing*	Branching pathway	496	211	734	0.43	0.67

^a^ The trans-splicing introns *nad5i2* and *nad5i3* were not analyzed because the *nad5* exon 3 is too short. ^b^ Information taken from Gualberto et al., 2015 [18]. ^c^ Number of reads that support the conformation exon-5′intron. ^d^ Number of reads that support the conformation 3′intron-exon. ^e^ Number of reads that support the conformation exon-exon.

## Data Availability

Not applicable.

## Supplementary Materials

The following are available online at https://www.mdpi.com/article/10.3390/cells10082054/s1, Figure S1: RNA read depth of the *Arabidopsis* mtDNA based on the combined data sets of the three biological samples, Figure S2: The frequency distribution of RNAseq read depth calculated based on 100-bp windows across both strands of the entire *Arabidopsis* mitochondrial genome, Figure S3: RNA read depth of the protein-coding genes in the *Arabidopsis* mtDNA based on the combined data sets of the three biological samples, Figure S4: RNA read depth of protein-coding genes and 2 kb flanking regions in the Arabidopsis mtDNA based on the combined data sets of the three biological samples, Figure S5: RNA read depth of protein-coding genes located at a distance < 2 kb in the *Arabidopsis* mtDNA based on the combined data sets of the three biological samples, Table S1: Average RNA read depth and RPKM of the CDS of all protein-coding genes in *Arabidopsis* mtDNA, Table S2: Transcription start sites and 3’ ends identified experimentally for protein-coding sequences in *Arabidopsis* mitochondria, Table S3: Splicing efficiency of introns in the *Arabidopsis* mtDNA calculated using reads mapped to exon-intron versus exon-exon junctions based on individual RNAseq data sets of the three biological samples.

## References

[1] Gray M.W., Burger G., Lang B.F. (2001). The origin and early evolution of mitochondria. Genome Biol..

[2] Putintseva Y.A., Bondar E.I., Simonov E.P., Sharov V.V., Oreshkova N.V., Kuzmin D.A., Konstantinov Y.M., Shmakov V.N., Belkov V.I., Sadovsky M.G. (2020). Siberian larch (*Larix sibirica* Ledeb.) mitochondrial genome assembled using both short and long nucleotide sequence reads is currently the largest known mitogenome. BMC Genom..

[3] Unseld M., Marienfeld J.R., Brandt P., Brennicke A. (1997). The mitochondrial genome of *Arabidopsis thaliana* contains 57 genes in 366,924 nucleotides. Nat. Genet..

[4] Kubo T., Nishizawa S., Sugawara A., Itchoda N., Estiati A., Mikami T. (2000). The complete nucleotide sequence of the mitochondrial genome of sugar beet (*Beta vulgaris* L.) reveals a novel gene for tRNACys(GCA). Nucleic Acids Res..

[5] Notsu Y., Masood S., Nishikawa T., Kubo N., Akiduki G., Nakazono M., Hirai A., Kadowaki K. (2002). The complete sequence of the rice (*Oryza sativa* L.) mitochondrial genome: Frequent DNA sequence acquisition and loss during the evolution of flowering plants. Mol. Genet. Genom..

[6] Handa H. (2003). The complete nucleotide sequence and RNA editing content of the mitochondrial genome of rapeseed (*Brassica napus* L.): Comparative analysis of the mitochondrial genomes of rapeseed and Arabidopsis thaliana. Nucleic Acids Res..

[7] Clifton S.W., Minx P., Fauron C.M.-R., Gibson M., Allen J.O., Sun H., Thompson M., Barbazuk W., Kanuganti S., Tayloe C. (2004). Sequence and Comparative Analysis of the Maize NB Mitochondrial Genome. Plant Physiol..

[8] Satoh M., Kubo T., Nishizawa S., Estiati A., Itchoda N., Mikami T. (2004). The cytoplasmic male-sterile type and normal type mitochondrial genomes of sugar beet share the same complement of genes of known function but differ in the content of expressed ORFs. Mol. Genet. Genom..

[9] Ogihara Y., Yamazaki Y., Murai K., Kanno A., Terachi T., Shiina T., Miyashita N., Nasuda S., Nakamura C., Mori N. (2005). Structural dynamics of cereal mitochondrial genomes as revealed by complete nucleotide sequencing of the wheat mitochondrial genome. Nucleic Acids Res..

[10] Sugiyama Y., Watase Y., Nagase M., Makita N., Yagura S., Hirai A., Sugiura M. (2004). The complete nucleotide sequence and multipartite organization of the tobacco mitochondrial genome: Comparative analysis of mitochondrial genomes in higher plants. Mol. Genet. Genom..

[11] Gandini C.L., Garcia L.E., Abbona C., Sanchez-Puerta M.V. (2019). The complete organelle genomes of *Physochlaina orientalis*: Insights into short sequence repeats across seed plant mitochondrial genomes. Mol. Phylogenetics Evol..

[12] Kubo T., Newton K.J. (2008). Angiosperm mitochondrial genomes and mutations. Mitochondrion.

[13] Kühn K., Gualberto J.M., Maréchal-Drouard L. (2012). Chapter Nine-Recombination in the Stability, Repair and Evolution of the Mitochondrial Genome. Advances in Botanical Research.

[14] Holec S., Lange H., Kühn K., Alioua M., Börner T., Gagliardi D. (2006). Relaxed Transcription in Arabidopsis Mitochondria Is Counterbalanced by RNA Stability Control Mediated by Polyadenylation and Polynucleotide Phosphorylase. Mol. Cell. Biol..

[15] Binder S., And K.S., Stoll B. (2016). Maturation of 5’ ends of plant mitochondrial RNAs. Physiol. Plant..

[16] Brown G.G., Francs-Small C.C.D., Ostersetzer-Biran O. (2014). Group II intron splicing factors in plant mitochondria. Front. Plant Sci..

[17] Cohen S., Zmudjak M., Francs-Small C.C.D., Malik S., Shaya F., Keren I., Belausov E., Many Y., Brown G.G., Small I. (2014). nMAT4, a maturase factor required fornad1pre-mRNA processing and maturation, is essential for holocomplex I biogenesis in *Arabidopsis* mitochondria. Plant J..

[18] Gualberto J.M., Le Ret M., Beator B., Kühn K. (2015). The RAD52-like protein ODB1 is required for the efficient excision of two mitochondrial introns spliced via first-step hydrolysis. Nucleic Acids Res..

[19] Shevtsov S., Nevo-Dinur K., Faigon L., Sultan L.D., Zmudjak M., Markovits M., Ostersetzer-Biran O. (2019). Correction: Control of organelle gene expression by the mitochondrial transcription termination factor mTERF22 in *Arabidopsis thaliana* plants. PLoS ONE.

[20] Giegé P., Hoffmann M., Binder S., Brennicke A. (2000). RNA degradation buffers asymmetries of transcription in *Arabidopsis* mitochondria. EMBO Rep..

[21] Brandt P., Sünkel S., Unseld M., Brennicke A., Knoop V. (1992). The nad4L gene is encoded between exon c of nad5 and orf25 in the Arabidopsis mitochondrial genome. Mol. Genet. Genom..

[22] Lippok B., Brennicke A., Unseld M. (1996). The rps4-Gene Is Encoded Upstream of the nad2-Gene in *Arabidopsis* Mitochondria. Biol. Chem. Hoppe-Seyler.

[23] Varré J.-S., D’Agostino N., Touzet P., Gallina S., Tamburino R., Cantarella C., Ubrig E., Cardi T., Drouard L., Gualberto J.M. (2019). Complete Sequence, Multichromosomal Architecture and Transcriptome Analysis of the *Solanum tuberosum* Mitochondrial Genome. Int. J. Mol. Sci..

[24] Tsujimura M., Kaneko T., Sakamoto T., Kimura S., Shigyo M., Yamagishi H., Terachi T. (2019). Multichromosomal structure of the onion mitochondrial genome and a transcript analysis. Mitochondrion.

[25] Kwasniak-Owczarek M., Kazmierczak U., Tomal A., Mackiewicz P., Janska H. (2019). Deficiency of mitoribosomal S10 protein affects translation and splicing in *Arabidopsis* mitochondria. Nucleic Acids Res..

[26] Sun Y., Xie M., Xu Z., Chan K.C., Zhong J.Y., Fan K., Wong-Bajracharya J., Lam H.-M., Lim B.L. (2020). Differential RNA Editing and Intron Splicing in Soybean Mitochondria During Nodulation. Int. J. Mol. Sci..

[27] Lima M.S., Smith D.R. (2017). Pervasive, Genome-Wide Transcription in the Organelle Genomes of Diverse Plastid-Bearing Protists. G3 Genes Genomes Genet..

[28] Fujii S., Toda T., Kikuchi S., Suzuki R., Yokoyama K., Tsuchida H., Yano K., Toriyama K. (2011). Transcriptome map of plant mitochondria reveals islands of unexpected transcribed regions. BMC Genom..

[29] Grimes B.T., Sisay A.K., Carroll H.D., Cahoon A.B. (2014). Deep sequencing of the tobacco mitochondrial transcriptome reveals expressed ORFs and numerous editing sites outside coding regions. BMC Genom..

[30] Wu Z., Stone J.D., Štorchová H., Sloan D.B. (2015). High transcript abundance, RNA editing, and small RNAs in intergenic regions within the massive mitochondrial genome of the angiosperm *Silene noctiflora*. BMC Genom..

[31] Silva S.R., Alvarenga D.O., Aranguren Y., Penha H., Fernandes C.C., Pinheiro D.G., Oliveira M.T., Michael T.P., Miranda V.F.O., Varani A.M. (2017). The mitochondrial genome of the terrestrial carnivorous plant *Utricularia reniformis* (Lentibulariaceae): Structure, comparative analysis and evolutionary landmarks. PLoS ONE.

[32] Evans D.L., Hlongwane T.T., Joshi S.V., Pachón D.M.R. (2019). The sugarcane mitochondrial genome: Assembly, phylogenetics and transcriptomics. PeerJ.

[33] Garcia L.E., Edera A.A., Palmer J.D., Sato H., Sanchez-Puerta M.V. (2021). Horizontal gene transfers dominate the functional mitochondrial gene space of a holoparasitic plant. New Phytol..

[34] Grewe F., Edger P.P., Keren I., Sultan L., Pires J.C., Ostersetzer-Biran O., Mower J.P. (2014). Comparative analysis of 11 Brassicales mitochondrial genomes and the mitochondrial transcriptome of *Brassica oleracea*. Mitochondrion.

[35] Shtratnikova V.Y., Schelkunov M.I., Penin A.A., Logacheva M.D. (2020). Mitochondrial genome of the nonphotosynthetic mycoheterotrophic plant *Hypopitys monotropa*, its structure, gene expression and RNA editing. PeerJ.

[36] Forner J., Weber B., Thuss S., Wildum S., Binder S. (2007). Mapping of mitochondrial mRNA termini in *Arabidopsis thaliana*: T-elements contribute to 5′ and 3′ end formation. Nucleic Acids Res..

[37] Zhao P., Wang F., Li N., Shi D.-Q., Yang W.-C. (2020). Pentatricopeptide repeat protein MID1 modulates nad2 intron 1 splicing and *Arabidopsis* development. Sci. Rep..

[38] Zmudjak M., Shevtsov S., Sultan L.D., Keren I., Ostersetzer-Biran O. (2017). Analysis of the Roles of the *Arabidopsis* nMAT2 and PMH2 Proteins Provided with New Insights into the Regulation of Group II Intron Splicing in Land-Plant Mitochondria. Int. J. Mol. Sci..

[39] de Longevialle A.F., Meyer E., Andrés C., Taylor N.L., Lurin C., Millar A.H., Small I.D. (2007). The Pentatricopeptide Repeat Gene OTP43 Is Required for trans-Splicing of the Mitochondrial nad1 Intron 1 in *Arabidopsis thaliana*. Plant Cell.

[40] Arrieta-Montiel M.P., Shedge V., Davila J., Christensen A.C., Mackenzie S.A. (2009). Diversity of the Arabidopsis Mitochondrial Genome Occurs via Nuclear-Controlled Recombination Activity. Genetics.

[41] Hedtke B., Börner T., Weihe A. (1997). Mitochondrial and Chloroplast Phage-Type RNA Polymerases in Arabidopsis. Science.

[42] Kühn K., Richter U., Meyer E., Delannoy E., de Longevialle A.F., O’Toole N., Börner T., Millar A.H., Small I., Whelan J. (2009). Phage-Type RNA Polymerase RPOTmp Performs Gene-Specific Transcription in Mitochondria of *Arabidopsis thaliana*. Plant Cell.

[43] Giegé P., Brennicke A. (1999). RNA editing in *Arabidopsis* mitochondria effects 441 C to U changes in ORFs. Proc. Natl. Acad. Sci. USA.

[44] Kühn K., Weihe A., Börner T. (2005). Multiple promoters are a common feature of mitochondrial genes in *Arabidopsis*. Nucleic Acids Res..

[45] Wu Z., Hu K., Yan M., Song L., Wen J., Ma C., Shen J., Fu T., Yi B., Tu J. (2019). Mitochondrial genome and transcriptome analysis of five alloplasmic male-sterile lines in *Brassica juncea*. BMC Genom..

[46] Sloan D.B., Wu Z., Sharbrough J. (2018). Correction of Persistent Errors in *Arabidopsis* Reference Mitochondrial Genomes. Plant Cell.

[47] Kubo T., Mikami T. (2007). Organization and variation of angiosperm mitochondrial genome. Physiol. Plant..

[48] Forner J., Hölzle A., Jonietz C., Thuss S., Schwarzländer M., Weber B., Meyer R.C., Binder S. (2008). Mitochondrial mRNA Polymorphisms in Different *Arabidopsis* Accessions. Plant Physiol..

[49] Qiu Y., Filipenko S.J., Darracq A., Adams K.L. (2014). Expression of a transferred nuclear gene in a mitochondrial genome. Curr. Plant Biol..

[50] Planchard N., Bertin P., Quadrado M., Dargel-Graffin C., Hatin I., Namy O., Mireau H. (2018). The translational landscape of *Arabidopsis* mitochondria. Nucleic Acids Res..

[51] Marienfeld J., Unseld M., Brandt P., Brennicke A. (1996). Genomic Recombination of the Mitochondrial atp6 Gene in *Arabidopsis thaliana* at the Protein Processing Site Creates Two Different Presequences. DNA Res..

[52] Mortazavi A., Williams B.A., McCue K., Schaeffer L., Wold B. (2008). Mapping and quantifying mammalian transcriptomes by RNA-Seq. Nat. Methods.

[53] Binder S., Brennicke A. (2003). Gene expression in plant mitochondria: Transcriptional and post–transcriptional control. Philos. Trans. R. Soc. B Biol. Sci..

[54] Raczynska K.D., Le Ret M., Rurek M., Bonnard G., Augustyniak H., Gualberto J.M. (2006). Plant mitochondrial genes can be expressed from mRNAs lacking stop codons. FEBS Lett..

[55] Gagliardi D., Gualberto J.M., Day D.A., Millar A.H., Whelan J. (2004). Gene Expression in Higher Plant Mitochondria. Plant Mitochondria: From Genome to Function.

[56] Newton K.J., Walbot V. (1985). Maize mitochondria synthesize organ-specific polypeptides. Proc. Natl. Acad. Sci. USA.

[57] Gagliardi D., Binder S. (2018). Expression of the Plant Mitochondrial Genome. Annual Plant Reviews Online.

[58] Bonen L. (2008). Cis- and trans-splicing of group II introns in plant mitochondria. Mitochondrion.

[59] Schmitz-Linneweber C., Lampe M.-K., Sultan L.D., Ostersetzer-Biran O. (2015). Organellar maturases: A window into the evolution of the spliceosome. Biochim. et Biophys. Acta (BBA)-Bioenerg..

[60] Lambowitz A.M., Zimmerly S. (2010). Group II Introns: Mobile Ribozymes that Invade DNA. Cold Spring Harb. Perspect. Biol..

[61] Podar M., Chu V.T., Pyle A.M., Perlman P.S. (1998). Group II intron splicing in vivo by first-step hydrolysis. Nat. Cell Biol..

[62] Bolger A.M., Lohse M., Usadel B. (2014). Trimmomatic: A flexible trimmer for Illumina sequence data. Bioinformatics.

[63] Kuhn J., Tengler U., Binder S. (2001). Transcript Lifetime Is Balanced between Stabilizing Stem-Loop Structures and Degradation-Promoting Polyadenylation in Plant Mitochondria. Mol. Cell. Biol..

[64] Hirayama T., Matsuura T., Ushiyama S., Narusaka M., Kurihara Y., Yasuda M., Ohtani M., Seki M., Demura T., Nakashita H. (2013). A poly(A)-specific ribonuclease directly regulates the poly(A) status of mitochondrial mRNA in Arabidopsis. Nat. Commun..

[65] Langmead B., Trapnell C., Pop M., Salzberg S.L. (2009). Ultrafast and memory-efficient alignment of short DNA sequences to the human genome. Genome Biol..

[66] Quinlan A.R., Hall I.M. (2010). BEDTools: A flexible suit of utilities for comparing genomic features. Bioinformatics.

[67] Xia X. (2020). RNA-Seq approach for accurate characterization of splicing efficiency of yeast introns. Methods.

