# Supplementary material for: Transcriptional Landscape and Splicing Efficiency in Arabidopsis Mitochondria
Source: Cells. 2021 Aug 11;10(8):2054. doi: 10.3390/cells10082054 (PMC8392254; doi:10.3390/cells10082054)
Supplement: Supplementary file 1 [file cells-10-02054-s001.zip › FigS1.pdf]

Fig. S1. RNA read depth of the *Arabidopsis* mtDNA based on the combined data sets of the three biological samples. The curves plot the number of RNA reads mapped to each nucleotide position. The number of reads that correspond to left-to-right transcription and vice versa are represented by green and orange curves on the positive and negative y-axes, respectively. Green and orange boxes represent genes transcribed left to-right and vice versa, respectively. Light green and light orange regions are introns. Green and red squares represent start of transcription and 3' termini, respectively, identified experimentally [36, 42, 44]. Plastid-derived regions (MTPTs) were masked and are depicted with purple boxes. The orf164 (blue box) is transcribed from left to right. Pseudogenes are shown as a dark grey bar and depicted with ψ.

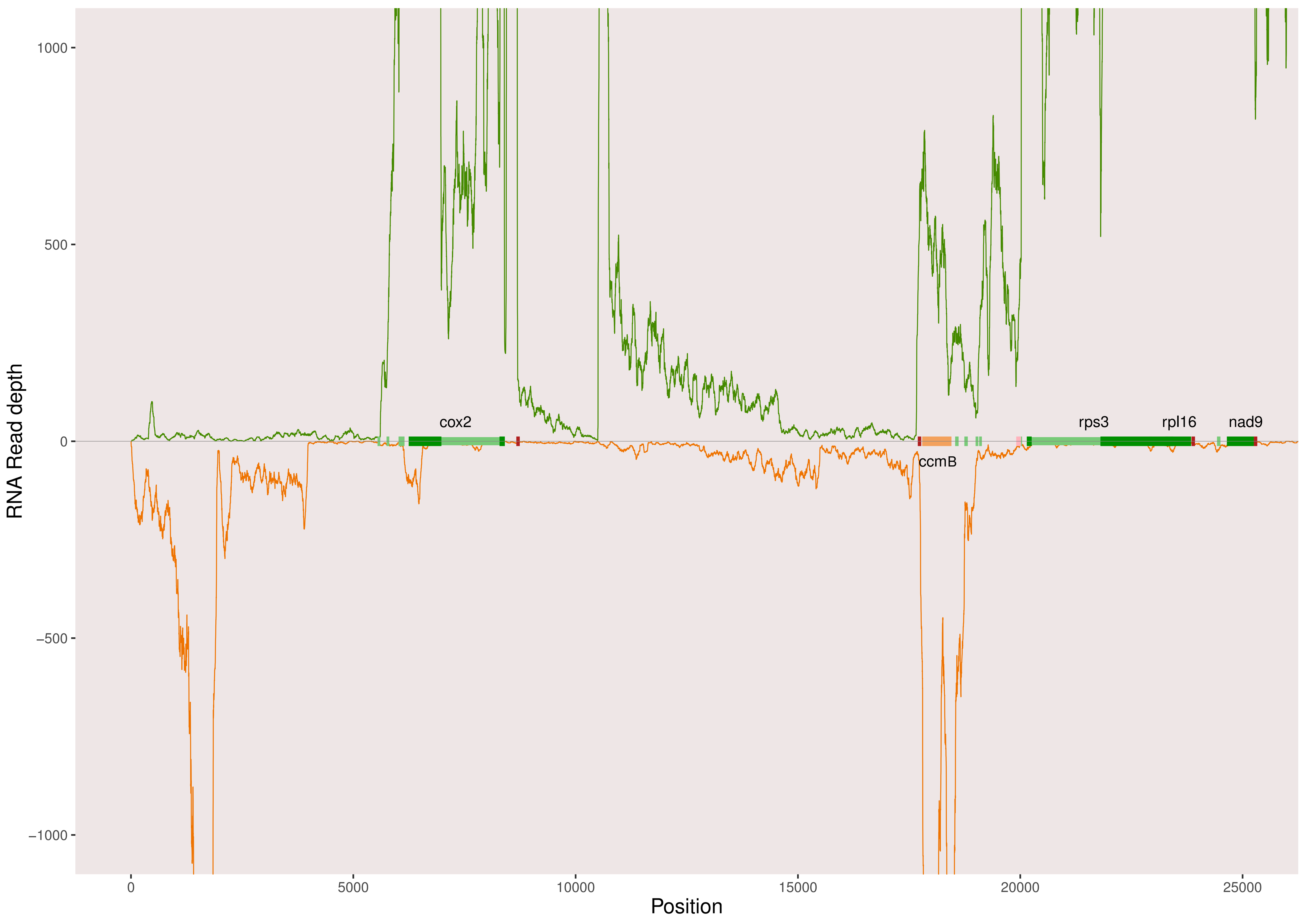

RNA Read depth
1000
500
0
−500
−1000
cox2
ccmB
rps3
rpl16
nad9
0
5000
10000
15000
20000
25000
Position

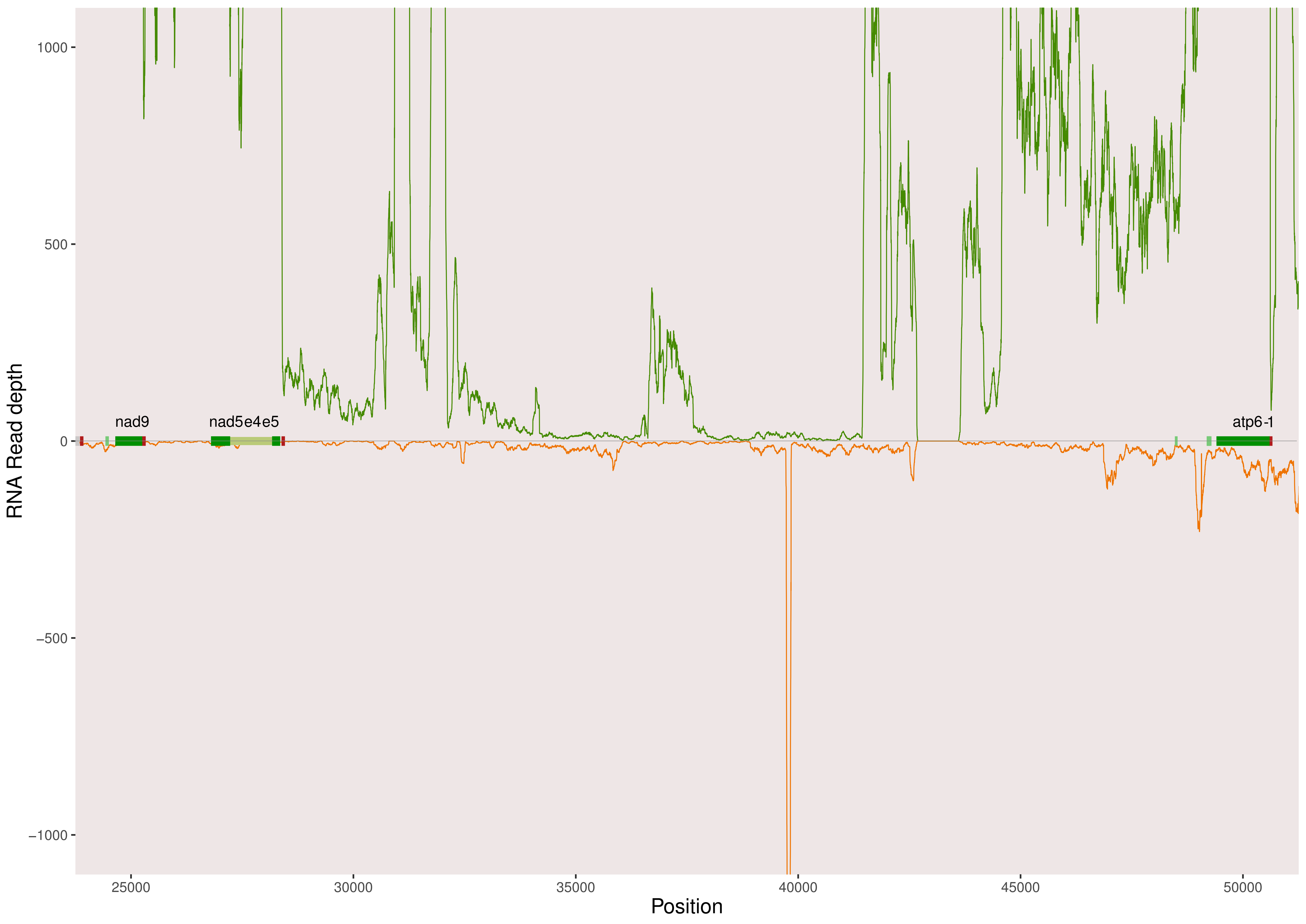

RNA Read depth
1000
500
0
−500
−1000
nad9
nad5e4e5
atp6-1
25000
30000
35000
40000
45000
50000
Position

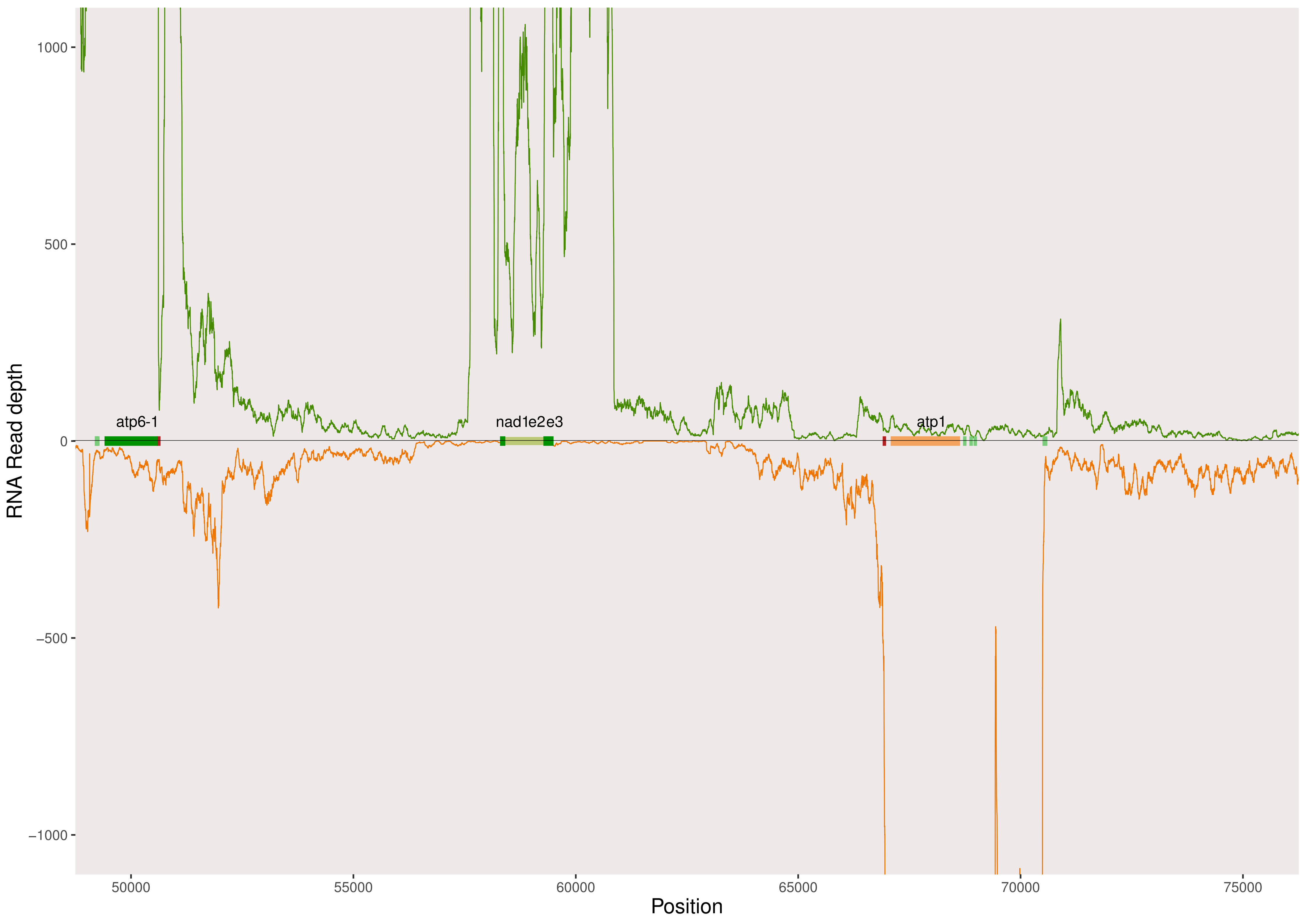
RNA Read depth
1000
500
0
−500
−1000
atp6-1
nad1e2e3
atp1
50000
55000
60000
65000
70000
75000
Position

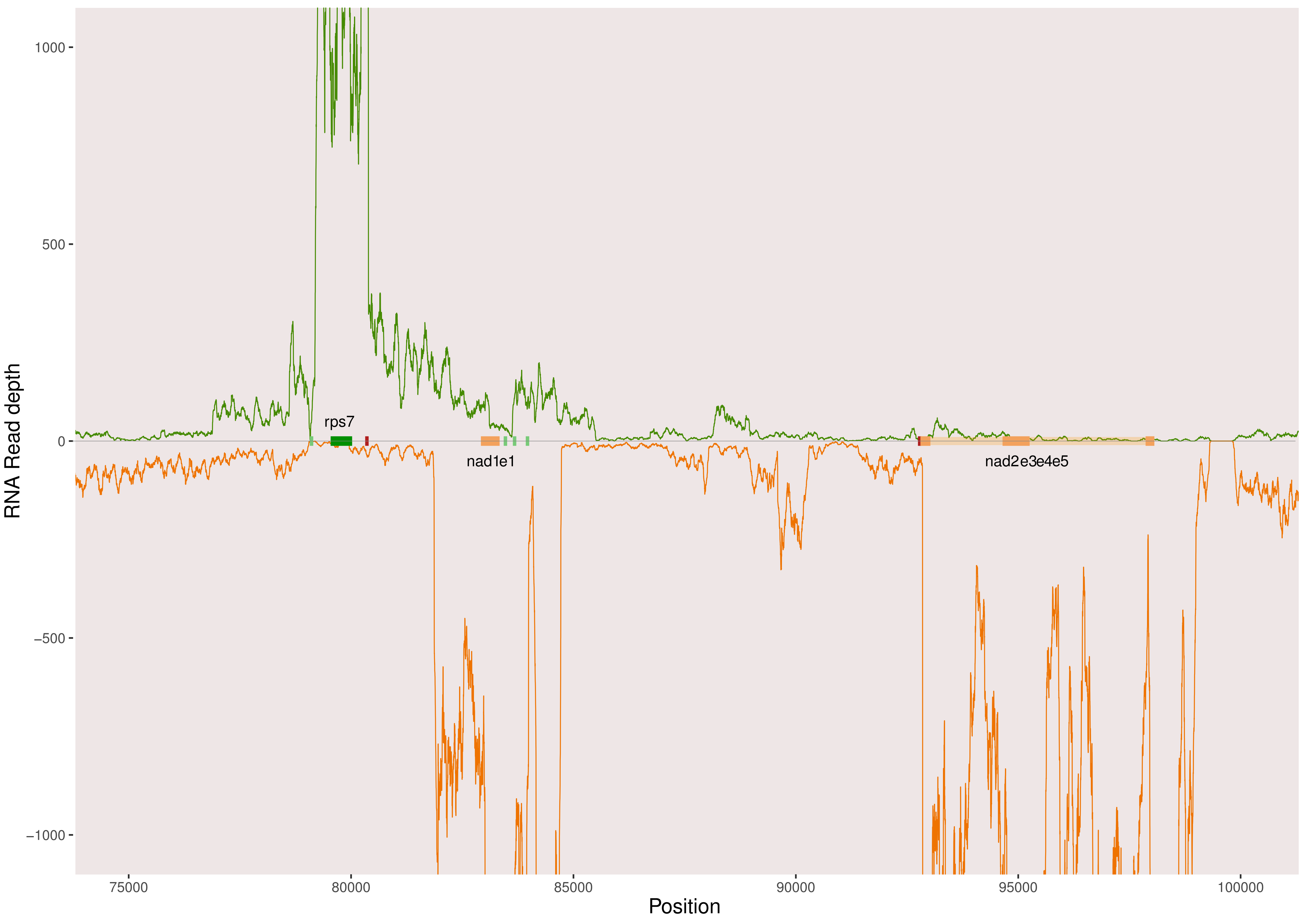

1000
500
0
−500
−1000
RNA Read depth
rps7
nad1e1
nad2e3e4e5
75000
80000
85000
90000
95000
100000
Position

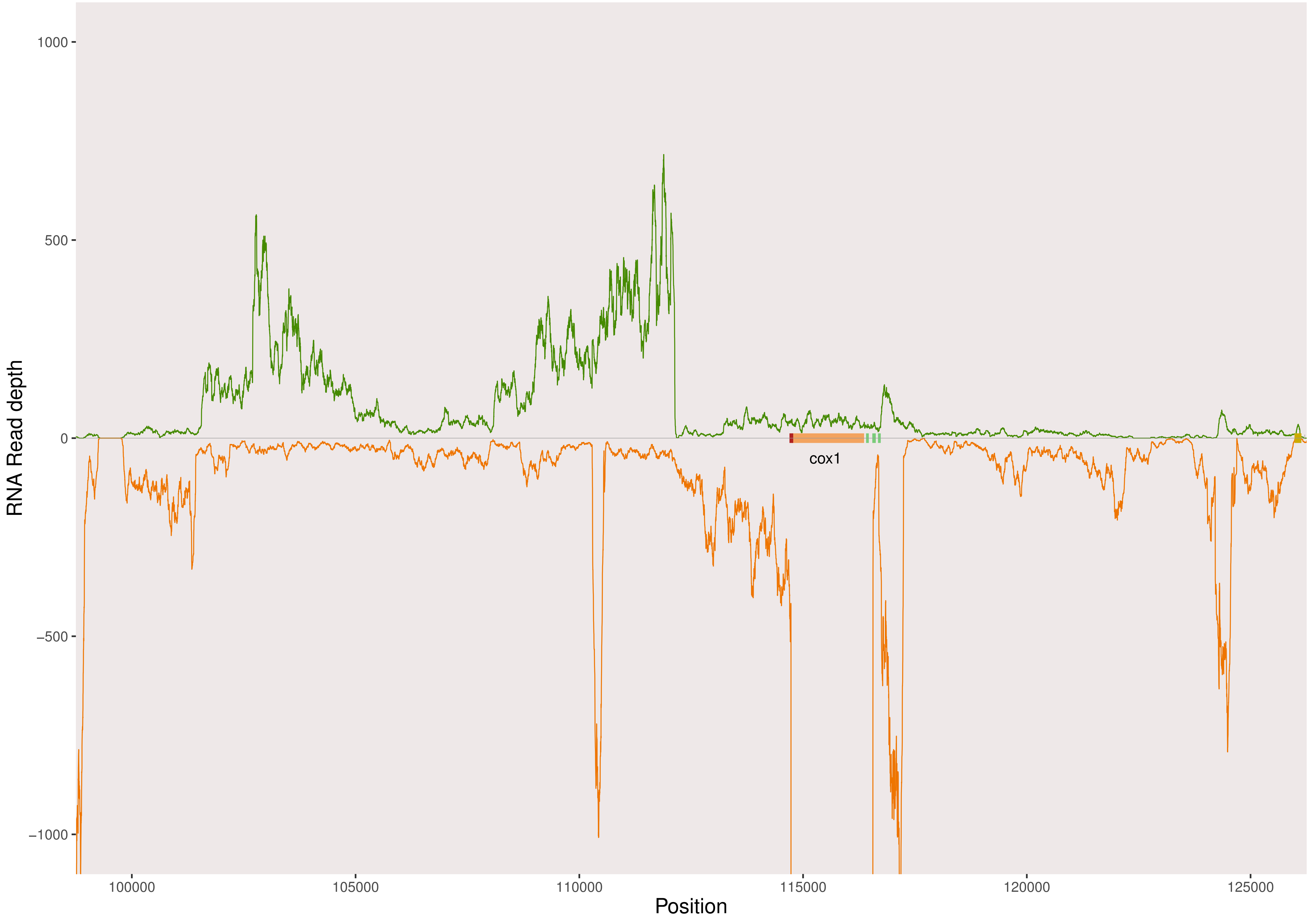

1000
500
0
−500
−1000
RNA Read depth
cox1
100000
105000
110000
115000
120000
125000
Position

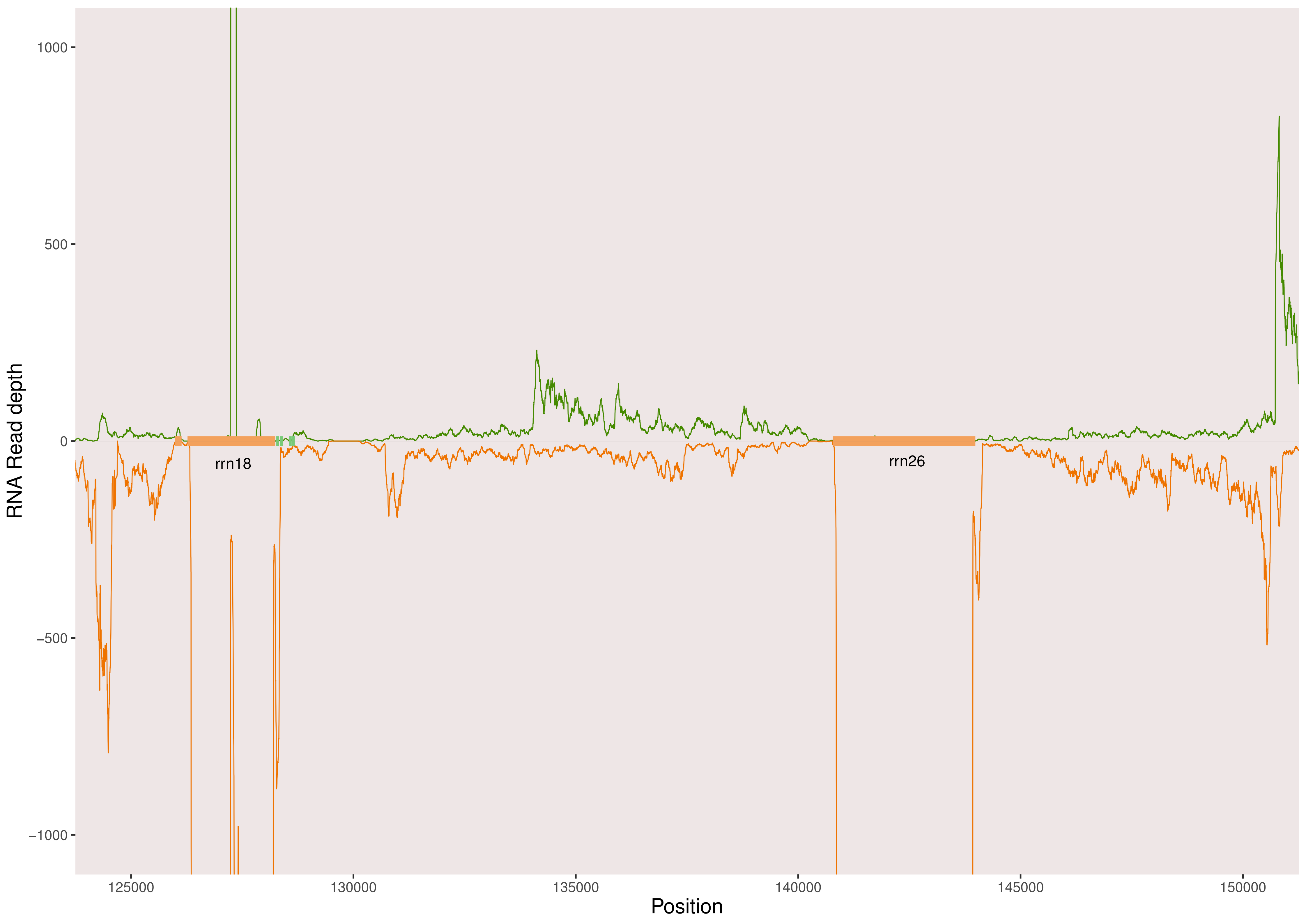

1000
500
0
−500
−1000
RNA Read depth
rrn18
rrn26
125000
130000
135000
140000
145000
150000
Position

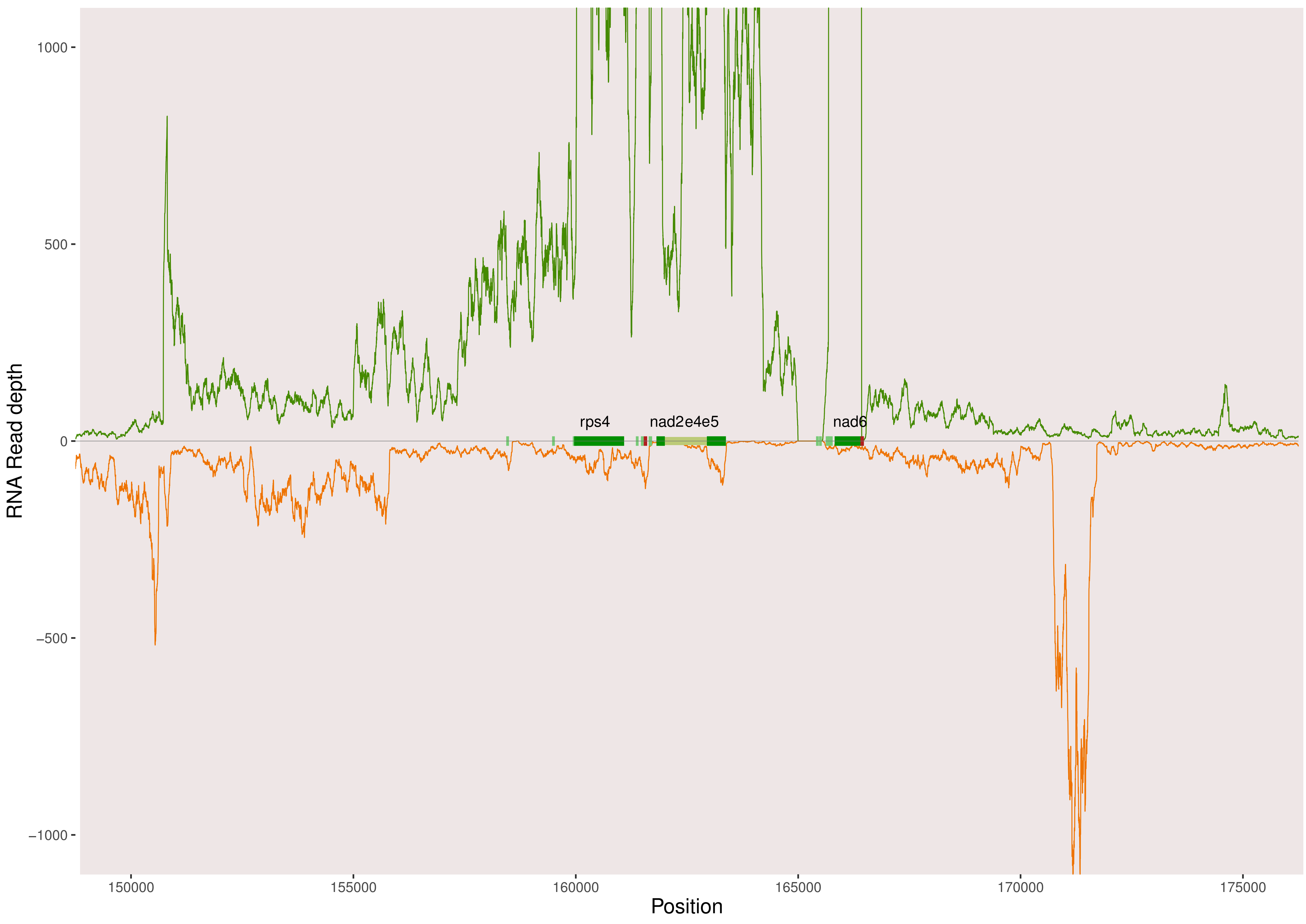

RNA Read depth
1000
500
0
−500
−1000
rps4
nad2e4e5
nad6
150000
155000
160000
165000
170000
175000
Position

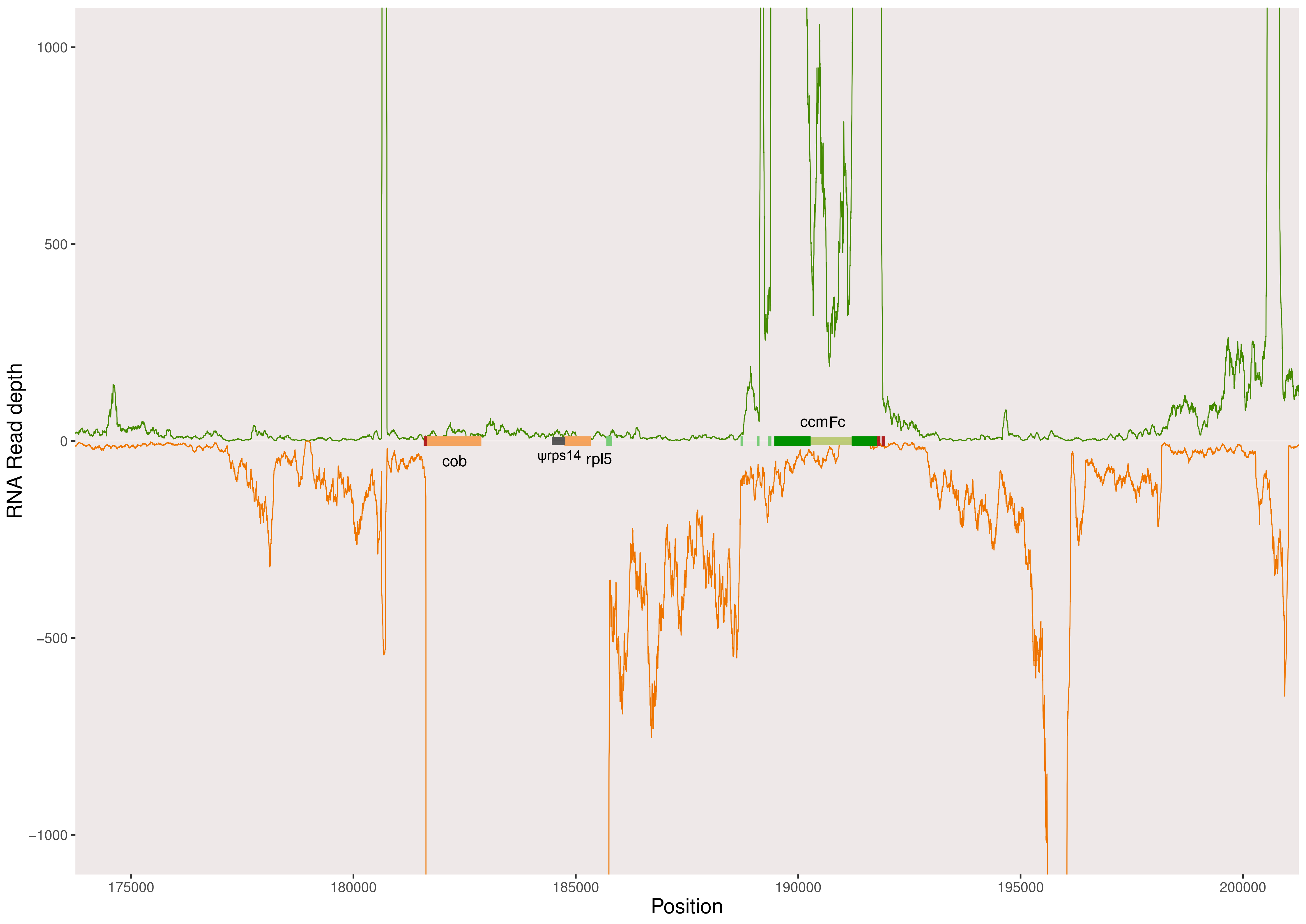

RNA Read depth
Position
1000
500
0
−500
−1000
175000
180000
185000
190000
195000
200000
cob
ψrps14
rpl5
ccmFc

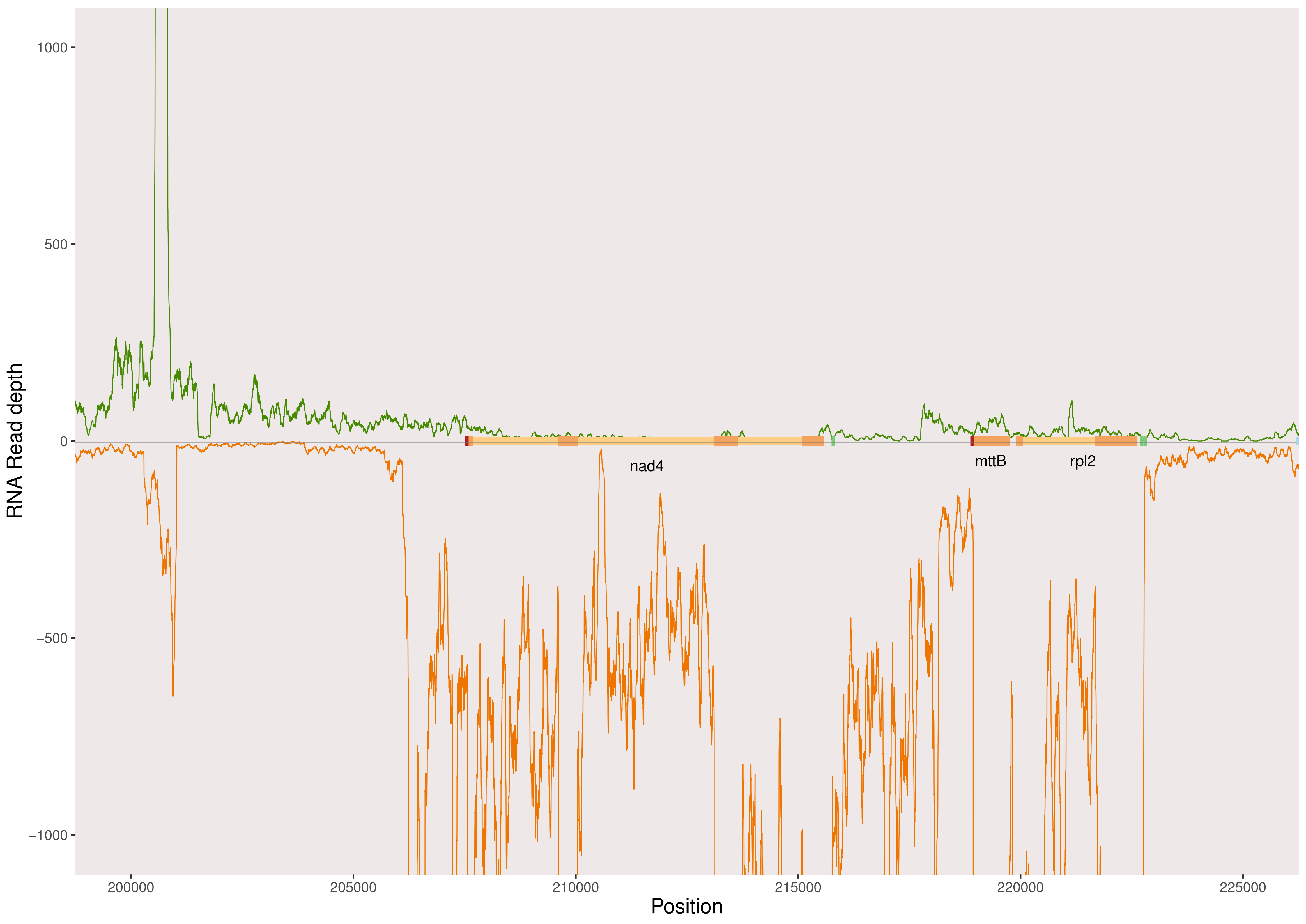

RNA Read depth
1000
500
0
−500
−1000
nad4
mttB
rpl2
200000
205000
210000
215000
220000
225000
Position

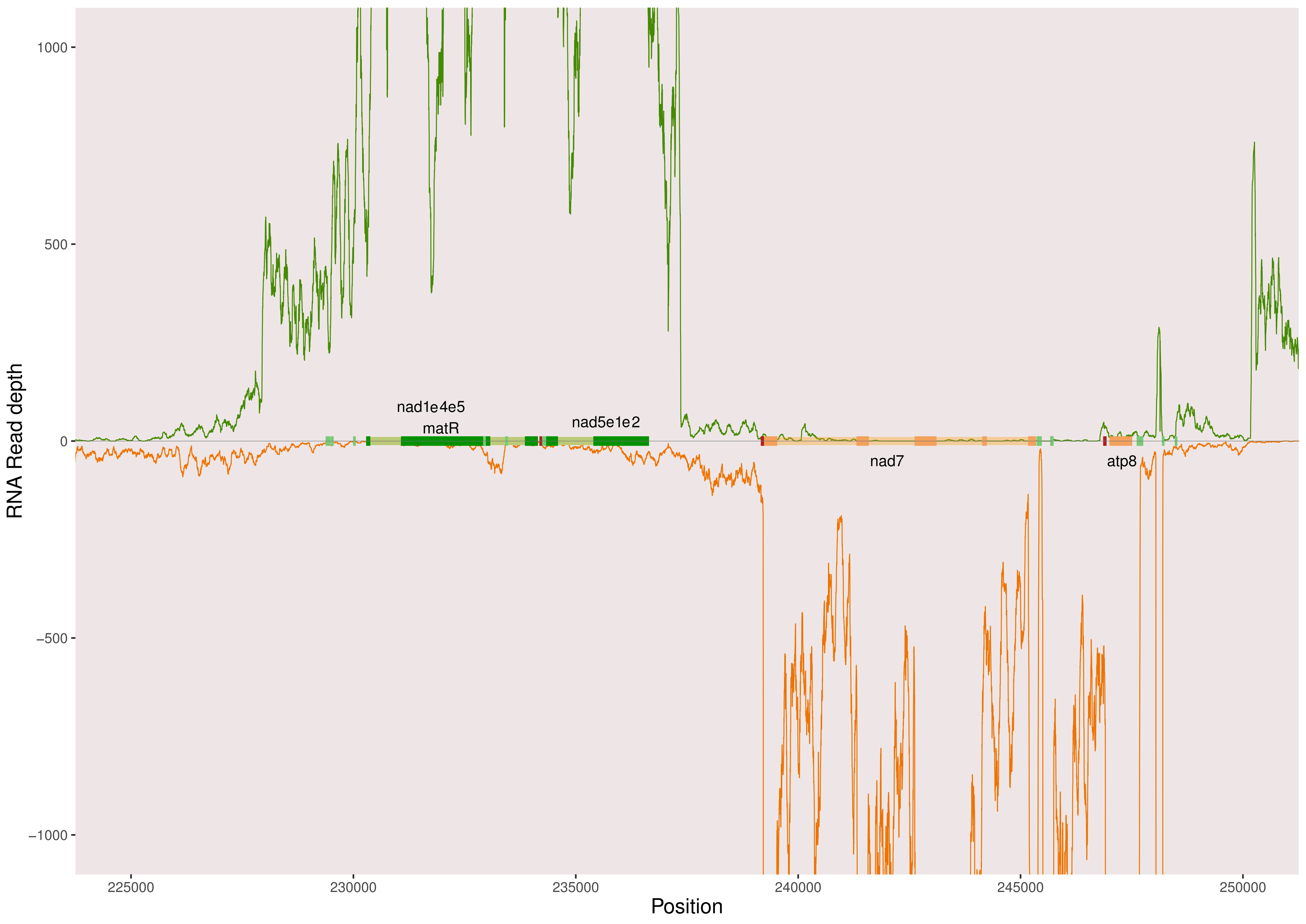

1000
500
0
−500
−1000
RNA Read depth
nad1e4e5
matR
nad5e1e2
nad7
atp8
225000
230000
235000
240000
245000
250000
Position

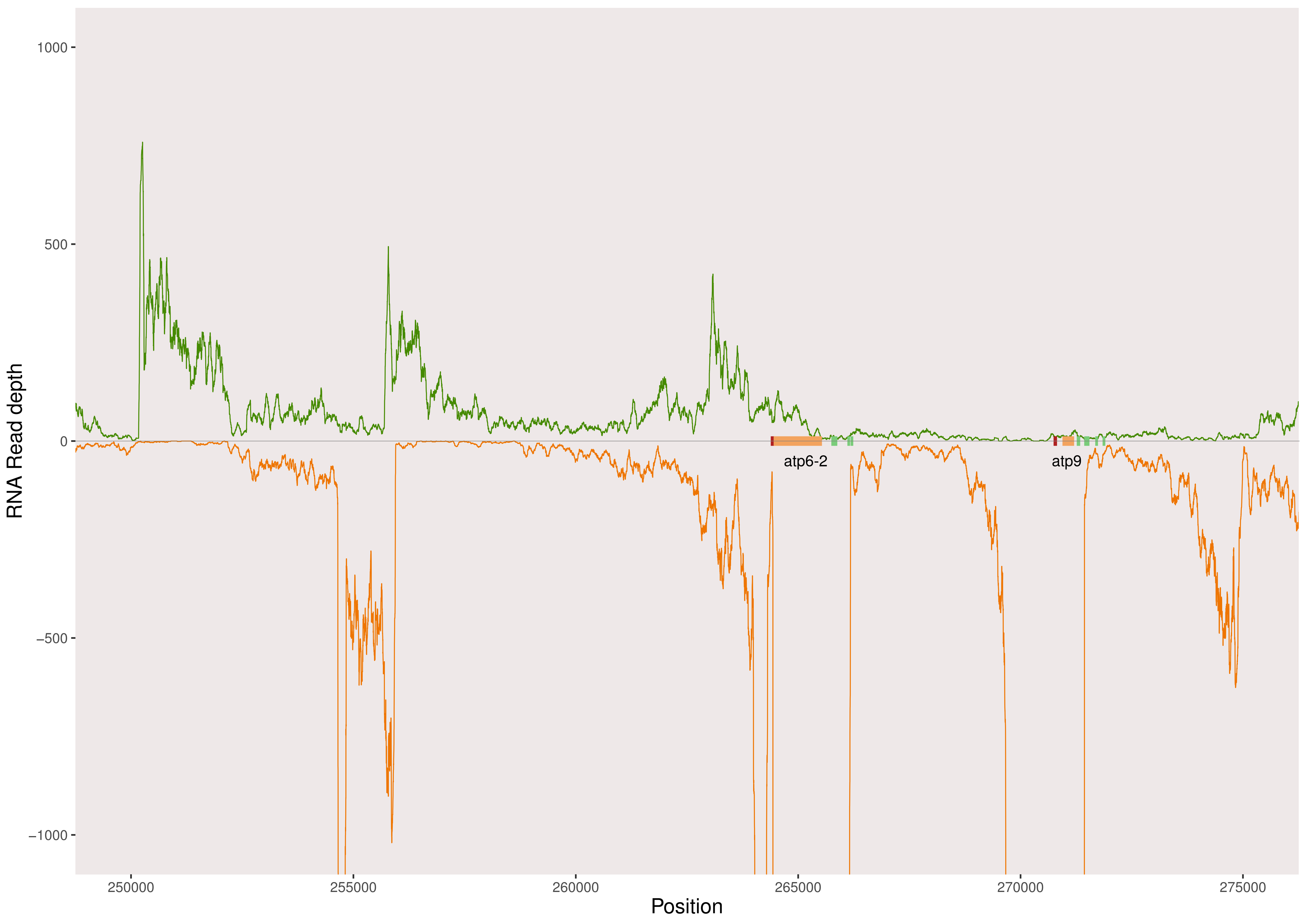

1000
500
0
−500
−1000
RNA Read depth
atp6-2
atp9
250000
255000
260000
265000
270000
275000
Position

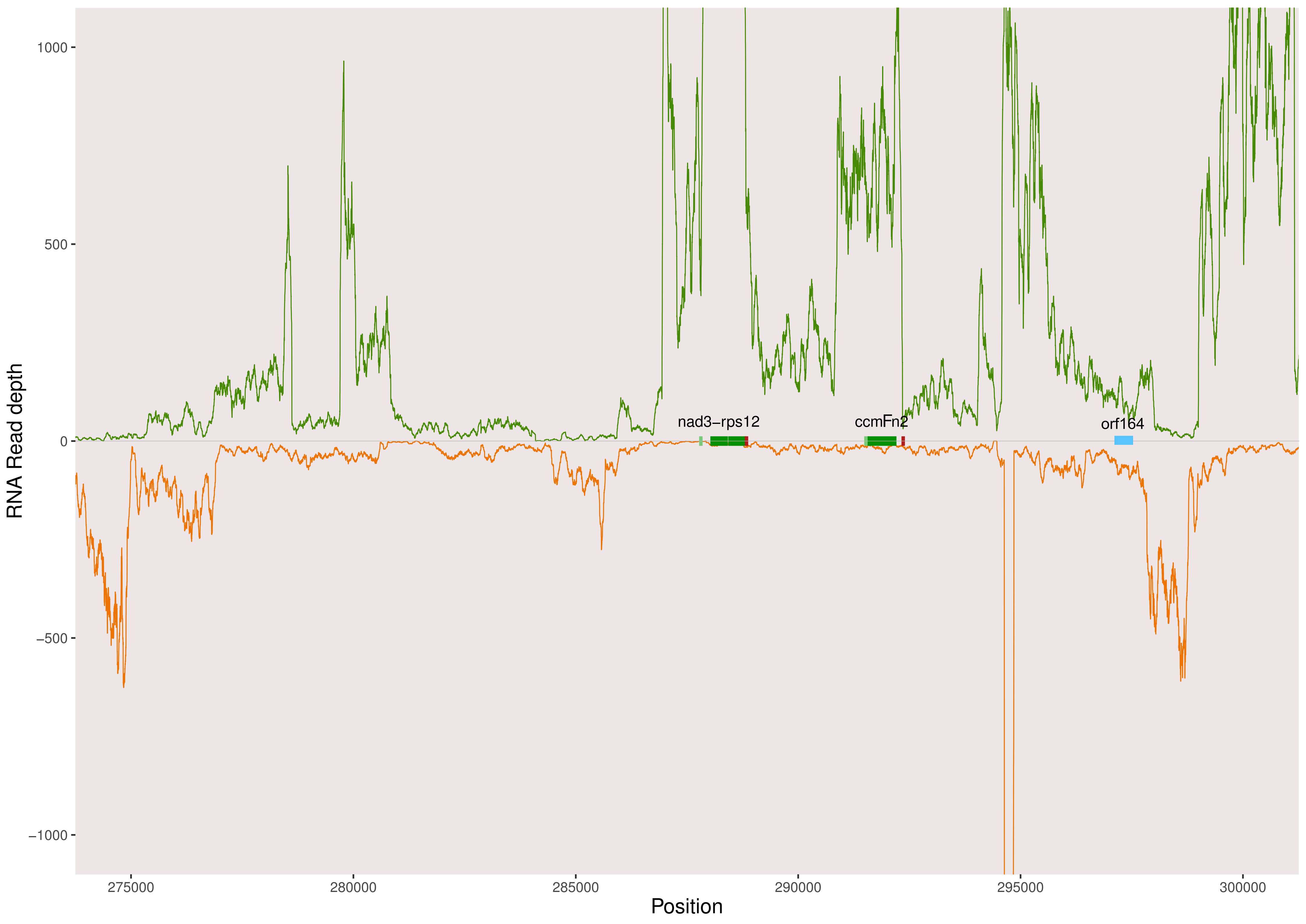

RNA Read depth
1000
500
0
−500
−1000
nad3−rps12
ccmFn2
orf164
275000
280000
285000
290000
295000
300000
Position

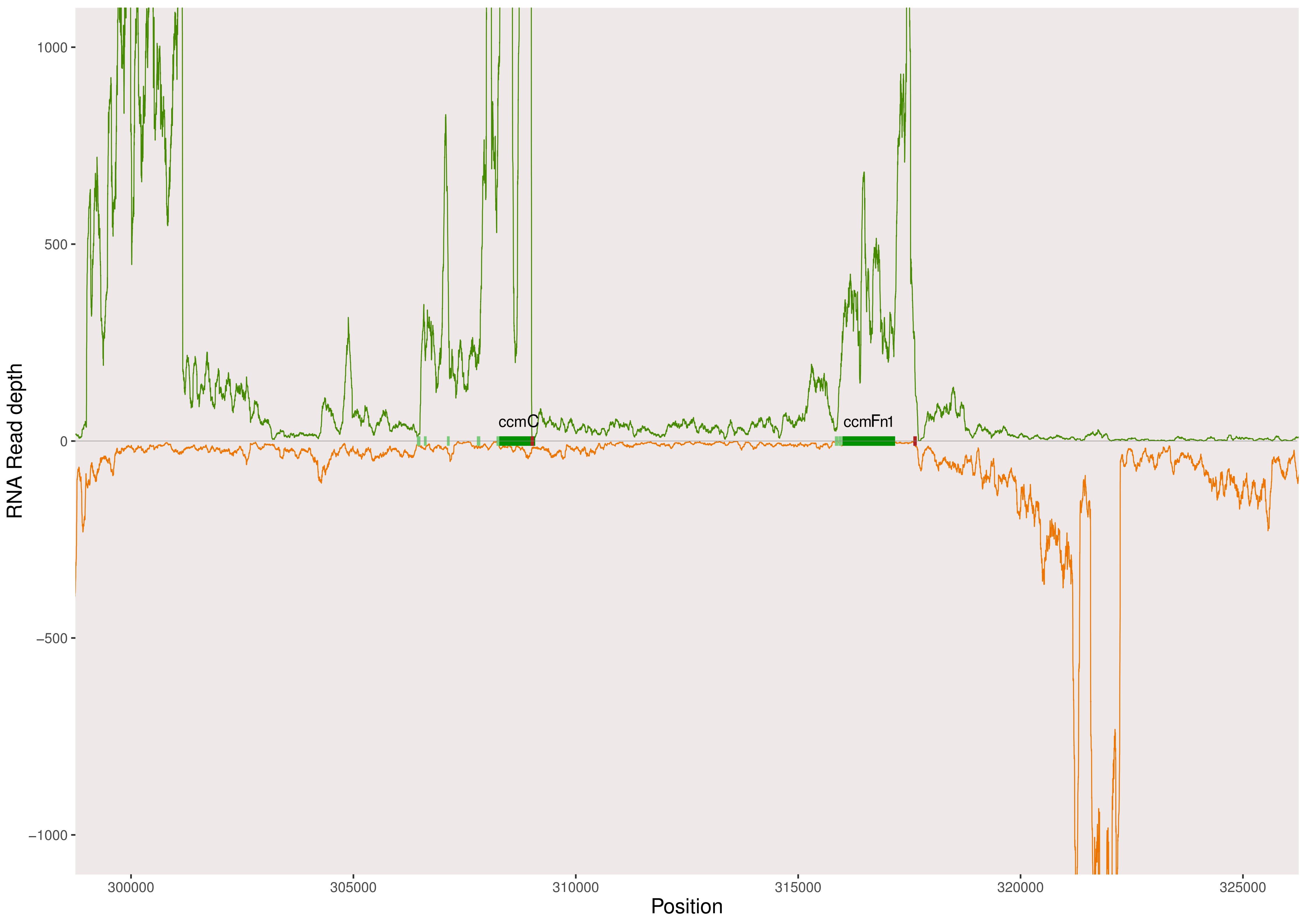

RNA Read depth
Position
1000
500
0
−500
−1000
300000
305000
310000
315000
320000
325000
ccmC
ccmFn1

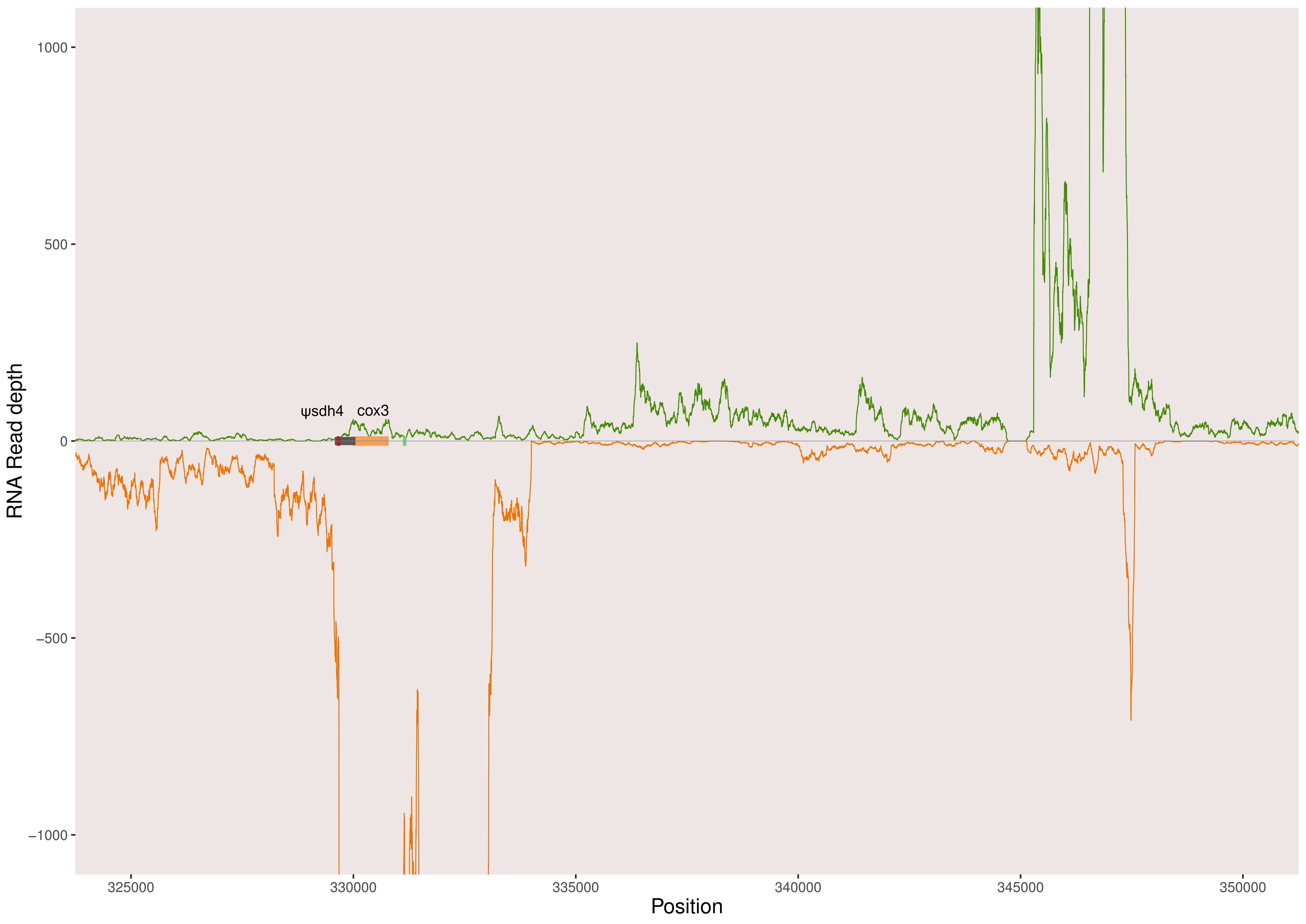

ψsdh4
cox3
RNA Read depth
1000
500
0
−500
−1000
325000
330000
335000
340000
345000
350000
Position

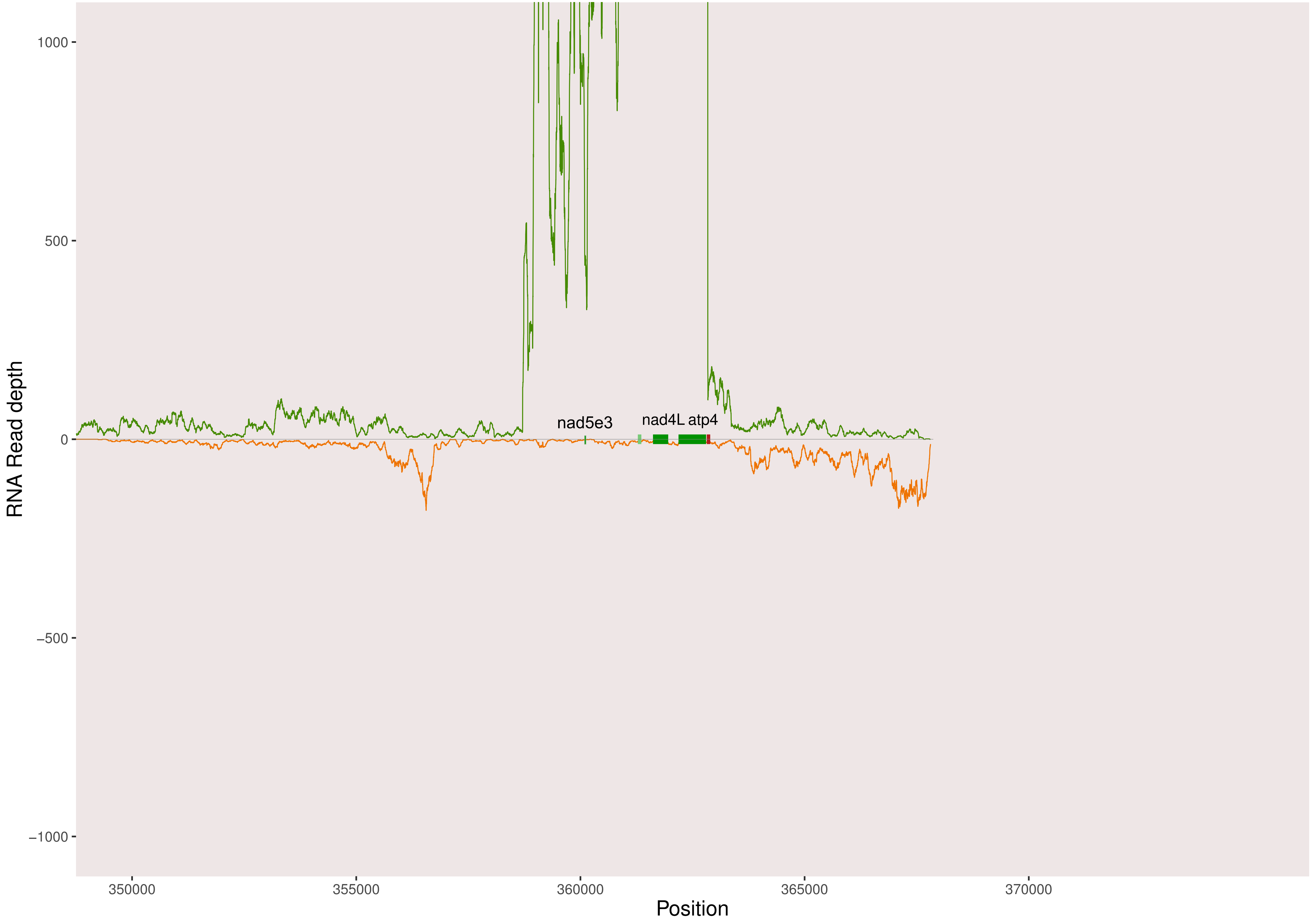

RNA Read depth
1000
500
0
−500
−1000
nad5e3
nad4L
atp4
350000
355000
360000
365000
370000
Position
