# Supplementary material for: Transcriptional Landscape and Splicing Efficiency in Arabidopsis Mitochondria
Source: Cells. 2021 Aug 11;10(8):2054. doi: 10.3390/cells10082054 (PMC8392254; doi:10.3390/cells10082054)

Fig.S2. The frequency distribution of RNAseq read depth calculated based on 100-bp windows across both strands of the entire *Arabidopsis* mitochondrial genome.

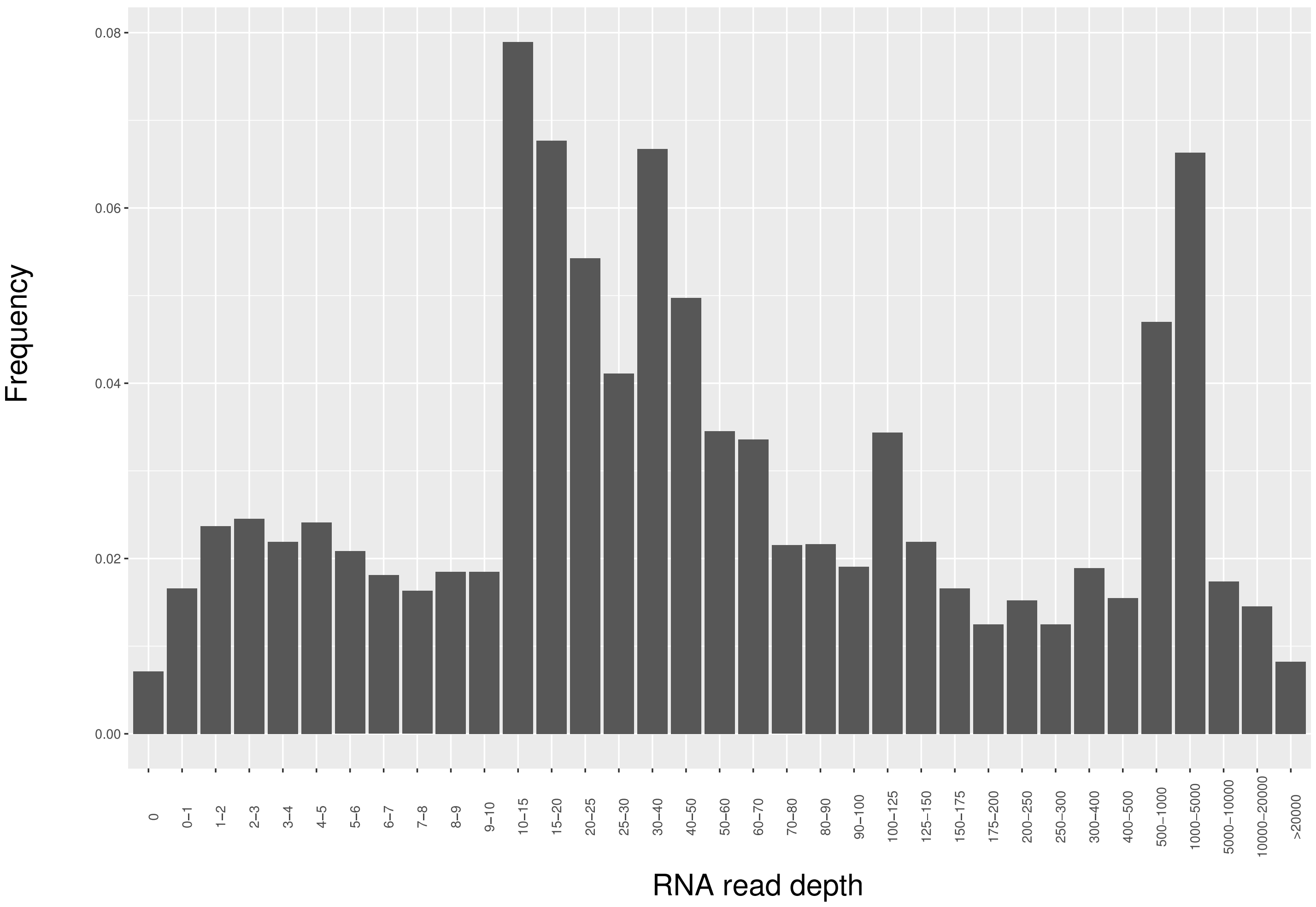

Supplement: Supplementary file 1 [file cells-10-02054-s001.zip › FigS2.pdf]
