# Supplementary material for: Transcriptional Landscape and Splicing Efficiency in Arabidopsis Mitochondria
Source: Cells. 2021 Aug 11;10(8):2054. doi: 10.3390/cells10082054 (PMC8392254; doi:10.3390/cells10082054)
Supplement: Supplementary file 1 [file cells-10-02054-s001.zip › FigS3.pdf]

Fig. S3. RNA read depth of the protein-coding genes in the *Arabidopsis* mtDNA based on the combined data sets of the three biological samples. The curves plot the number of RNA reads mapped to each nucleotide position. Shown are the coding sequences of each gene (gray rectangles) and 100 bp flanking regions. For intron-containing genes, the spliced and unspliced versions of the gene are shown. The x-axis shows the coordinates of the gene.

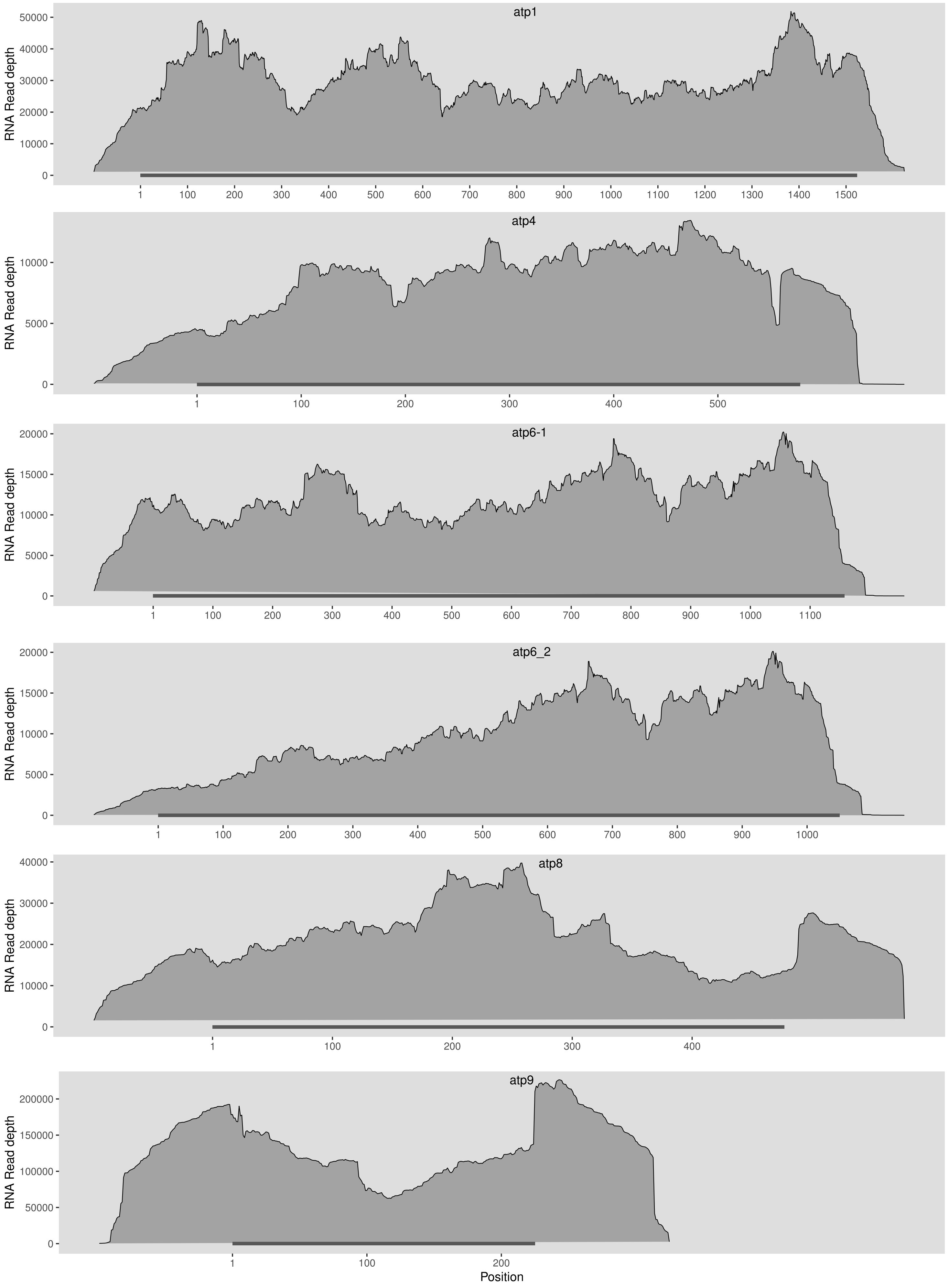

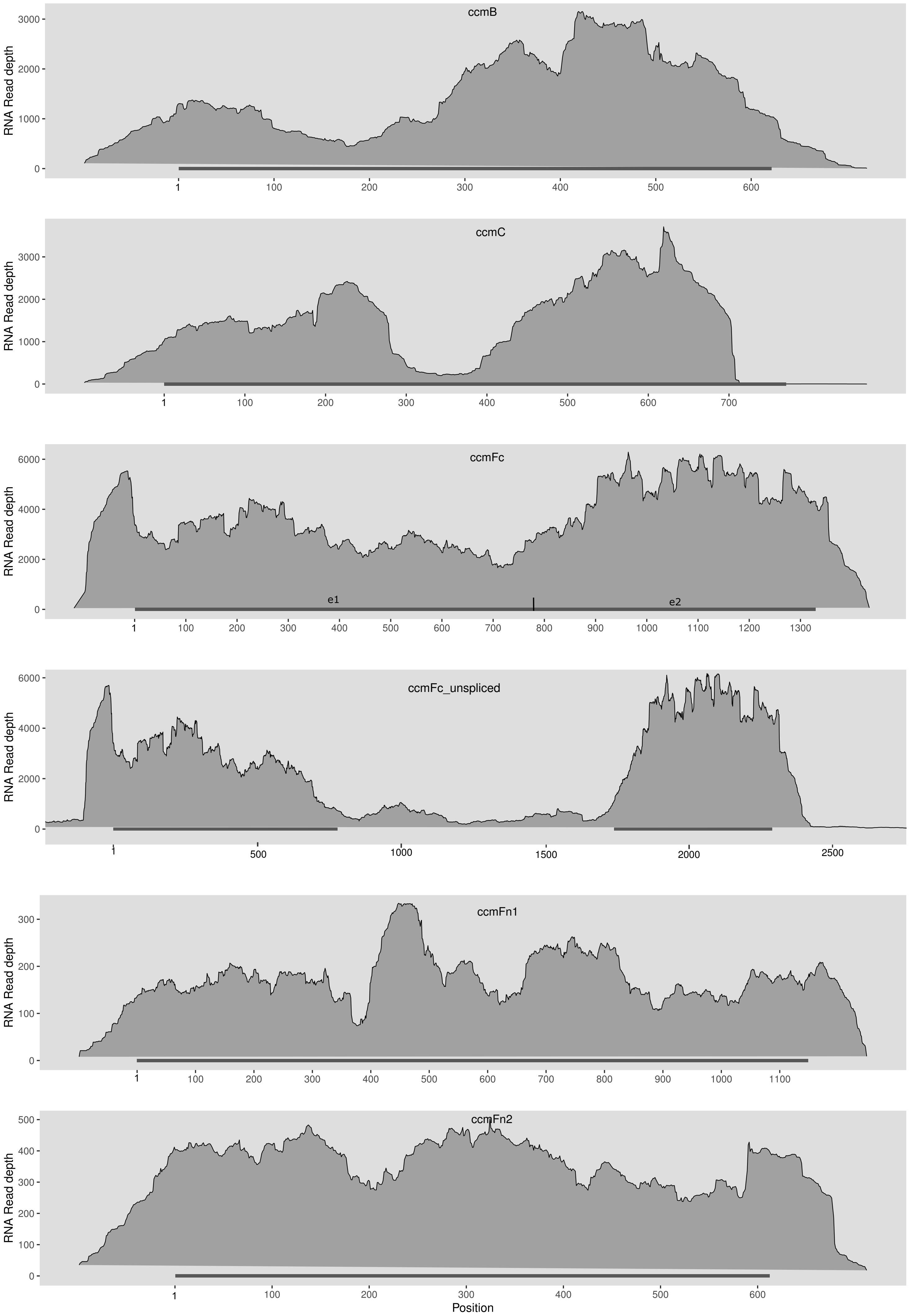

ccmB
ccmC
ccmFc
e1
e2
ccmFc_unspliced
ccmFn1
ccmFn2
RNA Read depth
Position

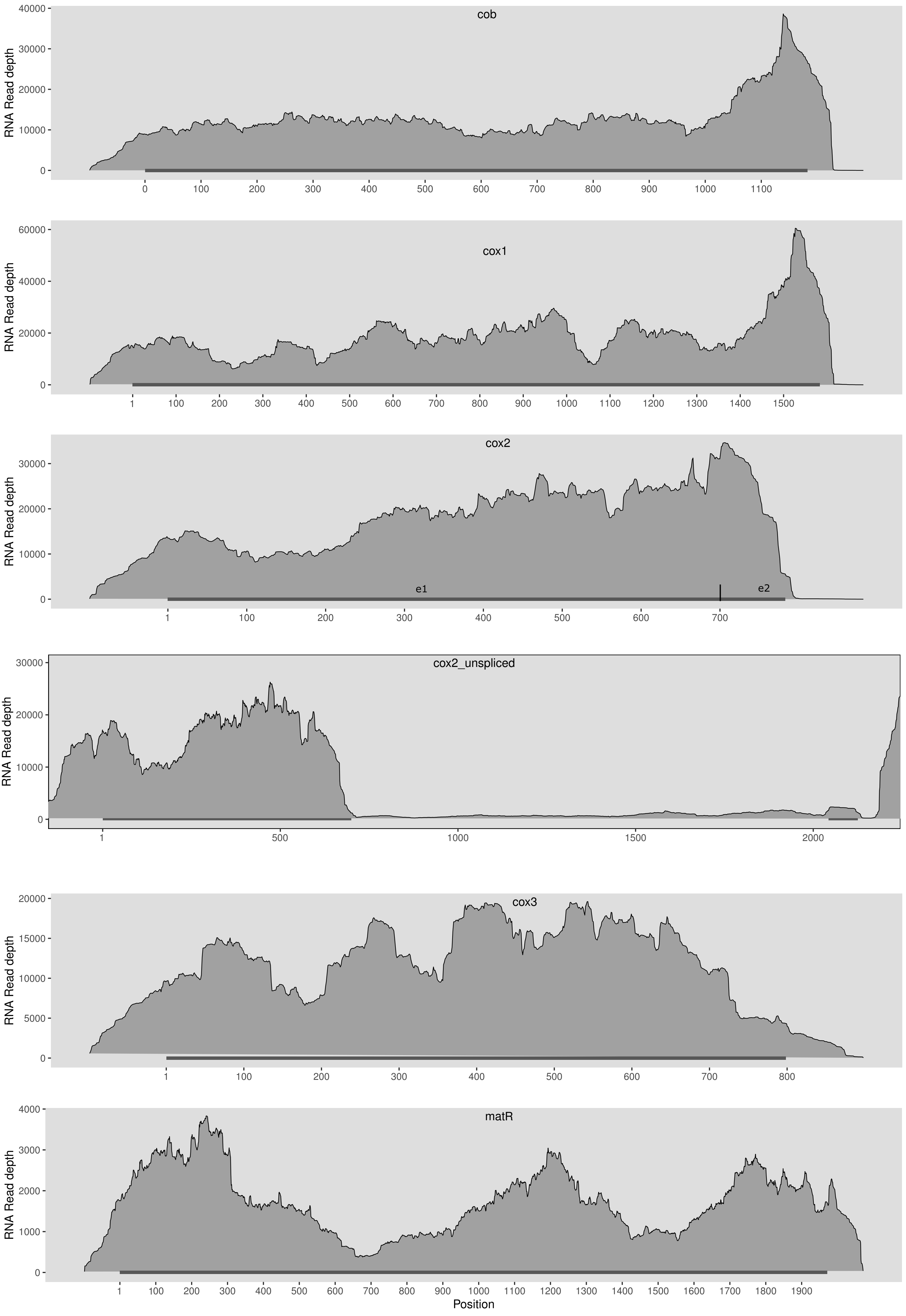
cob
cox1
cox2
e1
e2
cox2_unspliced
cox3
matR
RNA Read depth
Position
0
10000
20000
30000
40000
60000
5000
15000
1000
2000
3000
4000
100
200
300
400
500
600
700
800
900
1000
1100
1200
1300
1400
1500
1600
1700
1800
1900
2000
1

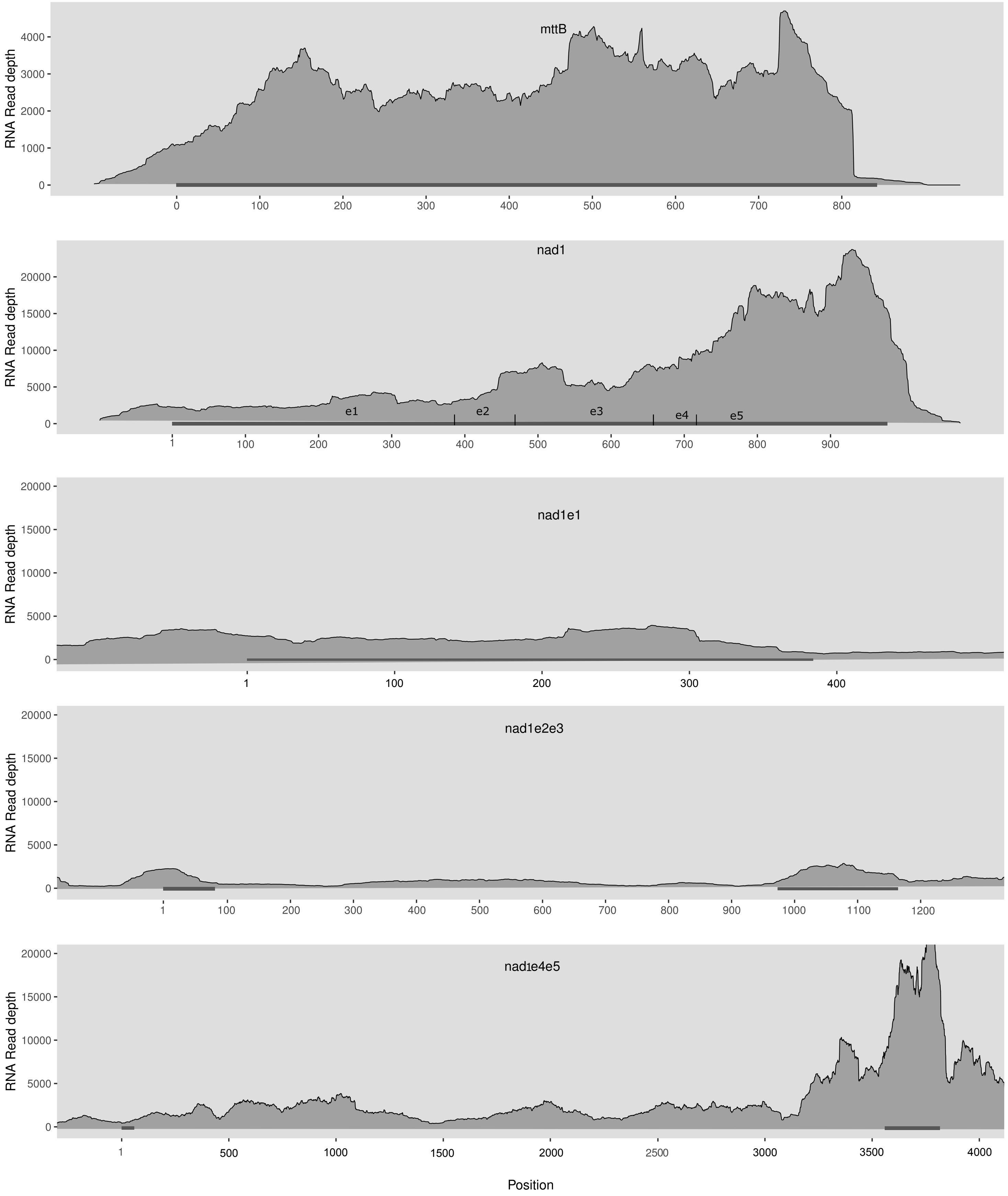
mttB
nad1
e1
e2
e3
e4
e5
nad1e1
nad1e2e3
nad1e4e5
RNA Read depth
Position

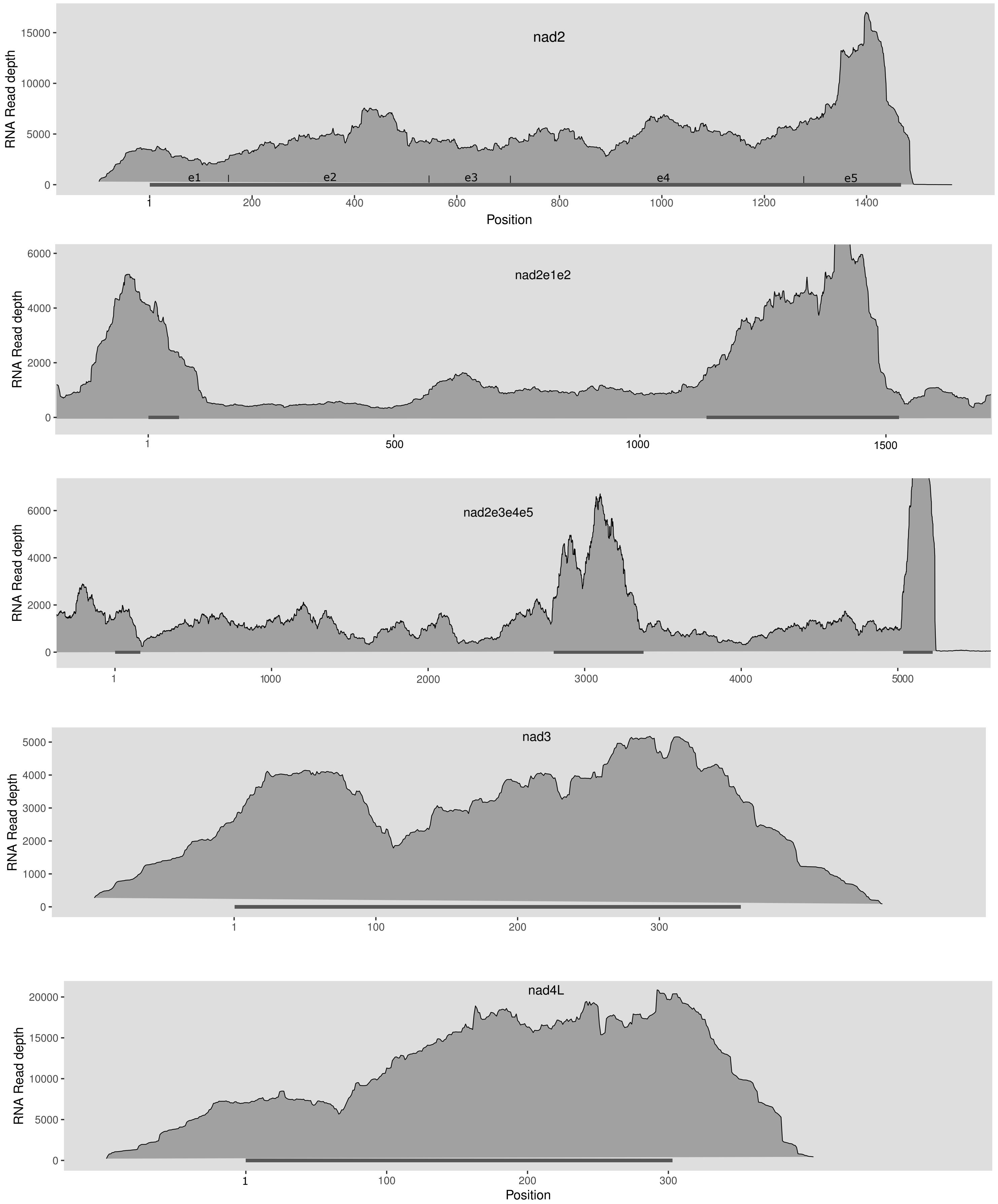
nad2
RNA Read depth
0
5000
10000
15000
e1
e2
e3
e4
e5
1
200
400
600
800
1000
1200
1400
Position
nad2e1e2
RNA Read depth
0
2000
4000
6000
1
500
1000
1500
nad2e3e4e5
RNA Read depth
0
2000
4000
6000
1
1000
2000
3000
4000
5000
nad3
RNA Read depth
0
1000
2000
3000
4000
5000
1
100
200
300
nad4L
RNA Read depth
0
5000
10000
15000
20000
1
100
200
300
Position

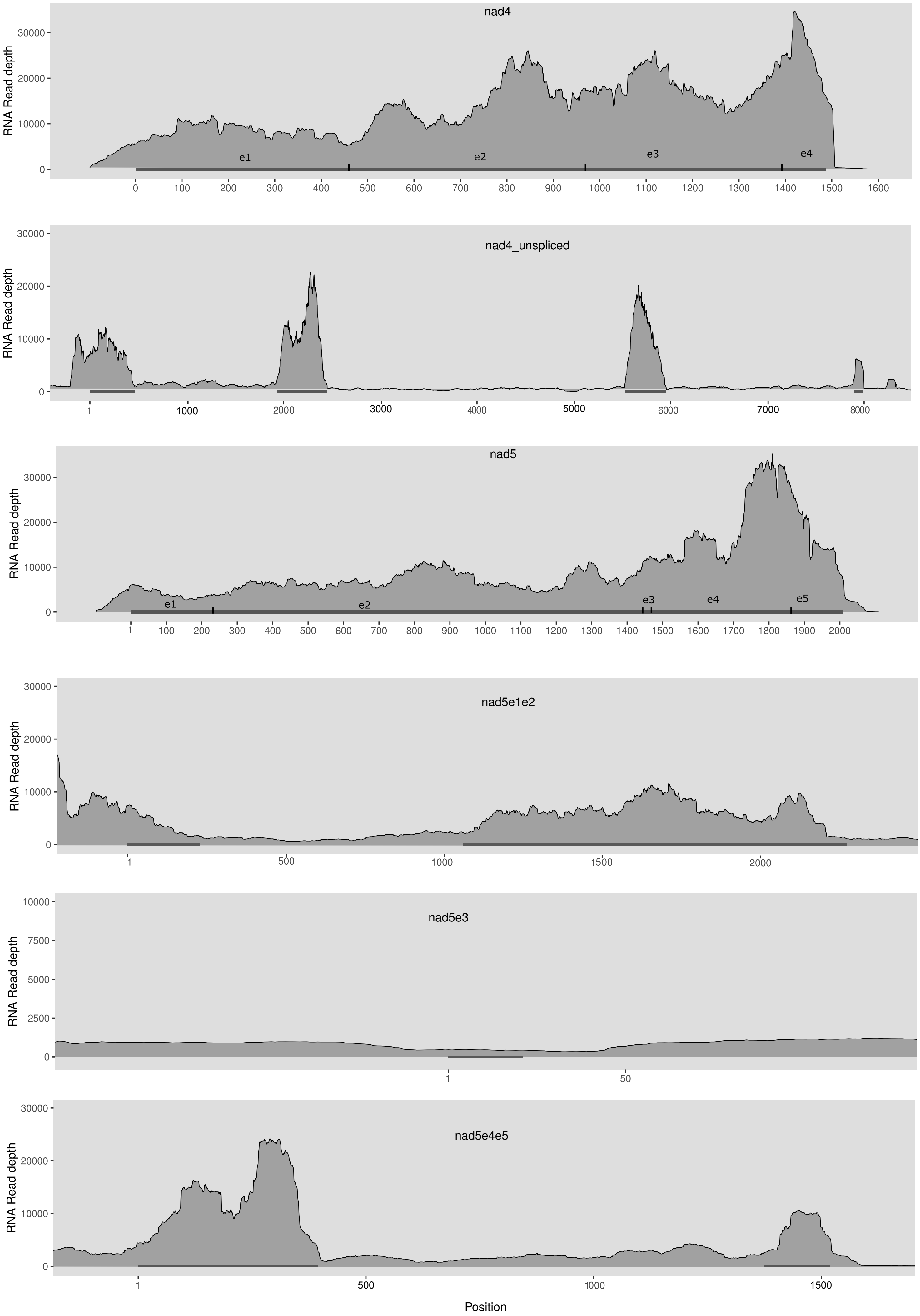

nad4
RNA Read depth
0
10000
20000
30000
e1
e2
e3
e4
0
100
200
300
400
500
600
700
800
900
1000
1100
1200
1300
1400
1500
1600
nad4_unspliced
RNA Read depth
0
10000
20000
30000
1
1000
2000
3000
4000
5000
6000
7000
8000
nad5
RNA Read depth
0
10000
20000
30000
e1
e2
e3
e4
e5
1
100
200
300
400
500
600
700
800
900
1000
1100
1200
1300
1400
1500
1600
1700
1800
1900
2000
nad5e1e2
RNA Read depth
0
10000
20000
30000
1
500
1000
1500
2000
nad5e3
RNA Read depth
0
2500
5000
7500
10000
1
50
nad5e4e5
RNA Read depth
0
10000
20000
30000
1
500
1000
1500
Position

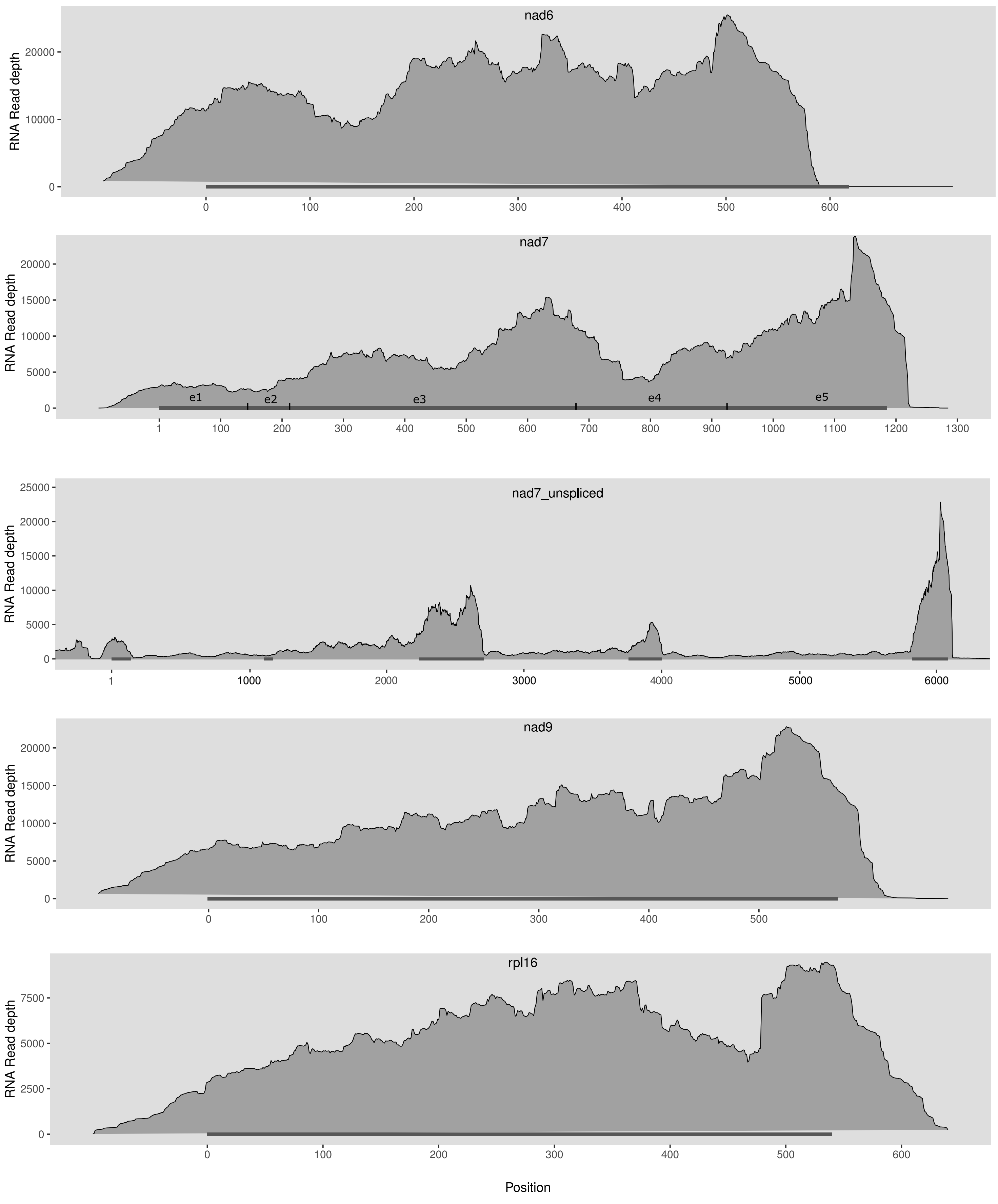

nad6
nad7
e1
e2
e3
e4
e5
nad7_unspliced
nad9
rpl16
RNA Read depth
Position

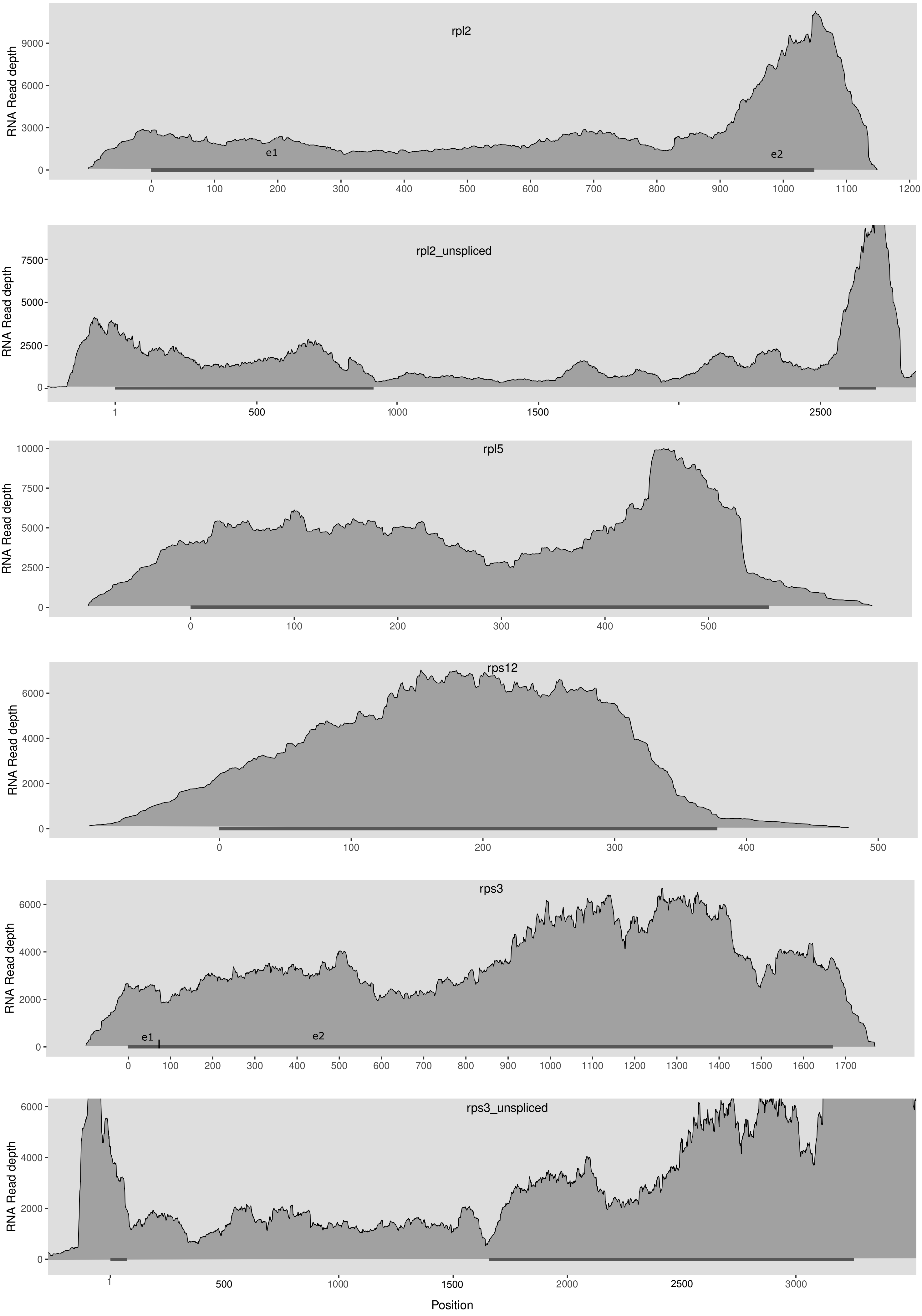

rpl2
e1
e2
RNA Read depth
0
3000
6000
9000
0 100 200 300 400 500 600 700 800 900 1000 1100 1200
rpl2_unspliced
0
2500
5000
7500
1 500 1000 1500 2500
rpl5
0
2500
5000
7500
10000
0 100 200 300 400 500
rps12
0
2000
4000
6000
0 100 200 300 400 500
rps3
e1
e2
0
2000
4000
6000
0 100 200 300 400 500 600 700 800 900 1000 1100 1200 1300 1400 1500 1600 1700
rps3_unspliced
0
2000
4000
6000
1 500 1000 1500 2000 2500 3000
Position

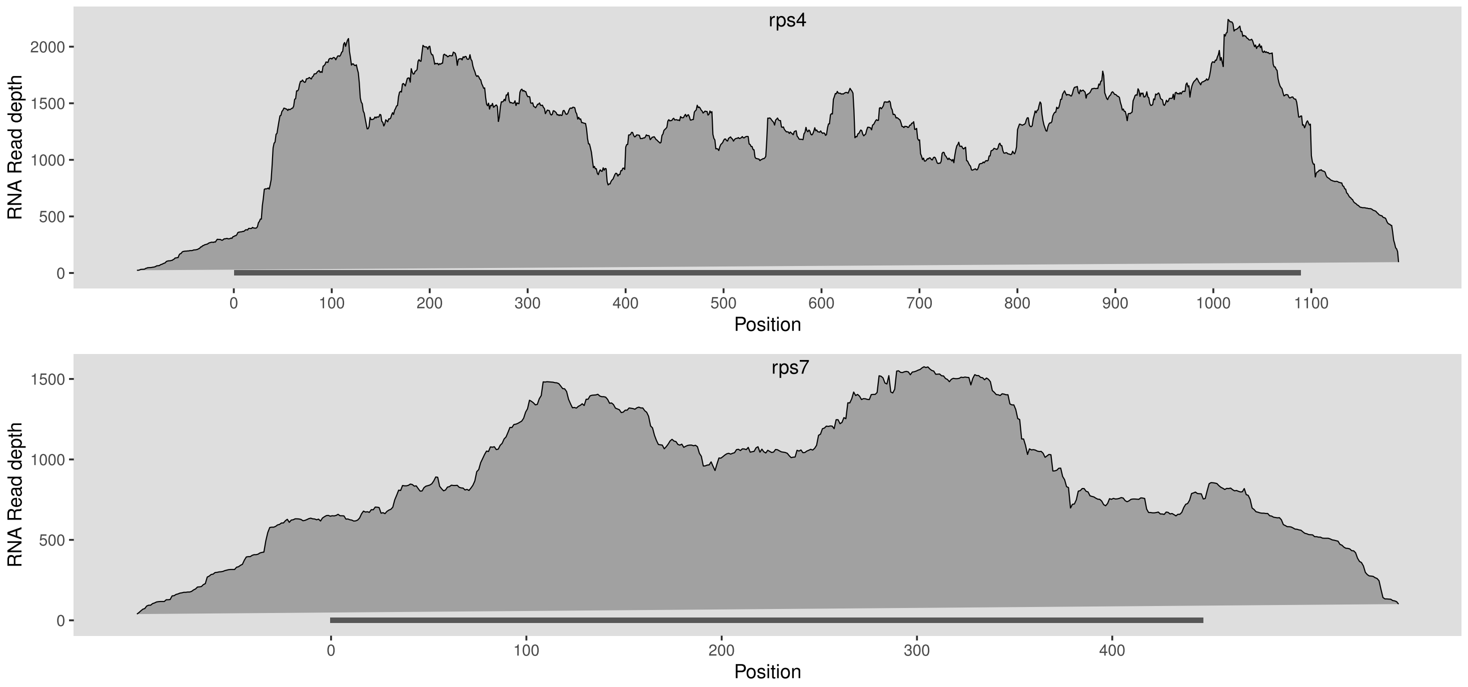
rps4
RNA Read depth
0
500
1000
1500
2000
0
100
200
300
400
500
600
700
800
900
1000
1100
Position
rps7
RNA Read depth
0
500
1000
1500
0
100
200
300
400
Position
