# Supplementary material for: Transcriptional Landscape and Splicing Efficiency in Arabidopsis Mitochondria
Source: Cells. 2021 Aug 11;10(8):2054. doi: 10.3390/cells10082054 (PMC8392254; doi:10.3390/cells10082054)
Supplement: Supplementary file 1 [file cells-10-02054-s001.zip › FigS4.pdf]

Fig. S4. RNA read depth of protein-coding genes and 2 kb flanking regions in the *Arabidopsis* mtDNA based on the combined data sets of the three biological samples. Only genes for which promoters have been experimentally identified (Table S2) are included. The slopes of the read depth (filled circles) were calculated every 50 bp in the 2 kb region upstream of each gene or coding units. Gray bars represent genes and dark gray bars are exons. Green and red squares represent start of transcription and 3' termini, respectively, which were identified experimentally [36,42,44]. Blue squares indicate sequences identical to reported promoters for other genes. The higher values of the slopes that fall within a 100 bp window around the transcription start sites identified experimentally are shown as green circles. The higher values of the slopes found upstream of the transcription start sites identified experimentally are shown as blue circles. Below each graph, the mtDNA coordinates of the strand in which the gene is located are shown. For each intron-containing gene, the spliced version of the gene is also shown, and the coordinates of the spliced reference sequence are depicted below each graph.

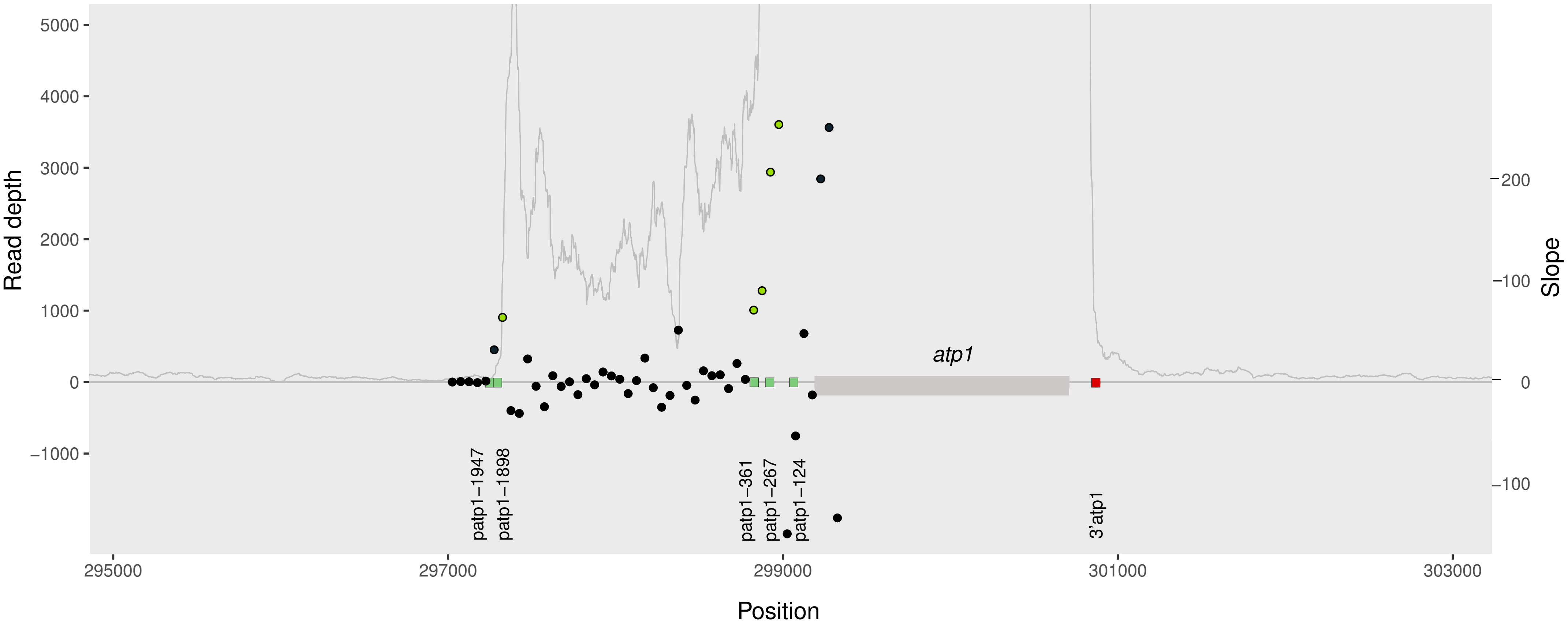

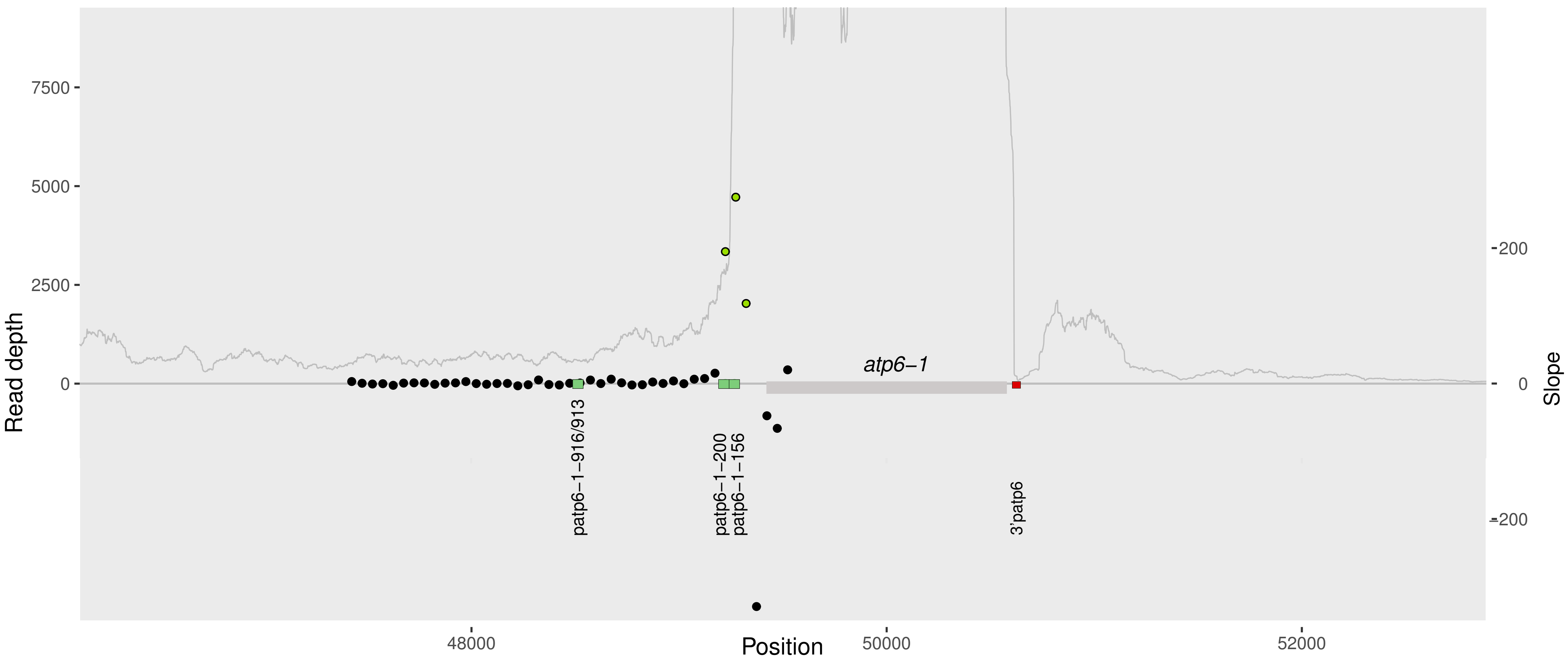

Read depth
7500
5000
2500
0
Slope
200
0
-200
atp6−1
patp6−1−916/913
patp6−1−200
patp6−1−156
3'patp6
48000
50000
52000
Position

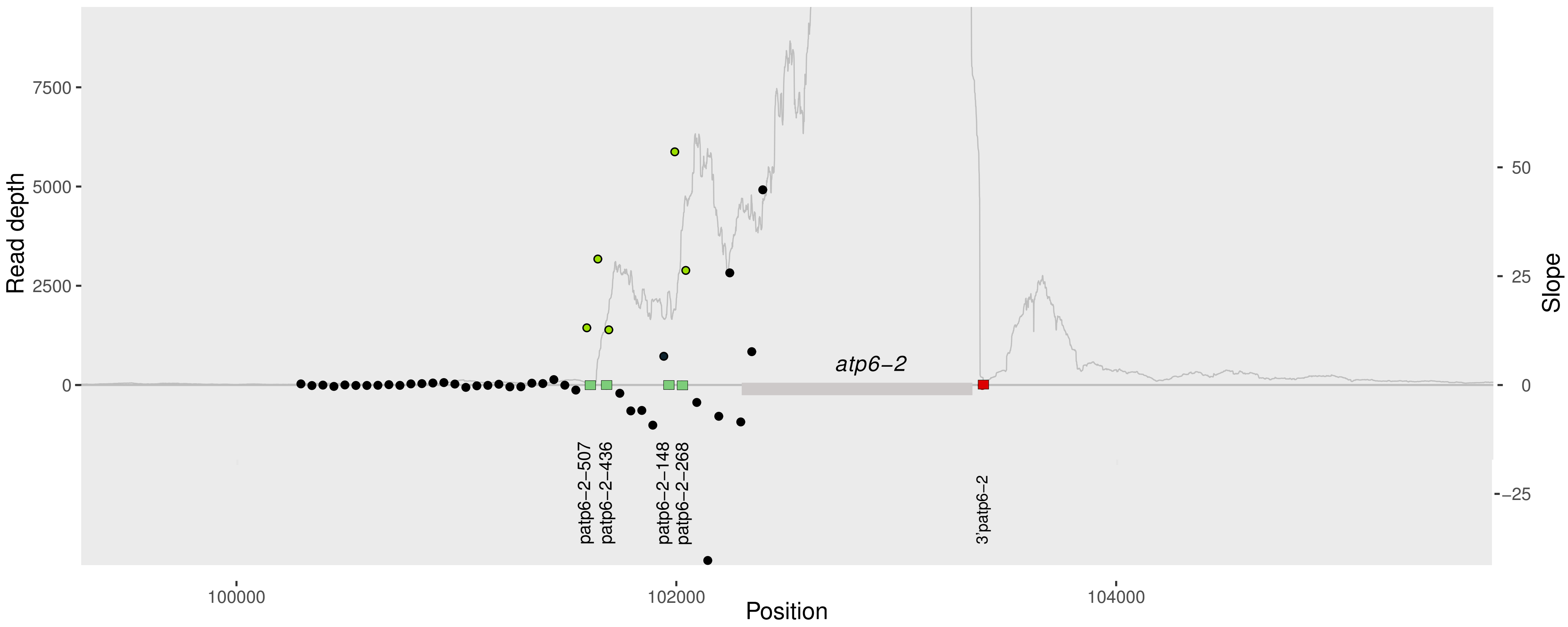

Read depth
7500
5000
2500
0
Slope
50
25
0
−25
atp6−2
patp6−2−507
patp6−2−436
patp6−2−148
patp6−2−268
3'patp6−2
100000
102000
104000
Position

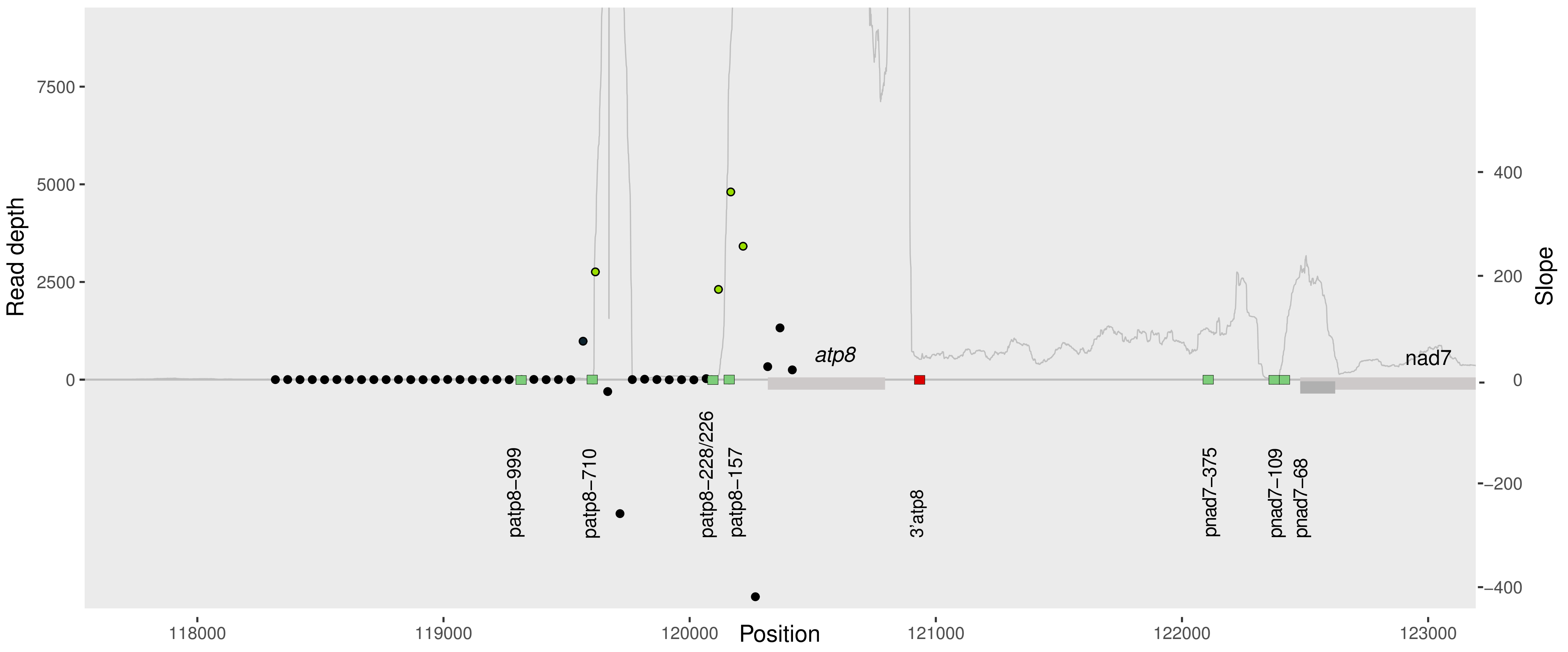
Read depth
Slope
Position
atp8
nad7
patp8−999
patp8−710
patp8−228/226
patp8−157
3'atp8
pnad7−375
pnad7−109
pnad7−68
7500
5000
2500
0
400
200
−200
−400
118000
119000
120000
121000
122000
123000

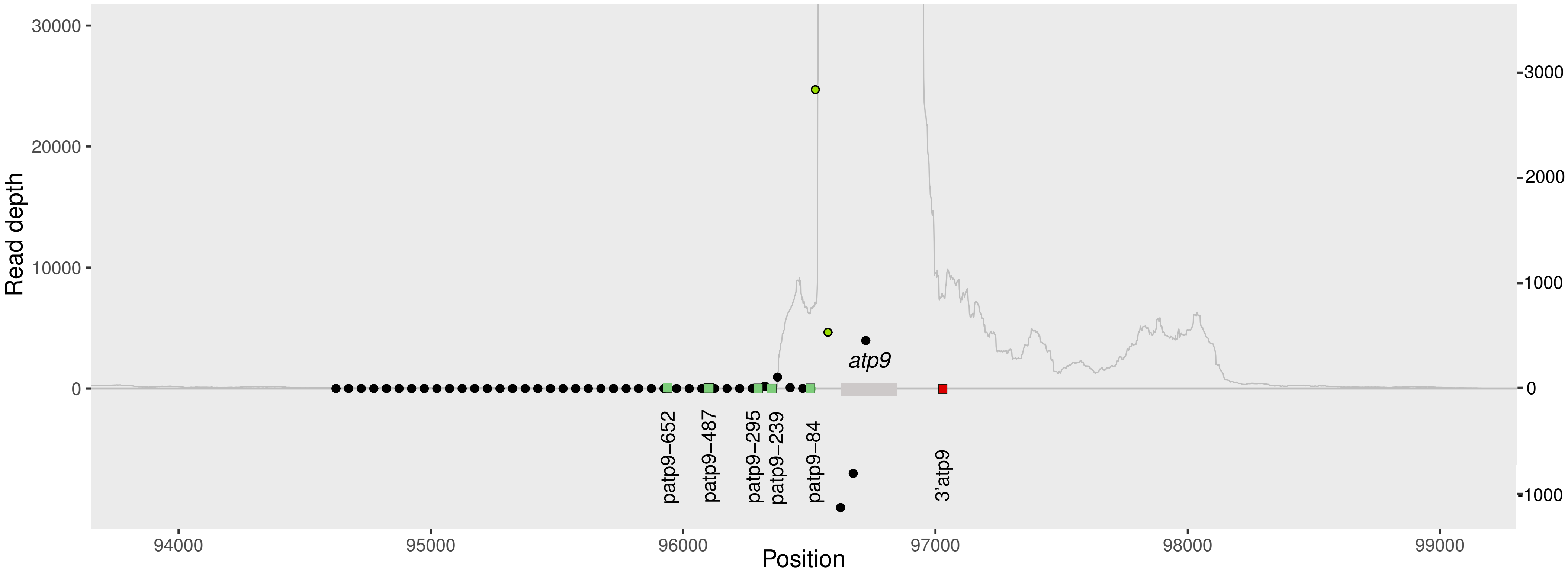

Read depth
30000
20000
10000
0
Slope
3000
2000
1000
0
-1000
atp9
patp9−652
patp9−487
patp9−295
patp9−239
patp9−84
3'atp9
94000
95000
96000
97000
98000
99000
Position

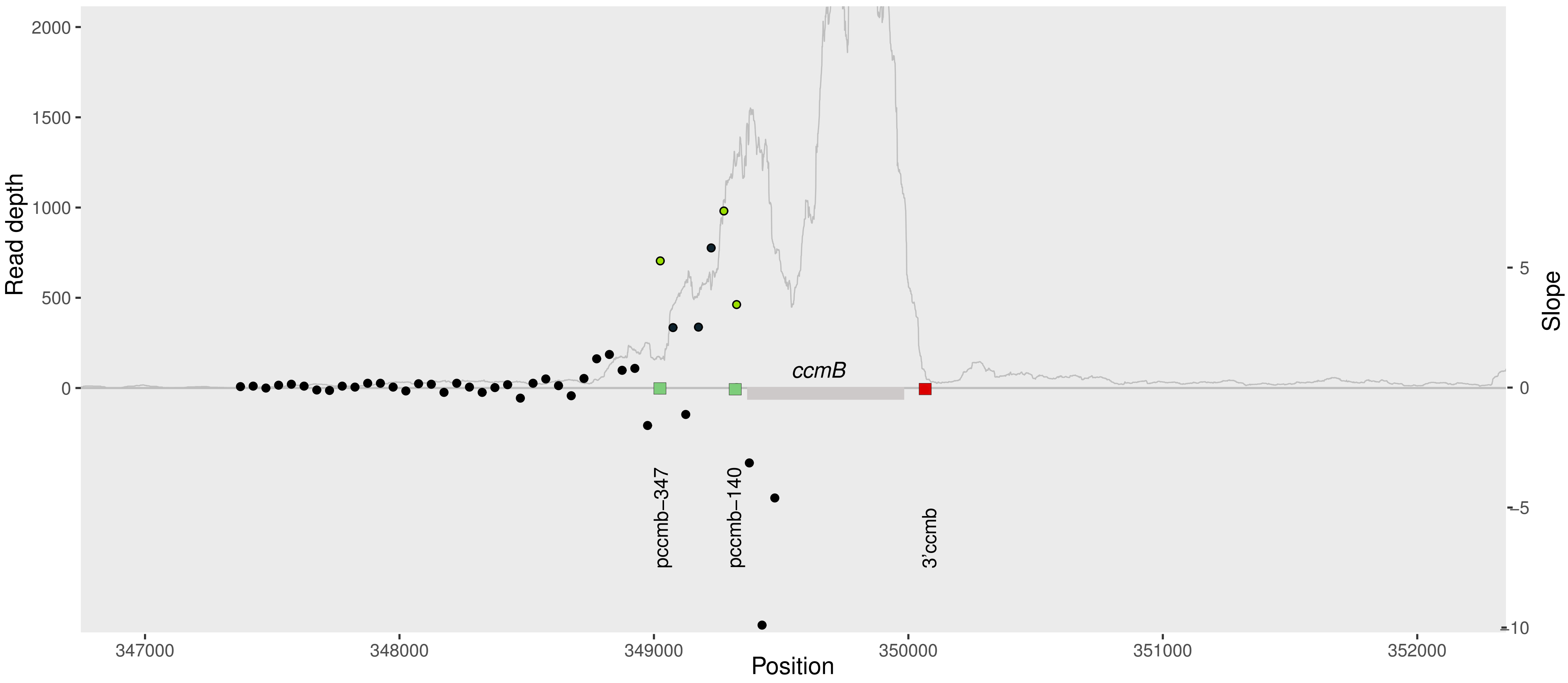

Read depth
2000
1500
1000
500
0
Slope
5
0
−5
−10
ccmB
pccmb−347
pccmb−140
3'ccmb
347000
348000
349000
350000
351000
352000
Position

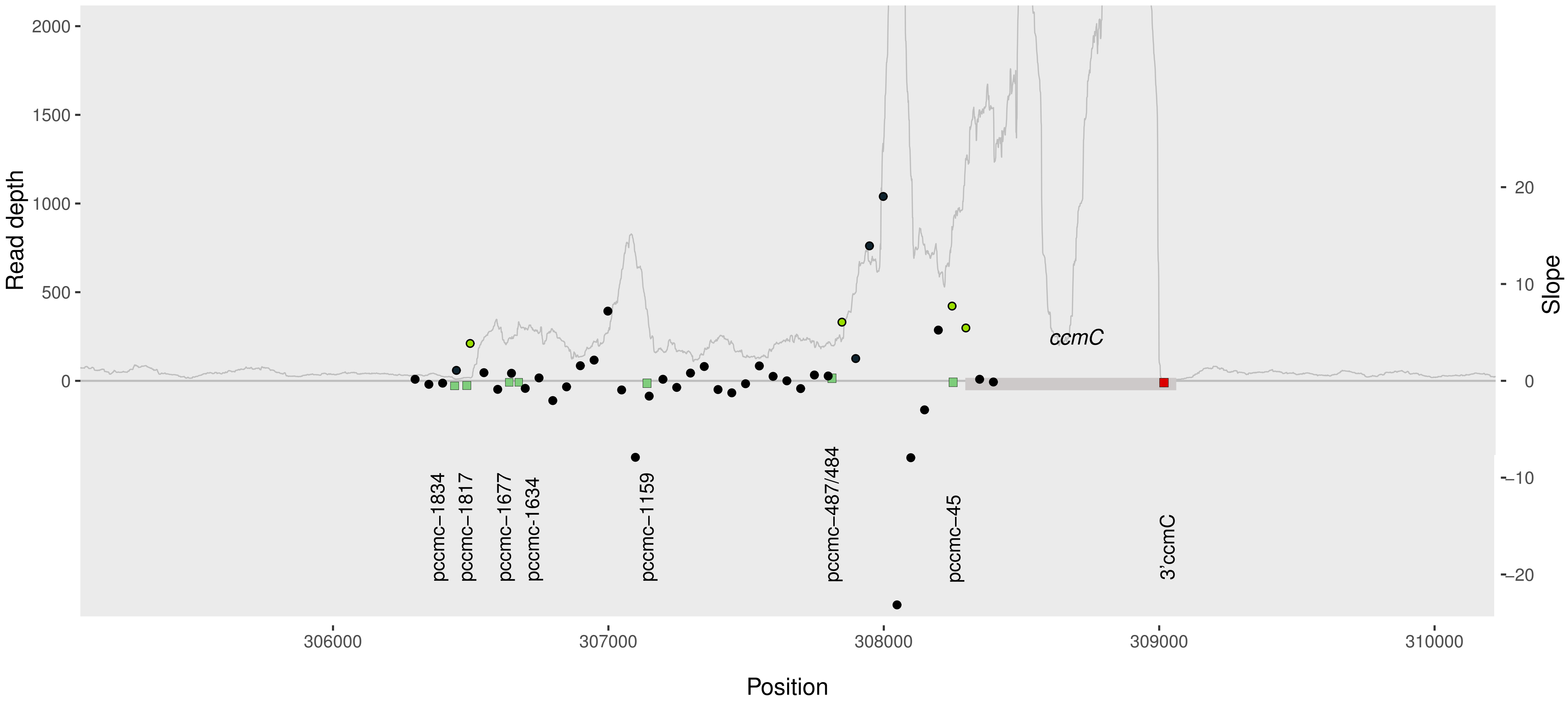

Read depth
2000
1500
1000
500
0
Slope
20
10
0
−10
−20
ccmC
pccmc−1834
pccmc−1817
pccmc−1677
pccmc-1634
pccmc−1159
pccmc−487/484
pccmc−45
3'ccmC
306000
307000
308000
309000
310000
Position

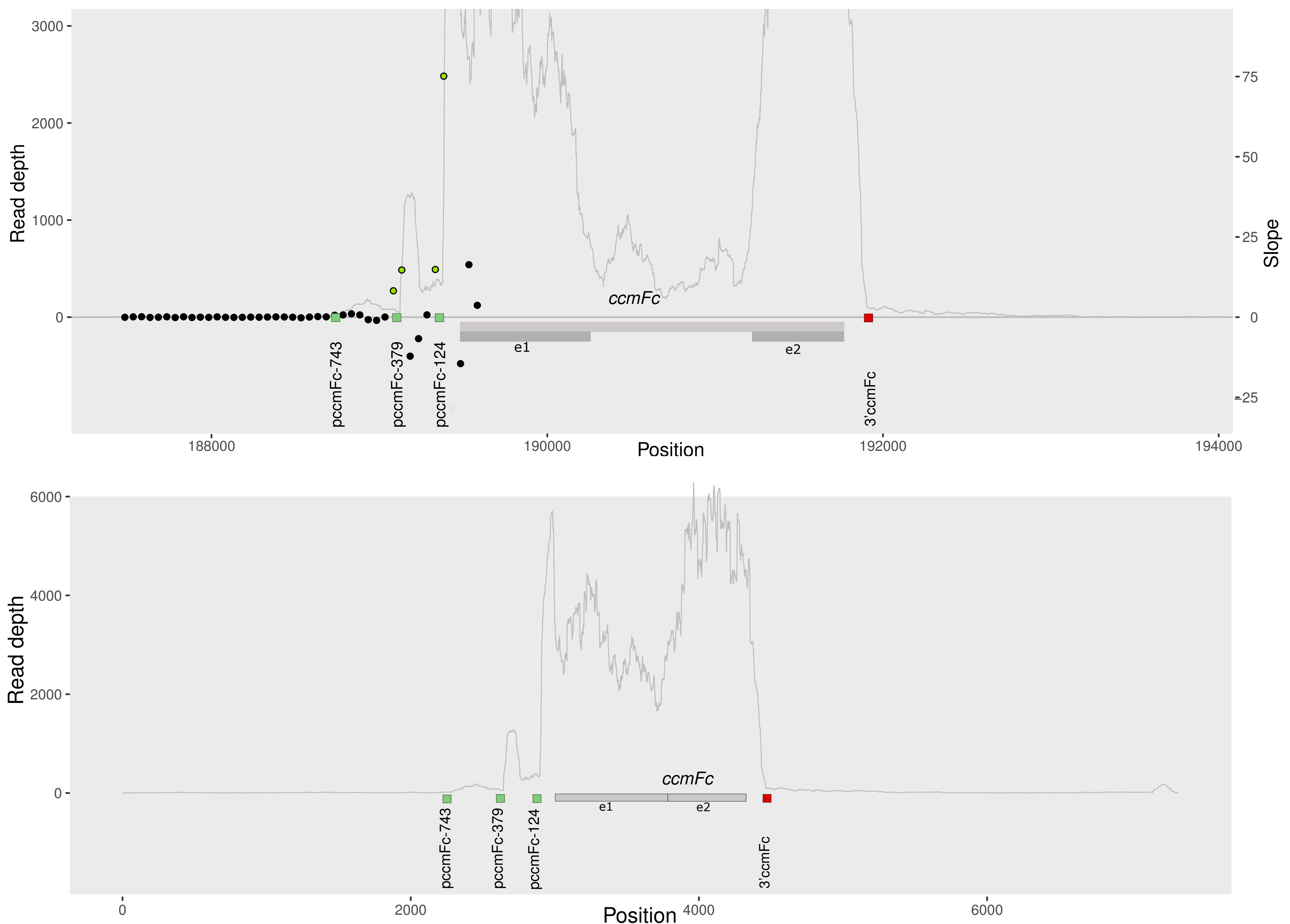

Read depth
3000
2000
1000
0
Slope
75
50
25
0
-25
ccmFc
e1
e2
pccmFc-743
pccmFc-379
pccmFc-124
3'ccmFc
188000
190000
192000
194000
Position
Read depth
6000
4000
2000
0
ccmFc
e1
e2
pccmFc-743
pccmFc-379
pccmFc-124
3'ccmFc
0
2000
4000
6000
Position

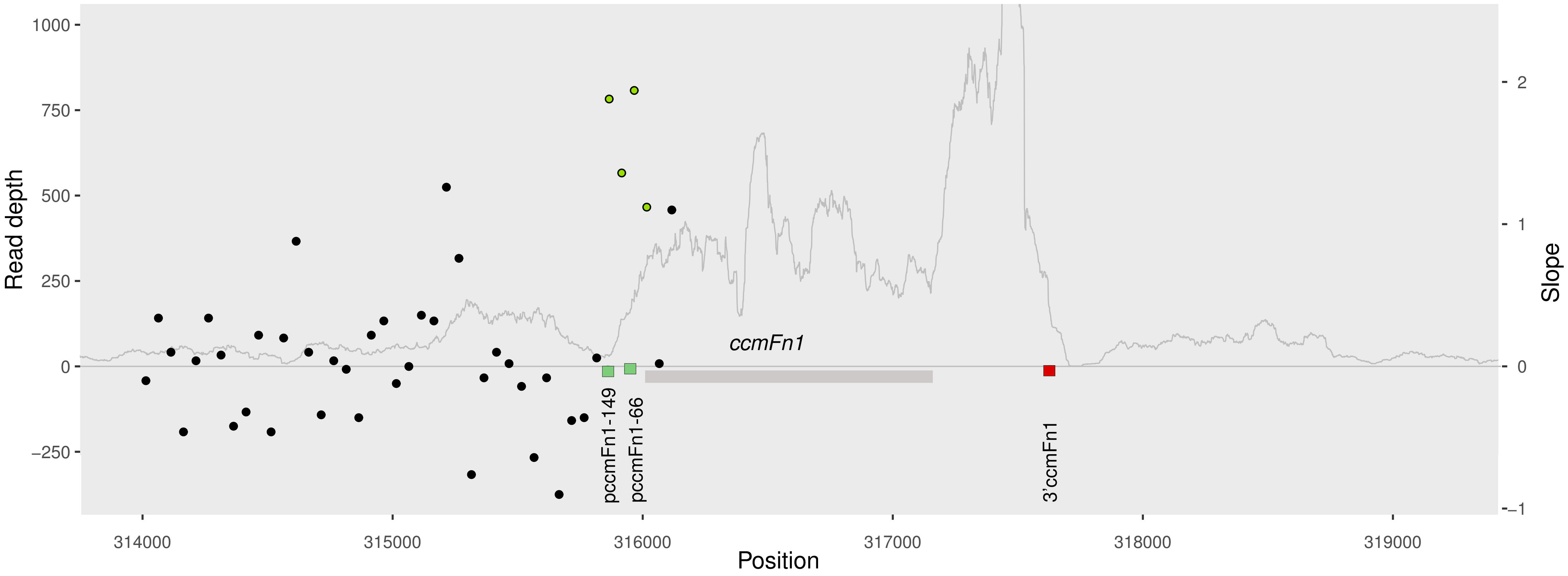

Read depth
Slope
Position
ccmFn1
pccmFn1-149
pccmFn1-66
3'ccmFn1
1000
750
500
250
0
−250
2
1
0
−1
314000
315000
316000
317000
318000
319000

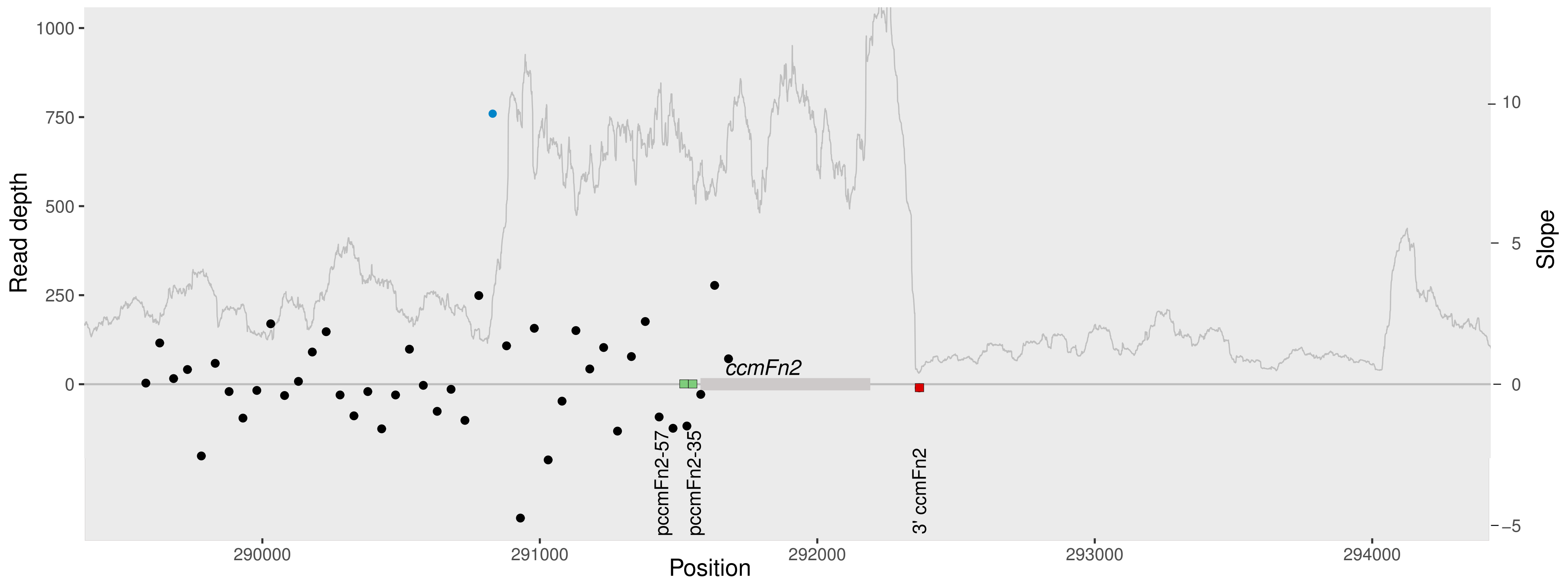

Read depth
Slope
Position
ccmFn2
pccmFn2-57
pccmFn2-35
3' ccmFn2
1000
750
500
250
0
10
5
0
−5
290000
291000
292000
293000
294000

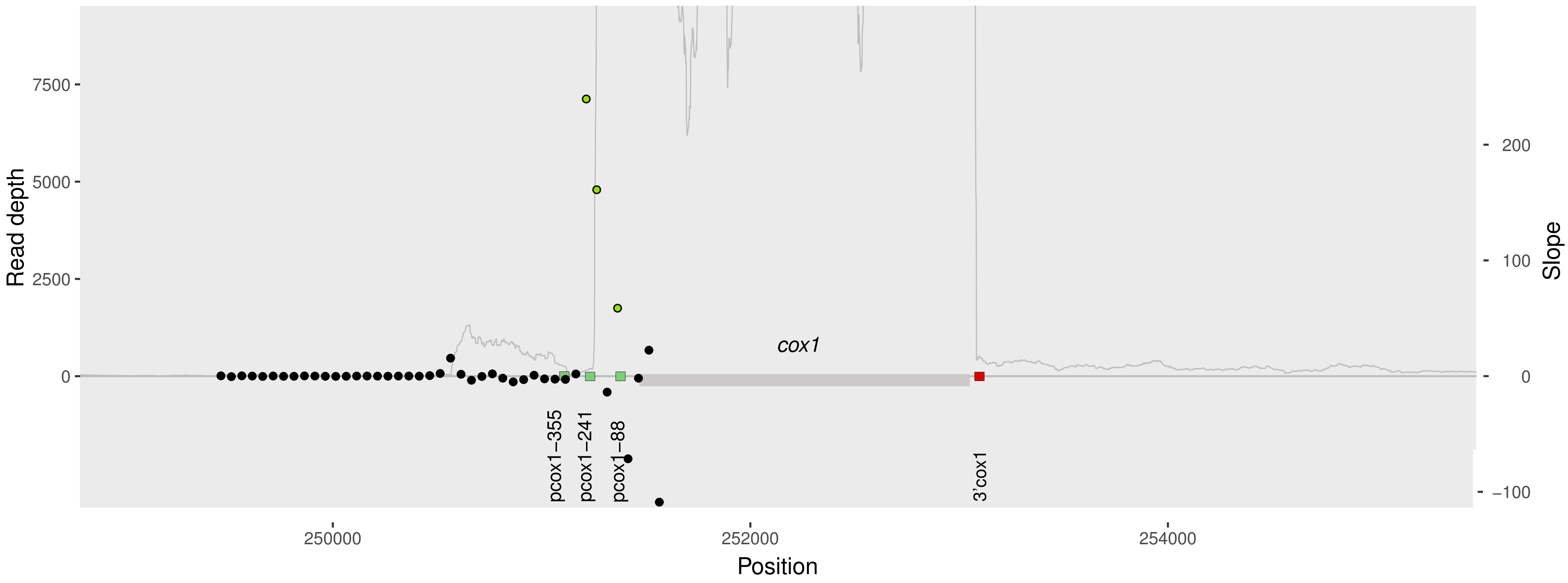

Read depth
7500
5000
2500
0
Slope
200
100
0
−100
cox1
pcox1−355
pcox1−241
pcox1−88
3'cox1
250000
252000
254000
Position

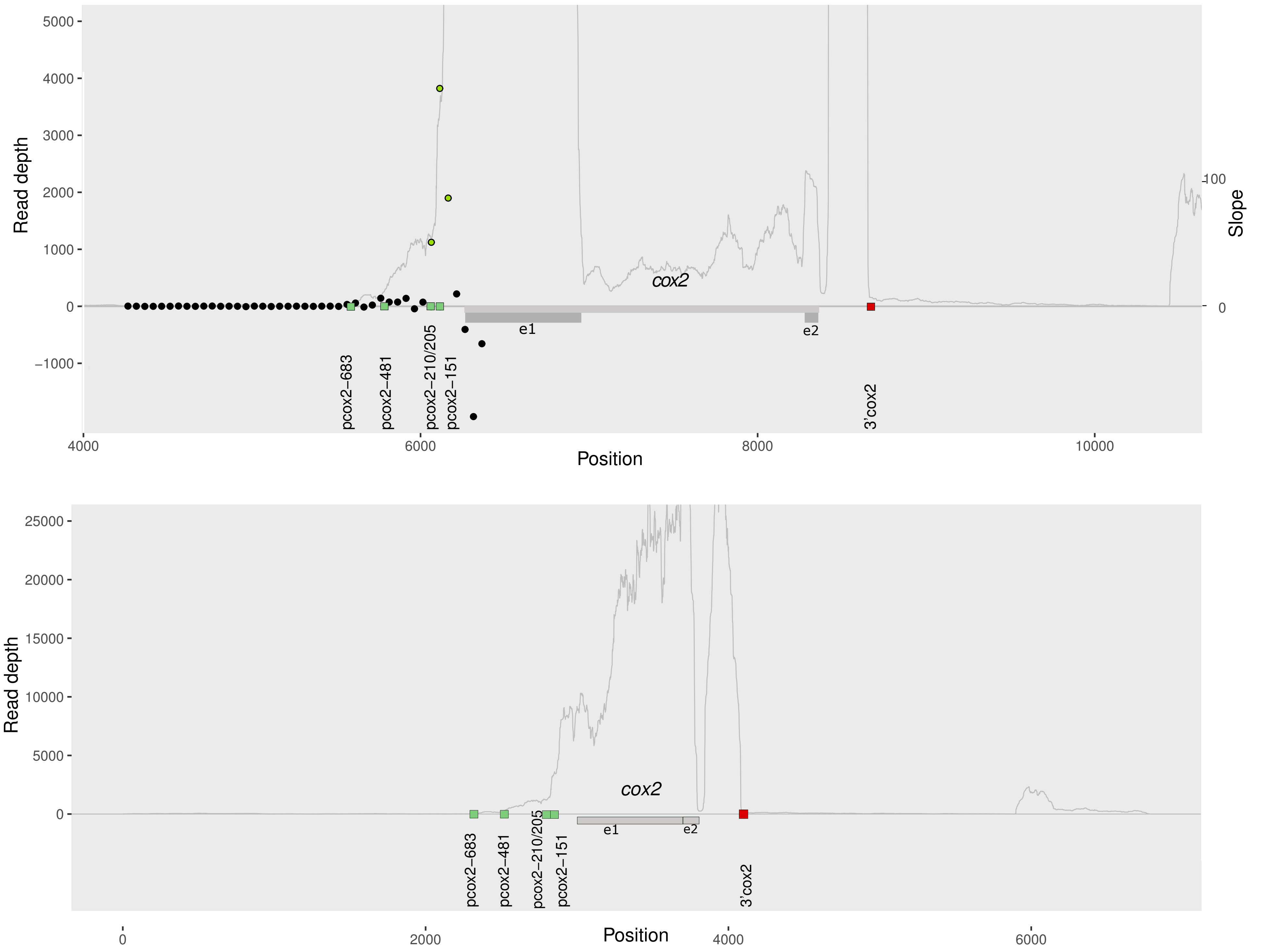
Read depth
Slope
Position
cox2
e1
e2
pcox2−683
pcox2−481
pcox2−210/205
pcox2−151
3'cox2
5000
4000
3000
2000
1000
0
−1000
100
0
4000
6000
8000
10000
Read depth
Position
cox2
e1
e2
pcox2−683
pcox2−481
pcox2−210/205
pcox2−151
3'cox2
25000
20000
15000
10000
5000
0
0
2000
4000
6000

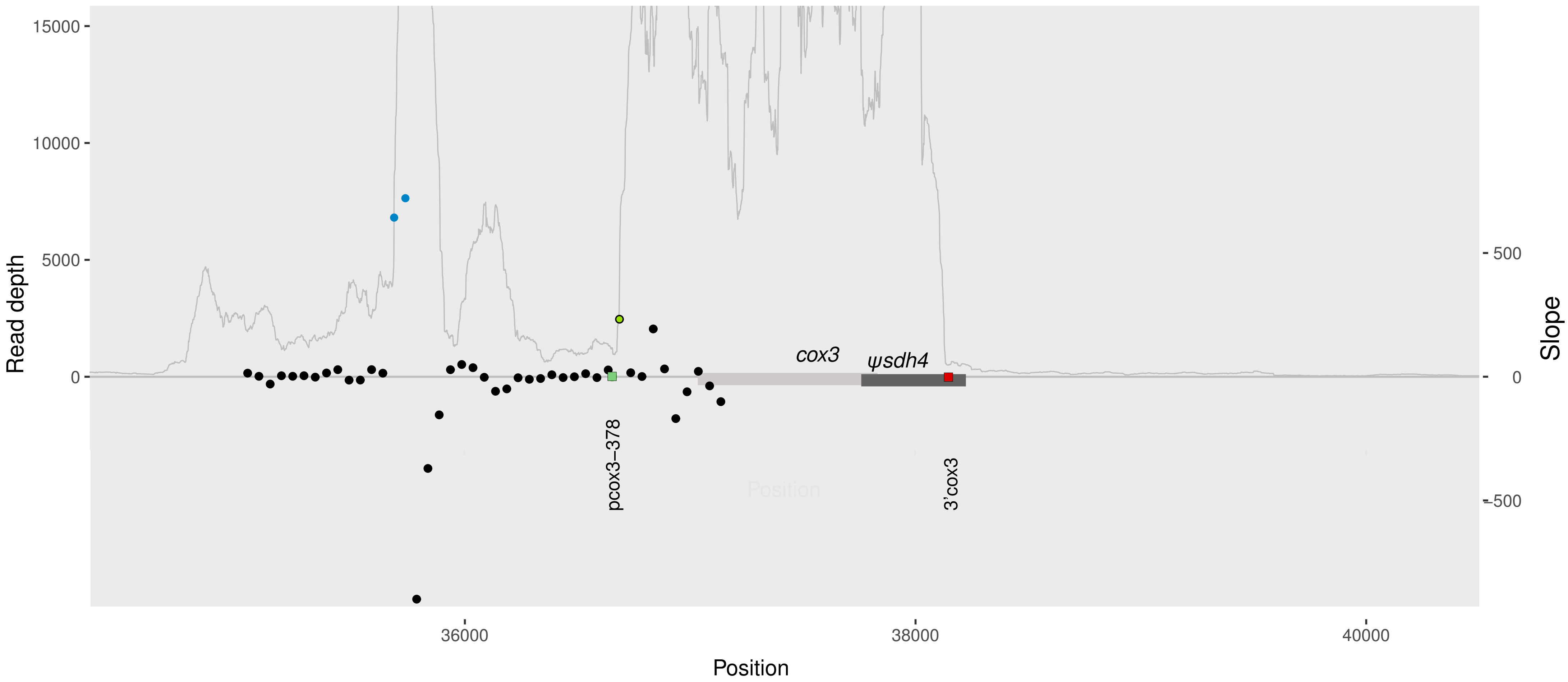

15000
10000
5000
0
Read depth
500
0
-500
Slope
cox3
ψsdh4
pcox3–378
3'cox3
36000
38000
40000
Position

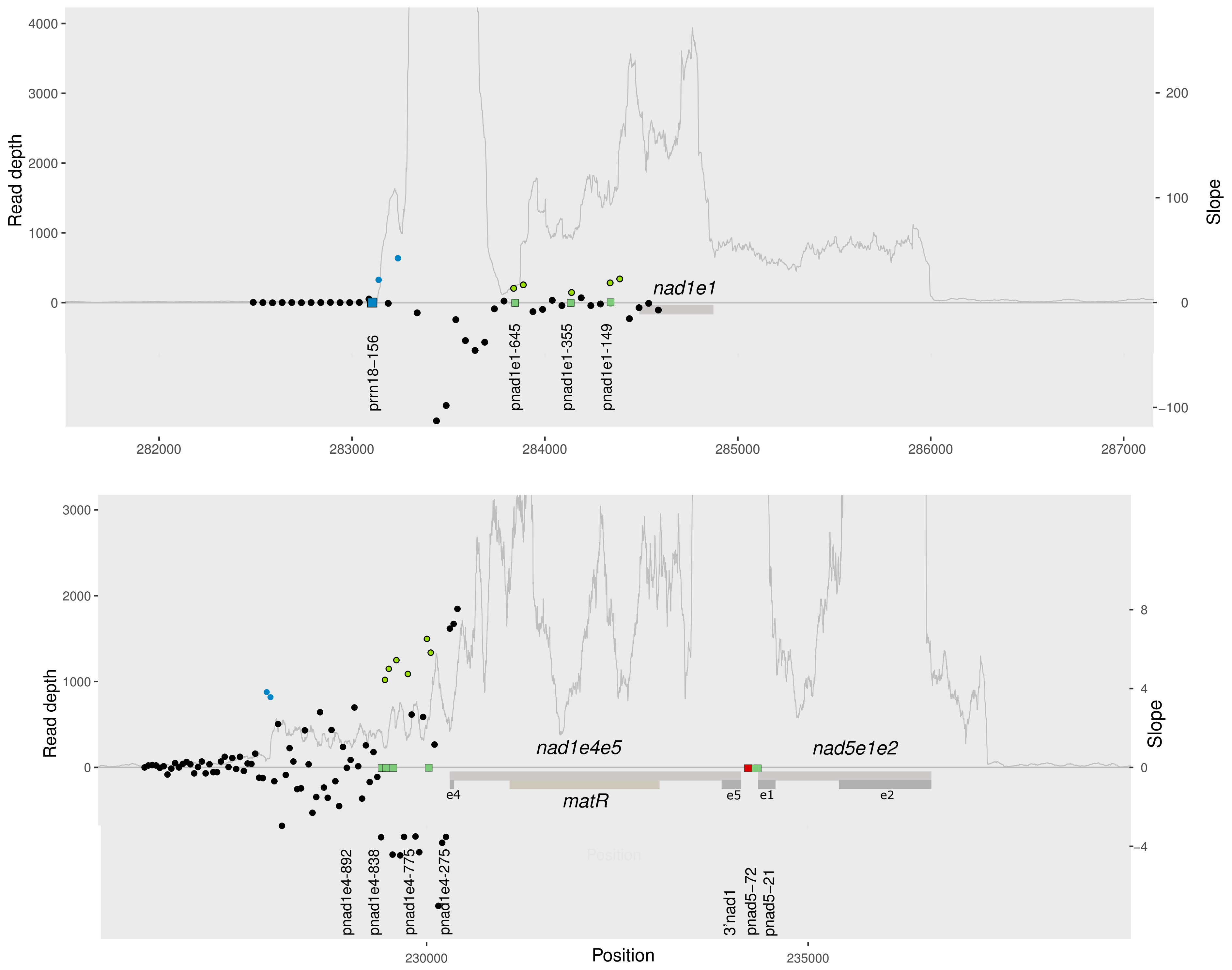

Read depth
Slope
nad1e1
prrn18−156
pnad1e1-645
pnad1e1-355
pnad1e1-149
282000
283000
284000
285000
286000
287000
nad1e4e5
nad5e1e2
matR
e4
e5
e1
e2
pnad1e4-892
pnad1e4-838
pnad1e4-775
pnad1e4-275
3'nad1
pnad5−72
pnad5−21
Position
230000
235000

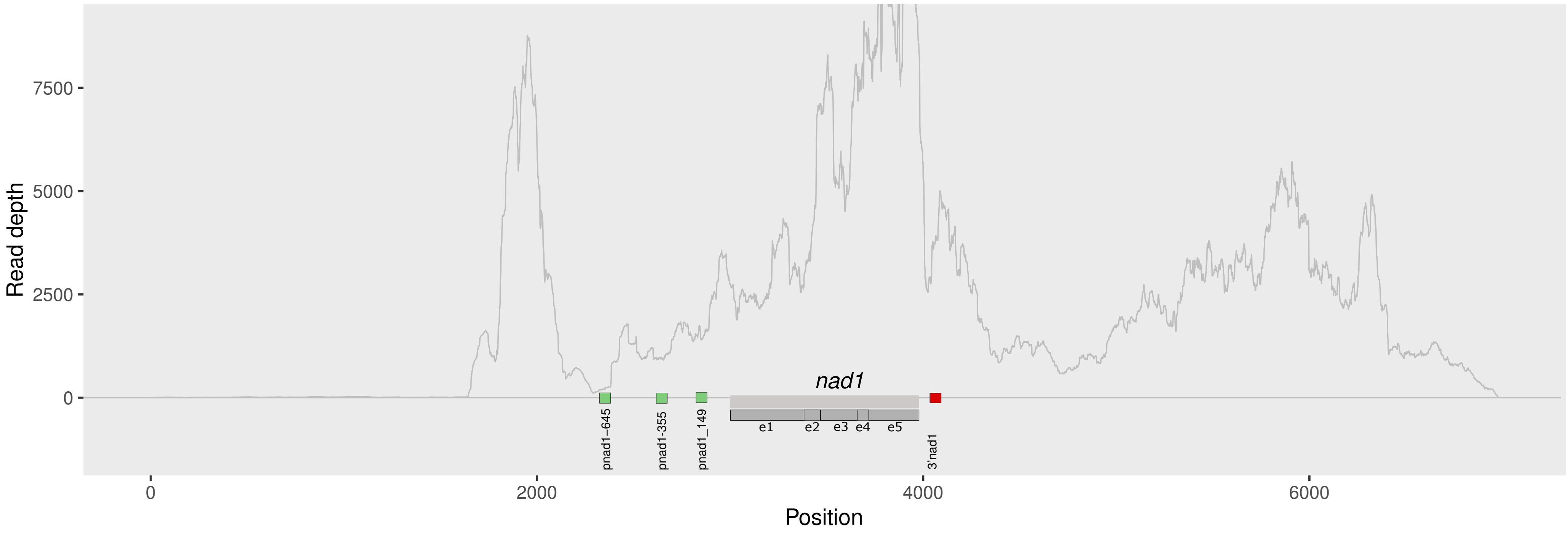

Read depth
7500
5000
2500
0
nad1
e1
e2
e3
e4
e5
pnad1-645
pnad1-355
pnad1_149
3'nad1
0
2000
4000
6000
Position

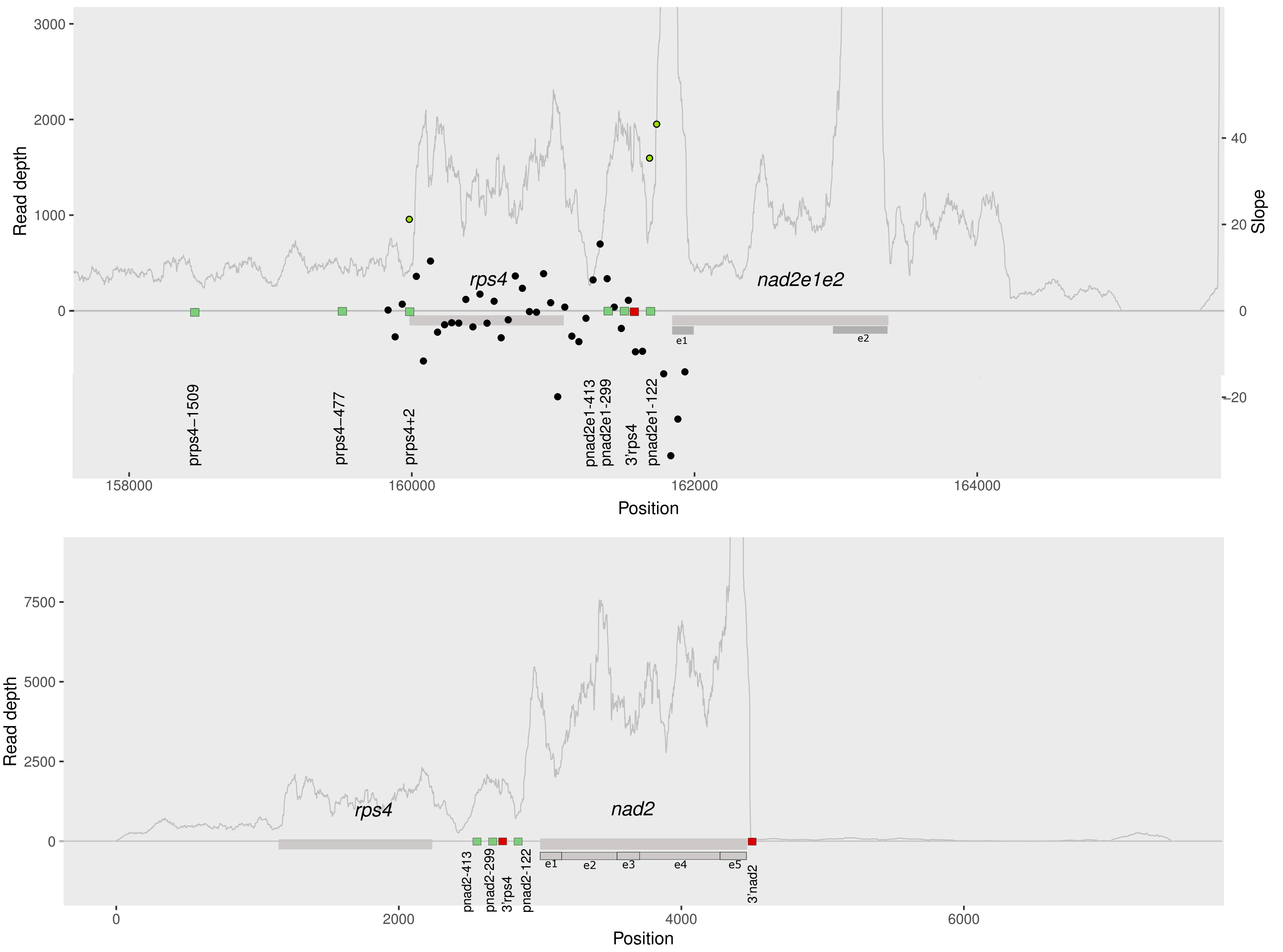
Read depth
Slope
rps4
nad2e1e2
e1
e2
prps4−1509
prps4−477
prps4+2
pnad2e1-413
pnad2e1-299
3'rps4
pnad2e1-122
Position
Read depth
rps4
nad2
e1
e2
e3
e4
e5
pnad2-413
pnad2-299
3'rps4
pnad2-122
3'nad2
Position

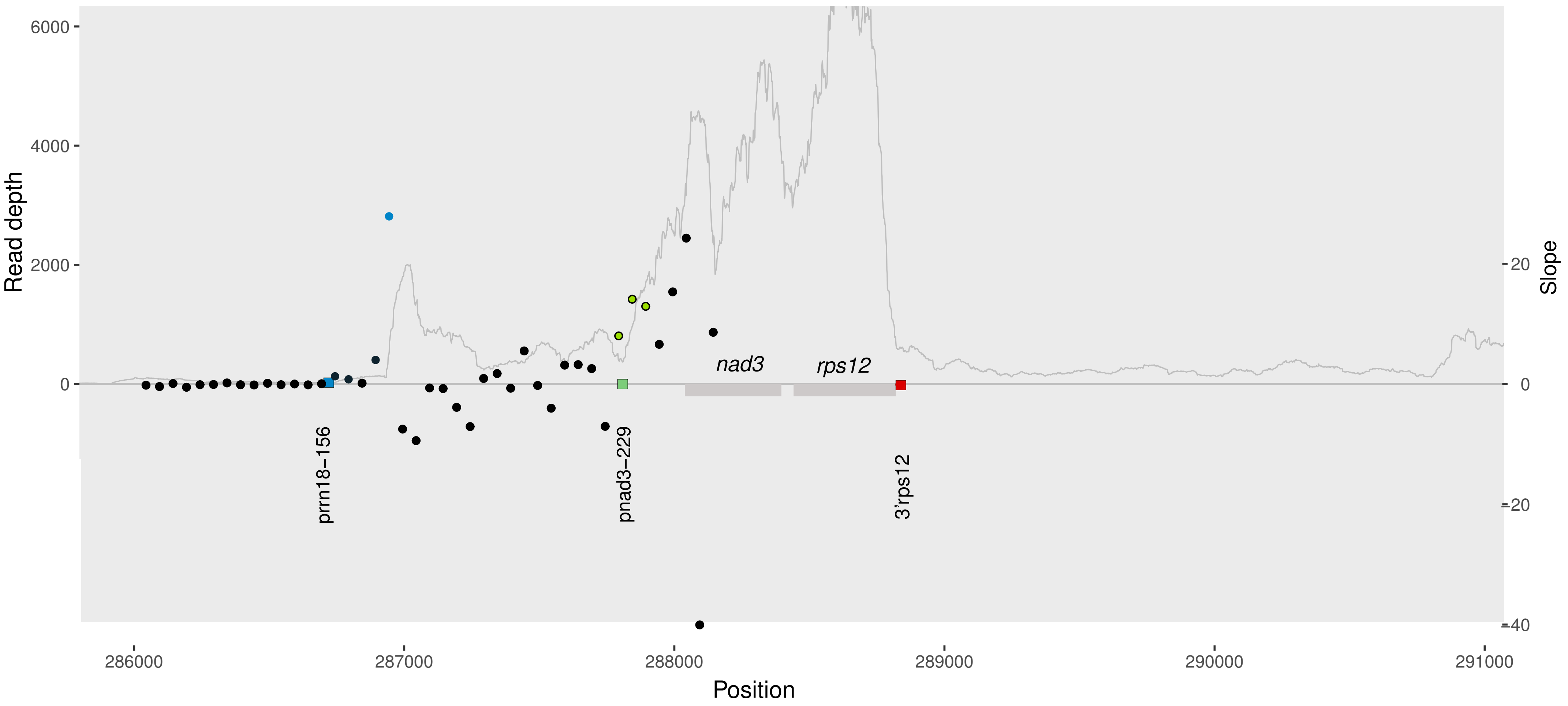

Read depth
6000
4000
2000
0
Slope
20
0
-20
-40
nad3
rps12
prrn18–156
pnad3–229
3'rps12
286000
287000
288000
289000
290000
291000
Position

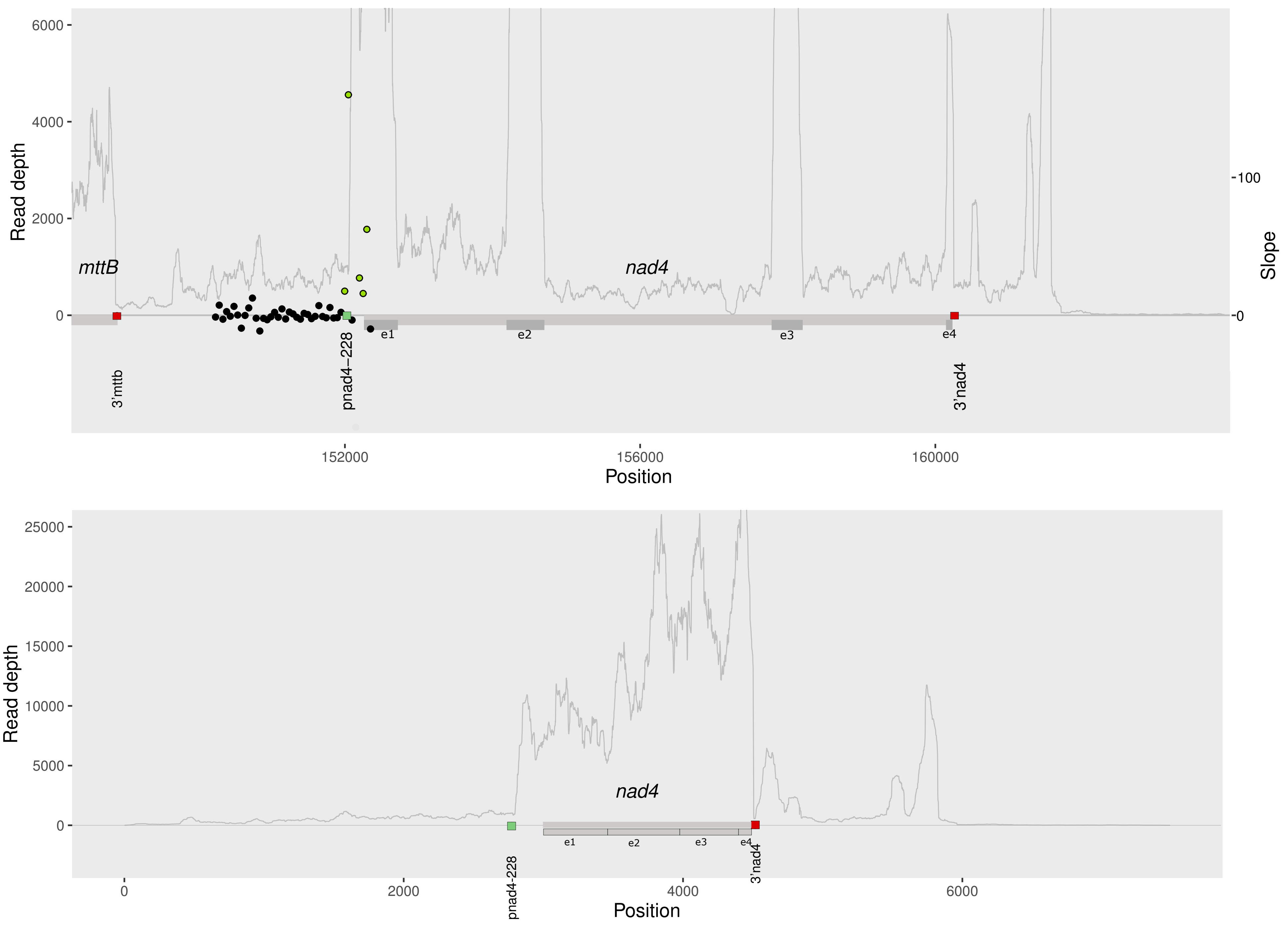

Read depth
Slope
mttB
nad4
3'mttb
pnad4−228
3'nad4
e1
e2
e3
e4
6000
4000
2000
0
100
152000
156000
160000
Position
Read depth
nad4
pnad4-228
3'nad4
25000
20000
15000
10000
5000
0
2000
4000
6000
Position

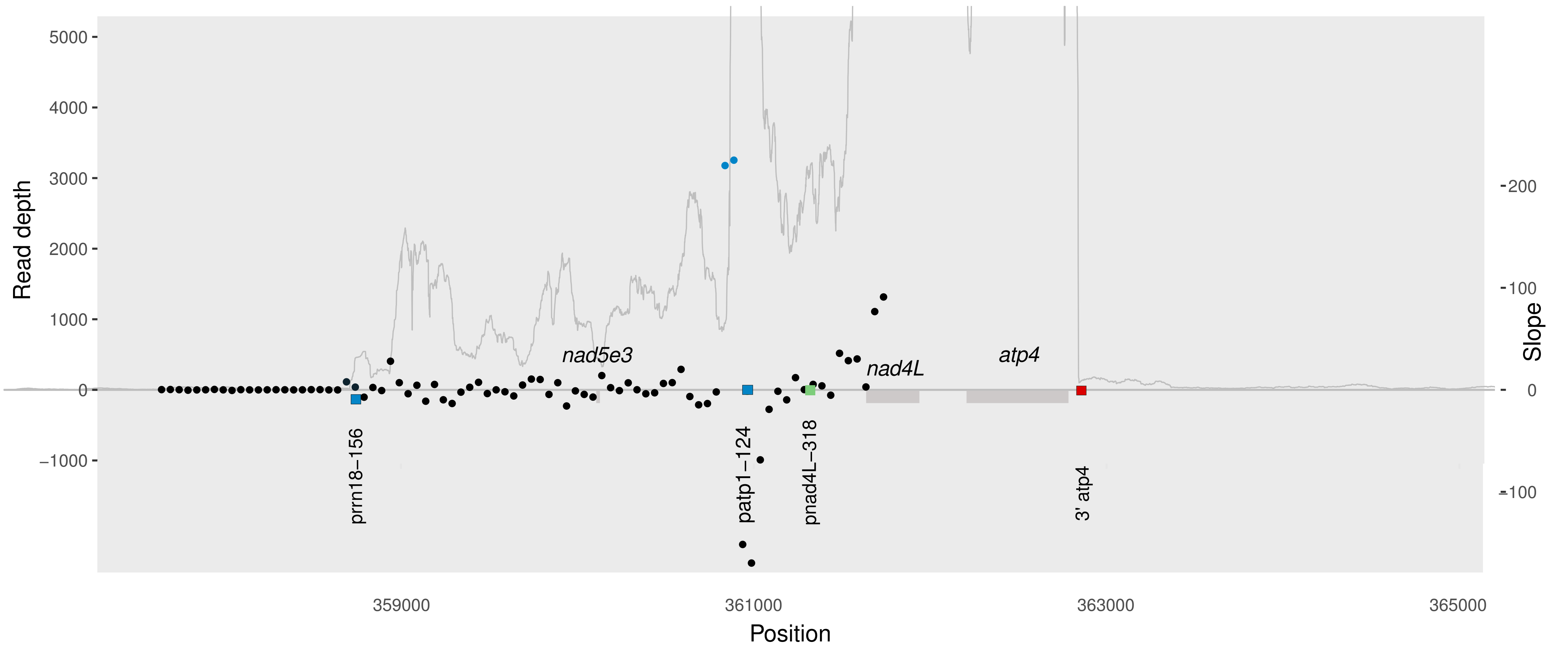

Read depth
5000
4000
3000
2000
1000
0
−1000
Slope
200
100
0
−100
nad5e3
nad4L
atp4
prrn18−156
patp1−124
pnad4L−318
3' atp4
359000
361000
363000
365000
Position

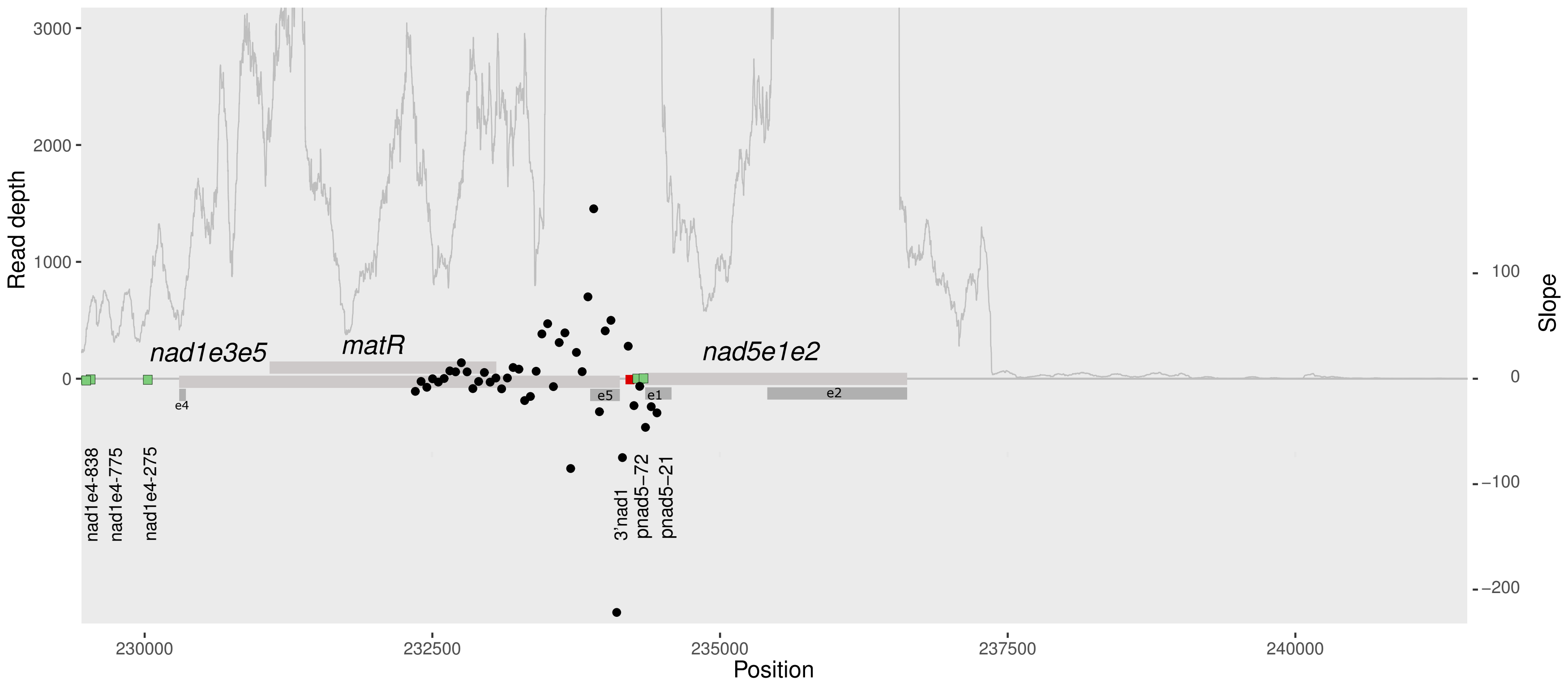

Read depth
Slope
nad1e3e5
matR
nad5e1e2
e4
e5
e1
e2
nad1e4-838
nad1e4-775
nad1e4-275
3'nad1
pnad5−72
pnad5−21
Position

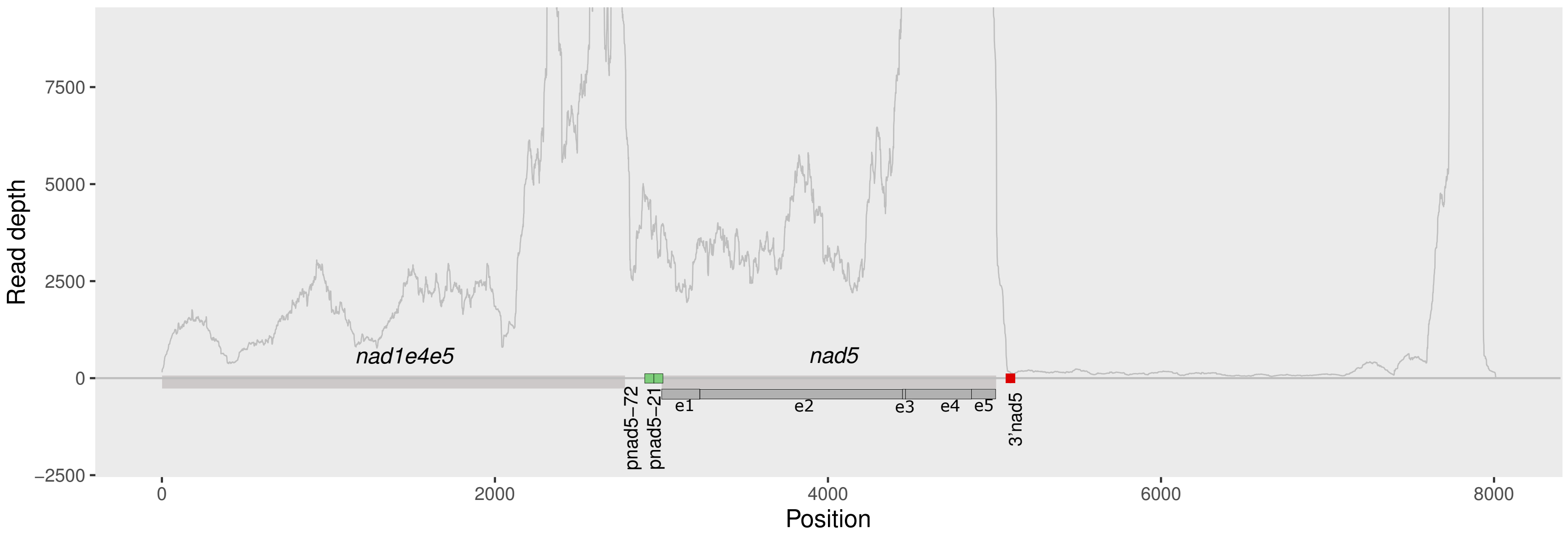

Read depth
nad1e4e5
nad5
e1
e2
e3
e4
e5
pnad5−72
pnad5−21
3'nad5
Position

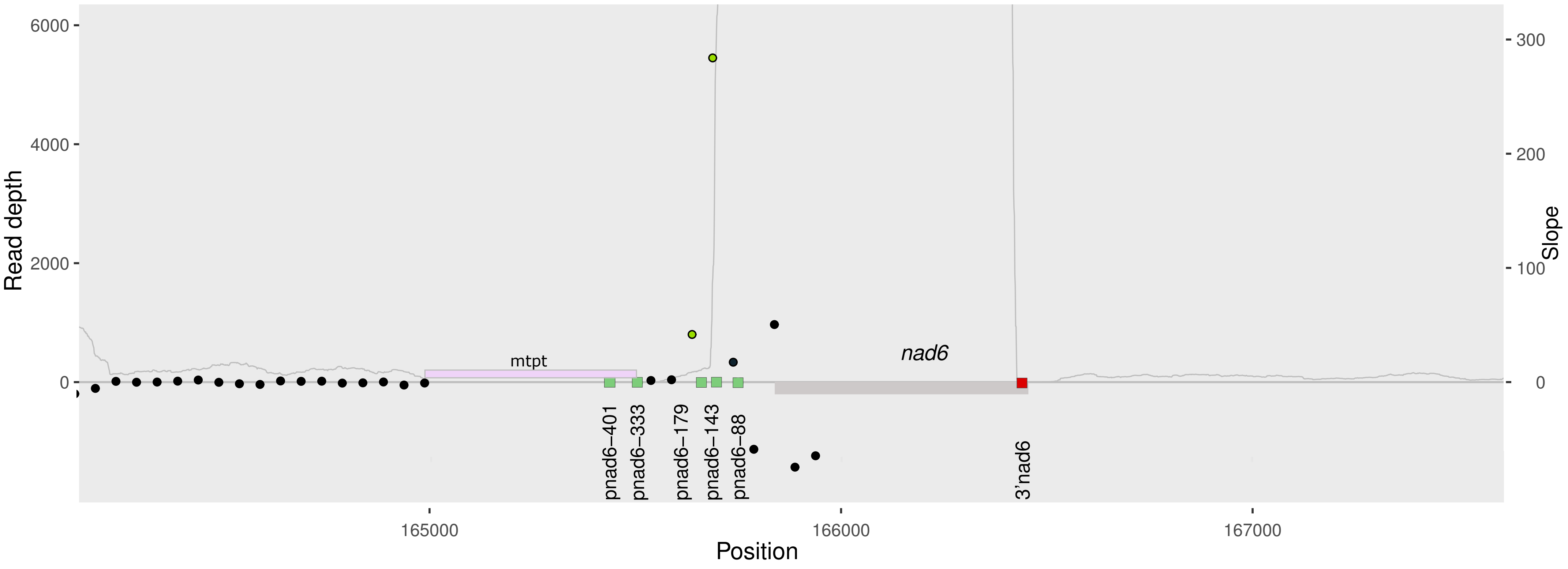

Read depth
6000
4000
2000
0
Slope
300
200
100
0
mtpt
nad6
pnad6−401
pnad6−333
pnad6−179
pnad6−143
pnad6−88
3'nad6
165000
166000
167000
Position

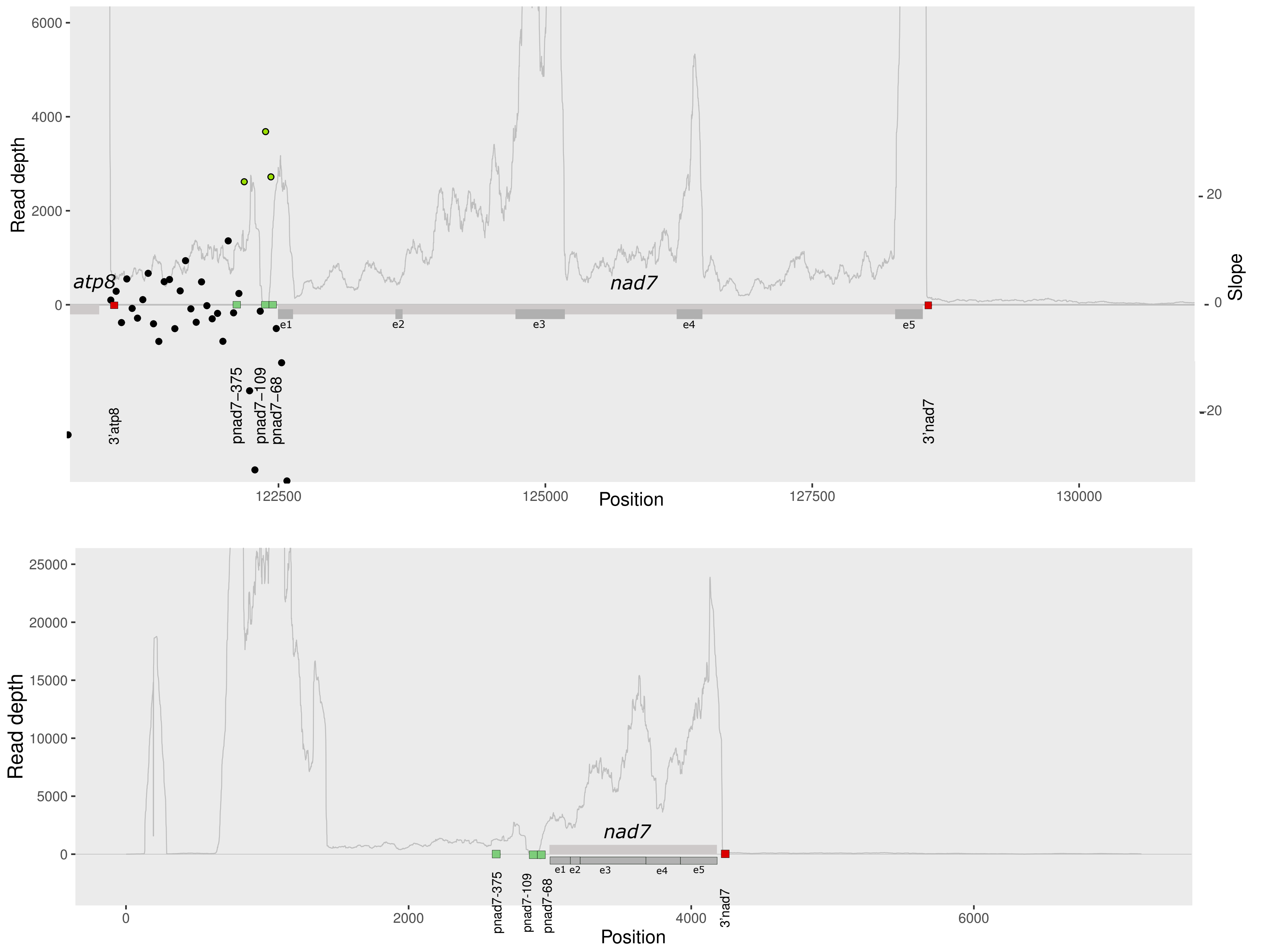

Read depth
6000
4000
2000
0
atp8
nad7
e1
e2
e3
e4
e5
3'atp8
pnad7−375
pnad7−109
pnad7−68
3'nad7
Slope
20
0
-20
122500
125000
127500
130000
Position
Read depth
25000
20000
15000
10000
5000
0
nad7
e1
e2
e3
e4
e5
pnad7-375
pnad7-109
pnad7-68
3'nad7
0
2000
4000
6000
Position

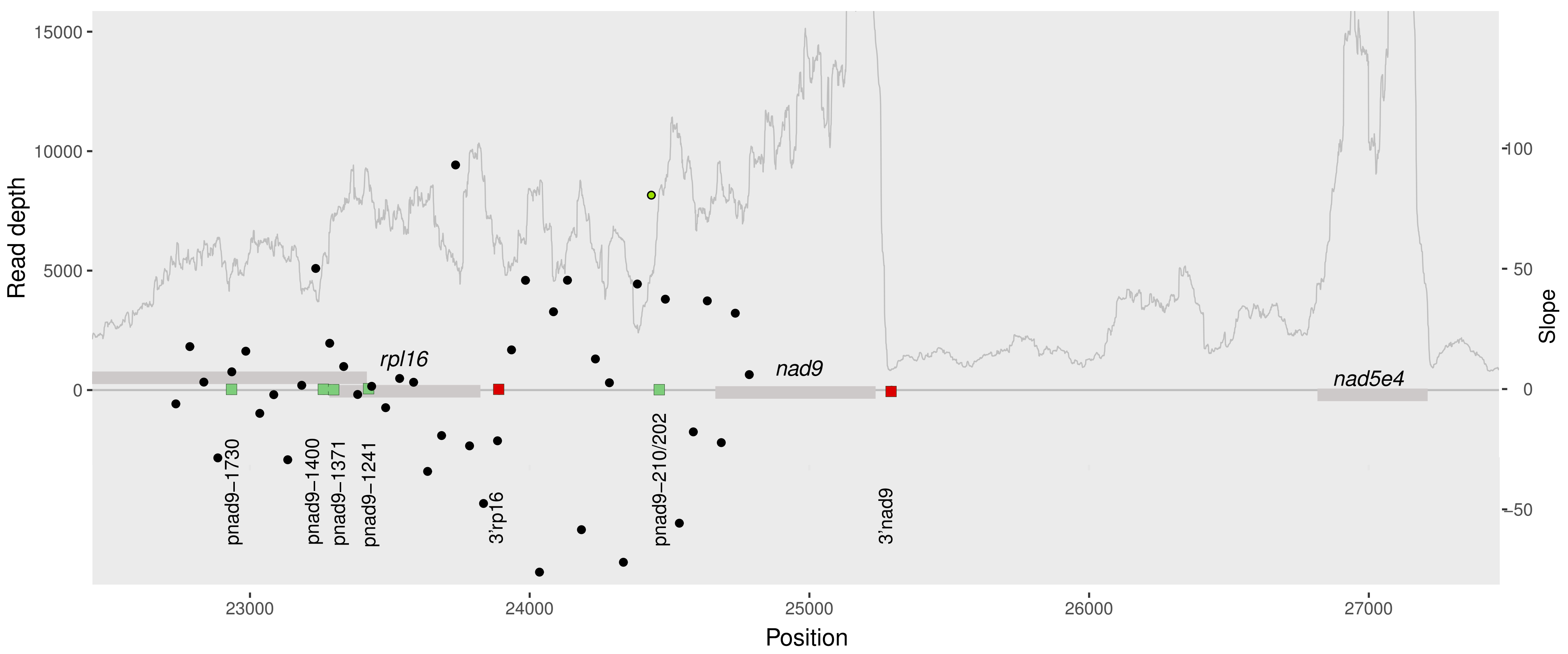

Read depth
15000
10000
5000
0
Slope
100
50
0
−50
rpl16
nad9
nad5e4
pnad9−1730
pnad9−1400
pnad9−1371
pnad9−1241
3'rp16
pnad9−210/202
3'nad9
23000
24000
25000
26000
27000
Position

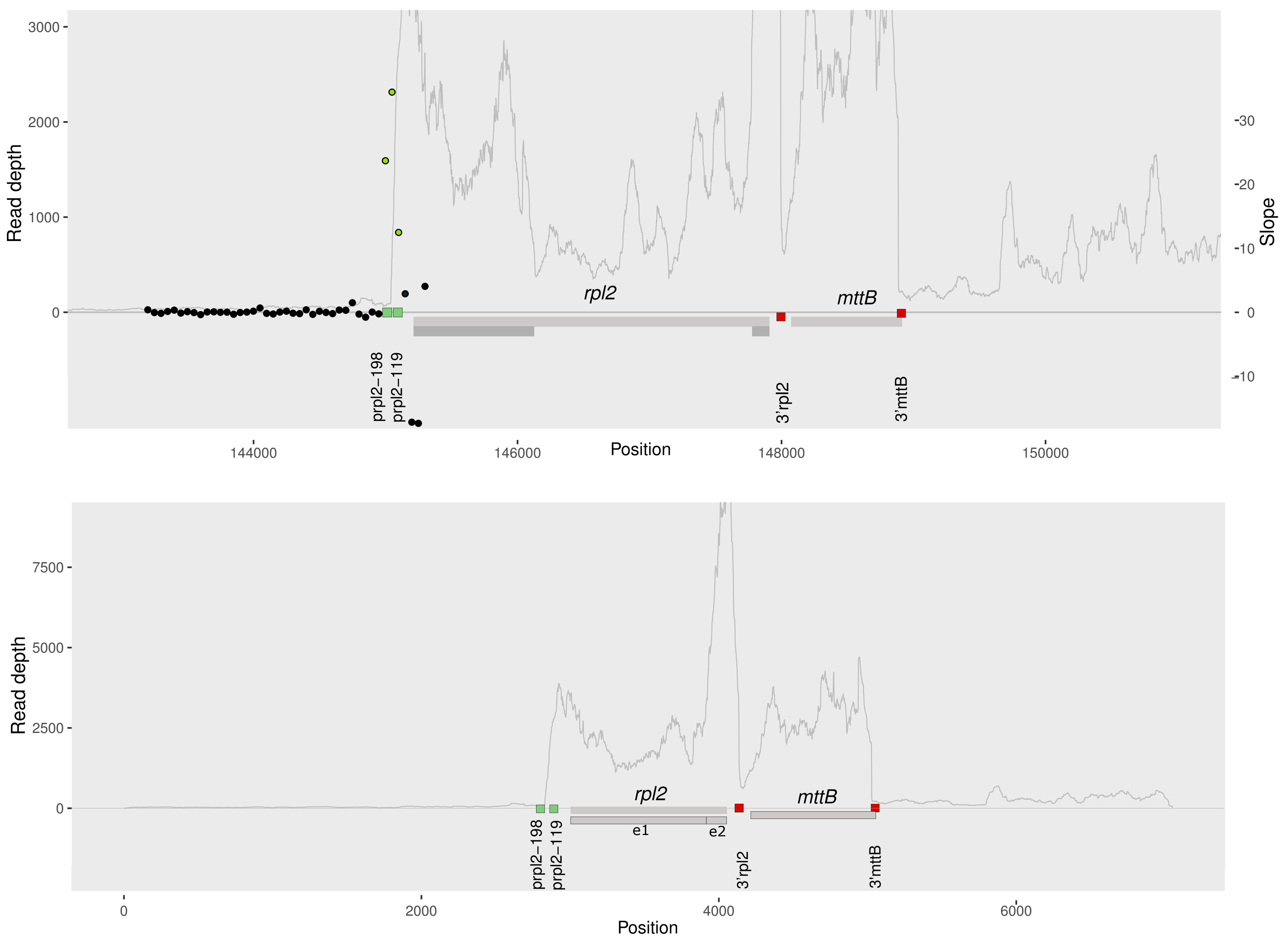

Read depth
Slope
Position
rpl2
mttB
prpl2−198
prpl2−119
3'rpl2
3'mttB
e1
e2

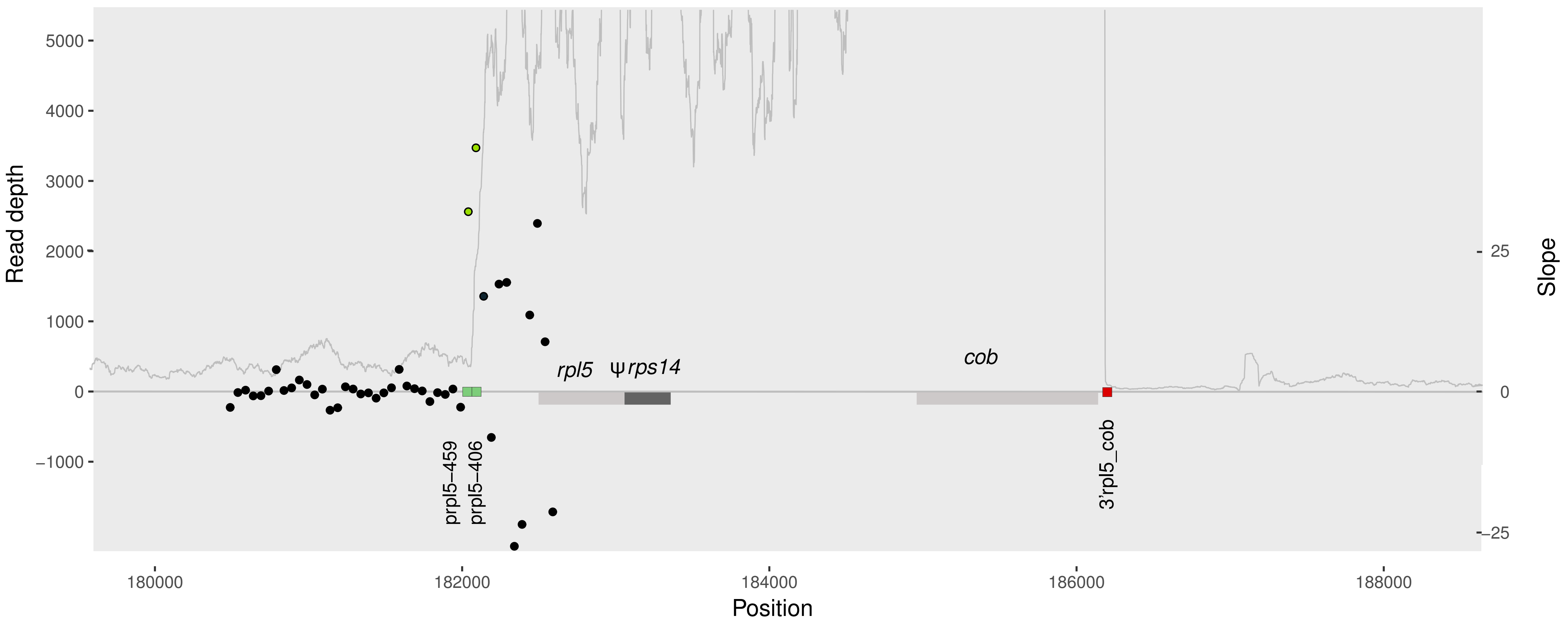

Read depth
Slope
5000
4000
3000
2000
1000
0
−1000
25
0
−25
rpl5
Ψ rps14
cob
prpl5−459
prpl5−406
3'rpl5_cob
180000
182000
184000
186000
188000
Position

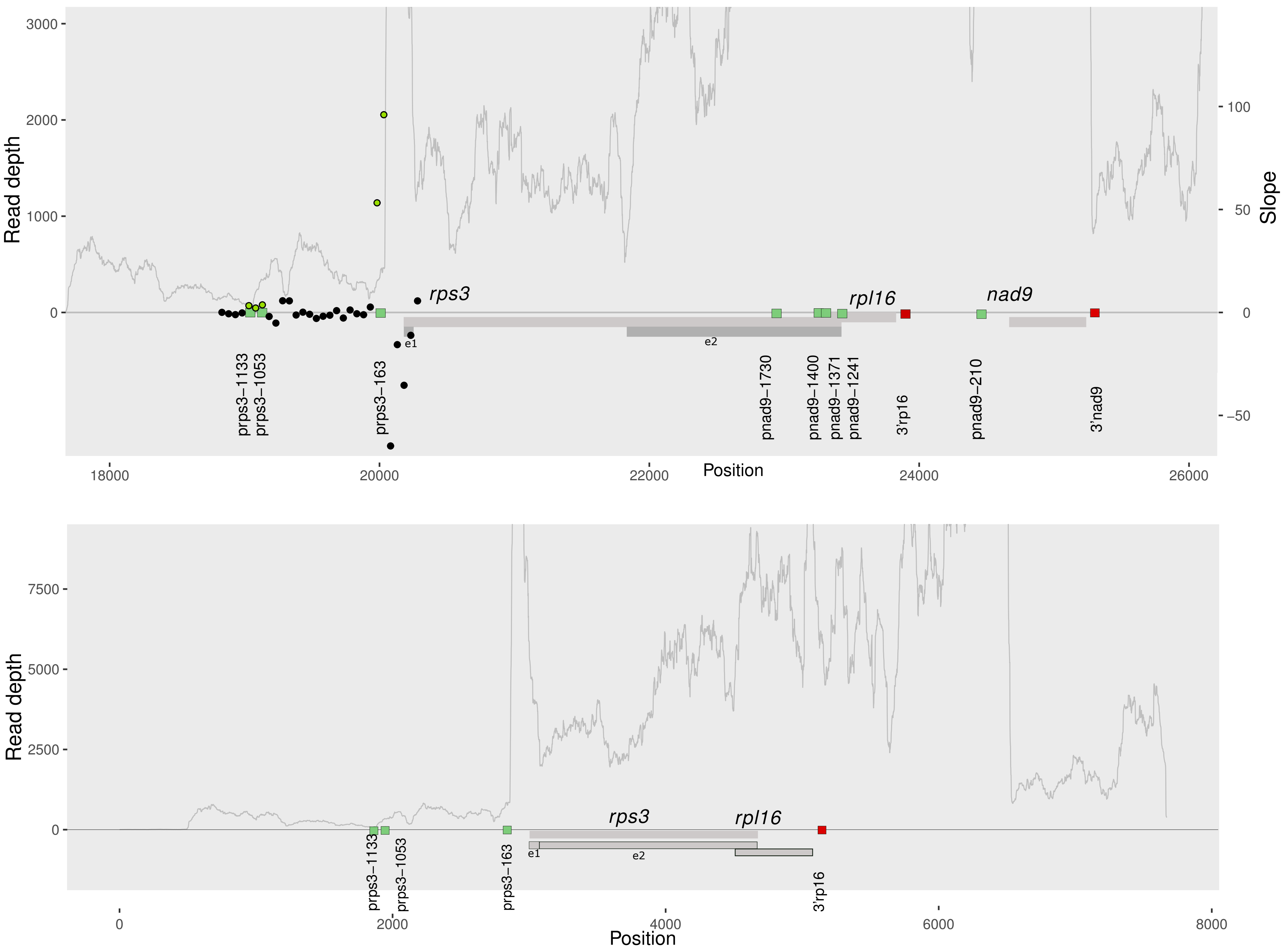

Read depth
Slope
Position
rps3
rpl16
nad9
e1
e2
prps3−1133
prps3−1053
prps3−163
pnad9−1730
pnad9−1400
pnad9−1371
pnad9−1241
3'rp16
pnad9−210
3'nad9
3000
2000
1000
0
100
50
0
−50
18000
20000
22000
24000
26000
7500
5000
2500
0
2000
4000
6000
8000

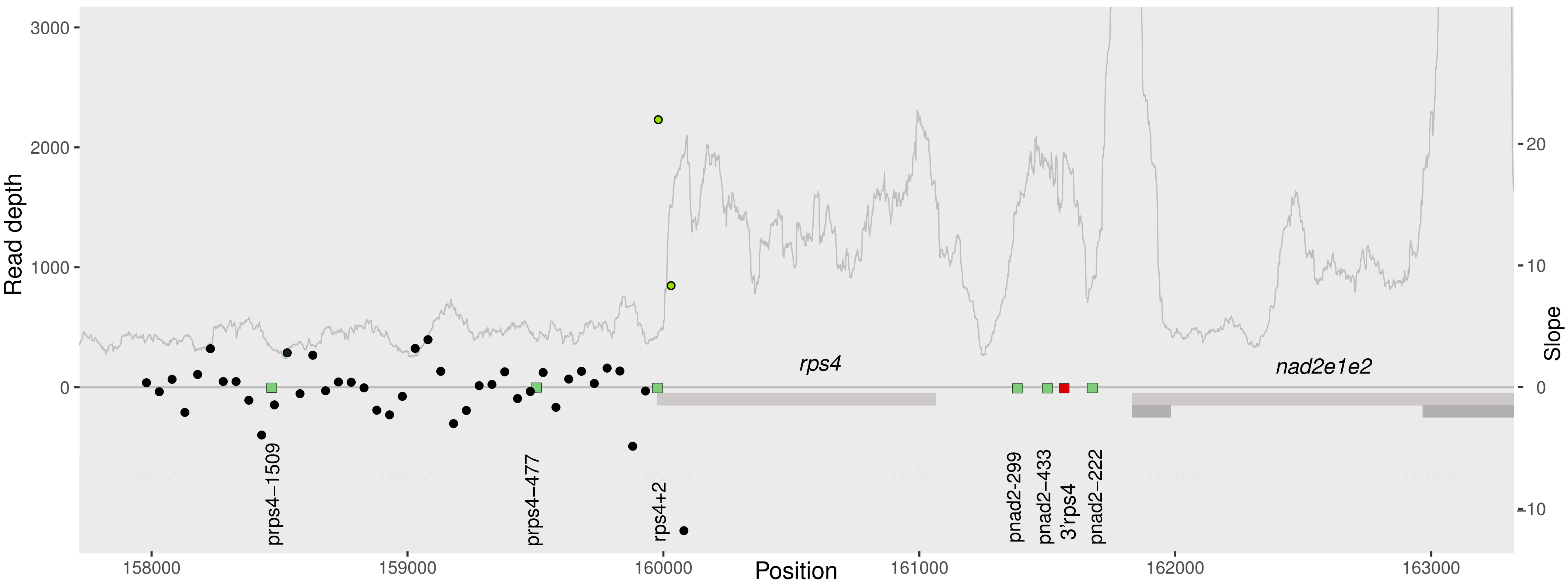
Read depth
3000
2000
1000
0
Slope
20
10
0
-10
rps4
nad2e1e2
prps4−1509
prps4−477
rps4+2
pnad2-299
pnad2−433
3'rps4
pnad2−222
158000
159000
160000
161000
162000
163000
Position

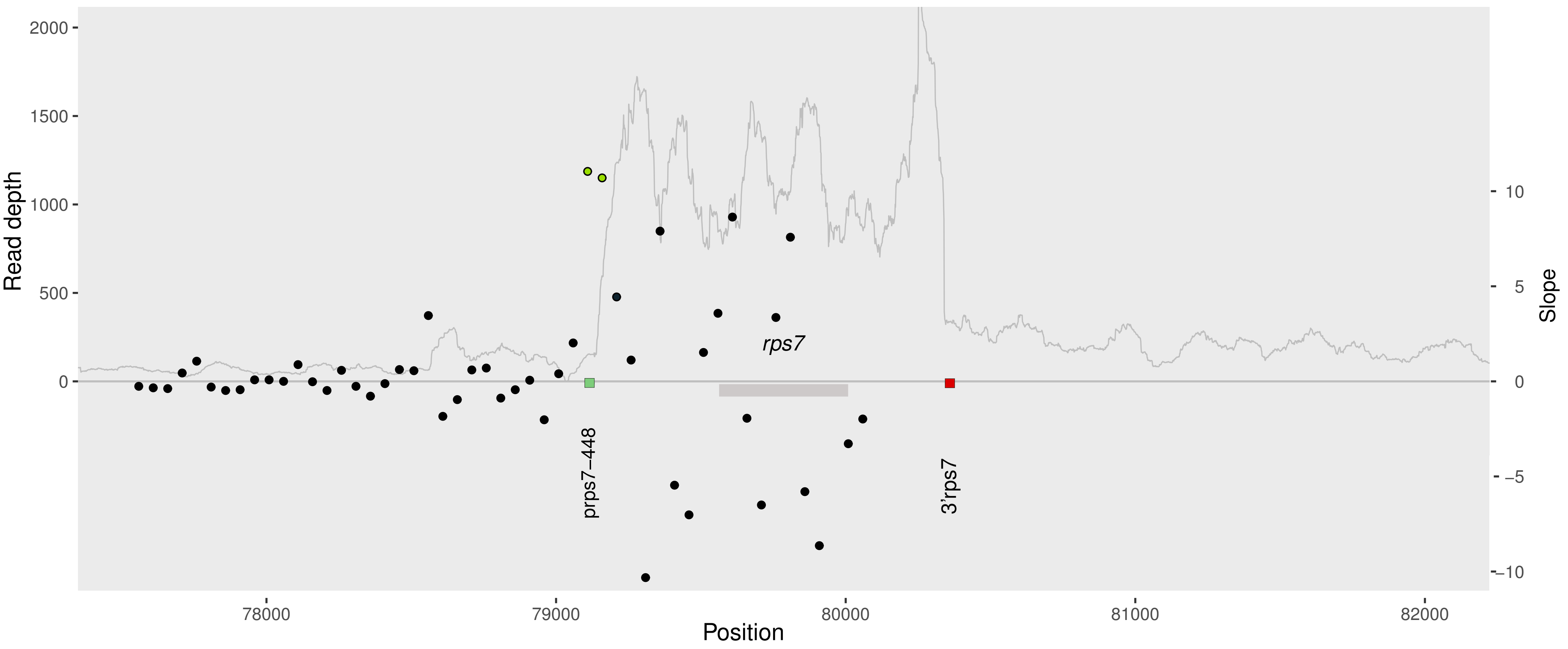
Read depth
Slope
Position
2000
1500
1000
500
0
10
5
0
–5
–10
78000
79000
80000
81000
82000
rps7
prps7–448
3'rps7
