# Supplementary material for: Transcriptional Landscape and Splicing Efficiency in Arabidopsis Mitochondria
Source: Cells. 2021 Aug 11;10(8):2054. doi: 10.3390/cells10082054 (PMC8392254; doi:10.3390/cells10082054)
Supplement: Supplementary file 1 [file cells-10-02054-s001.zip › FigS5.pdf]

Fig. S5. RNA read depth of protein-coding genes located at a distance < 2 kb in the *Arabidopsis* mtDNA based on the combined data sets of the three biological samples. Gray bars represent genes and dark gray bars are exons. Green and red squares represent the start of transcription sites and 3' termini, respectively, which were identified experimentally [36,42,44]. The start of transcription for nad5e4e5 has not been experimentally identified. Below each graph, the mtDNA coordinates of the strand in which the genes are located are shown.

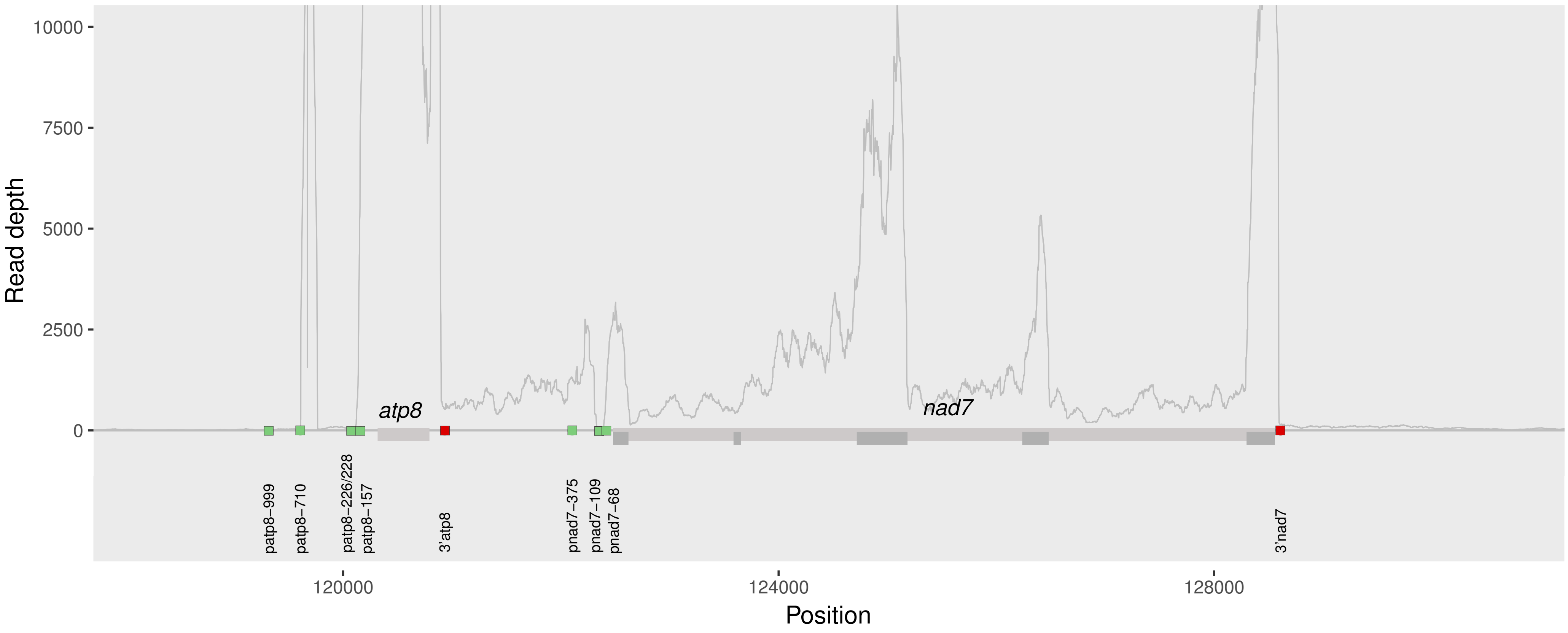

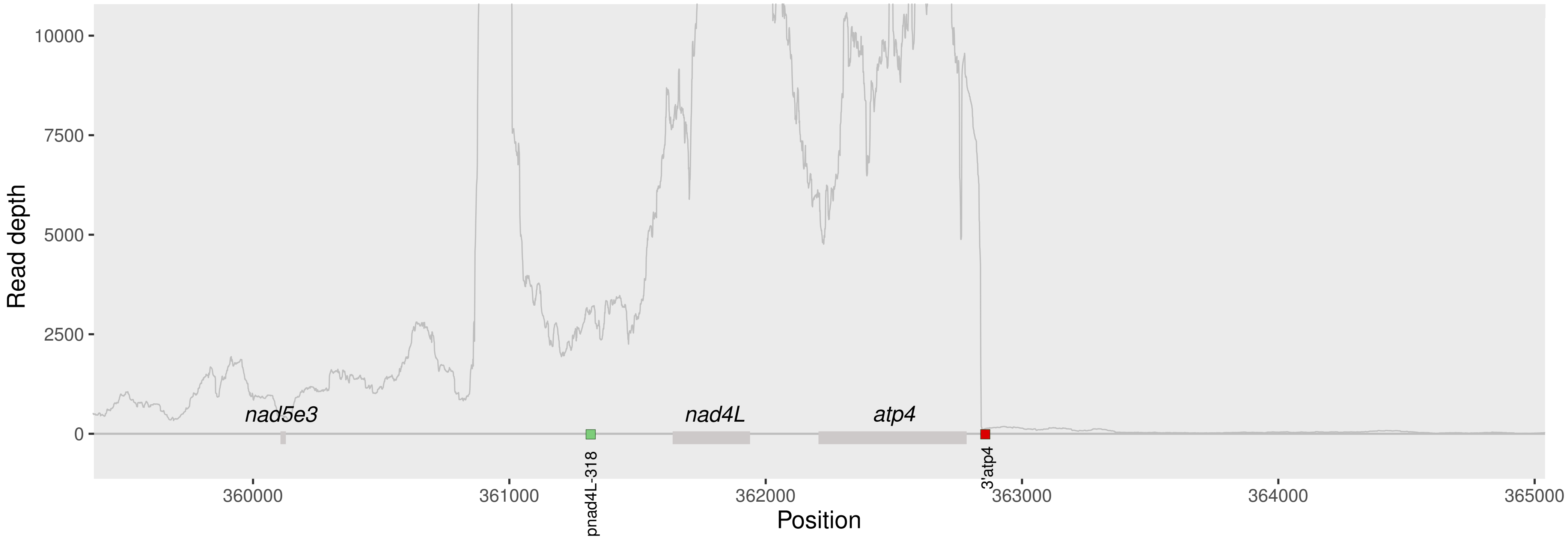

Read depth
10000
7500
5000
2500
0
nad5e3
nad4L
atp4
pnad4L-318
3'atp4
360000
361000
362000
363000
364000
365000
Position

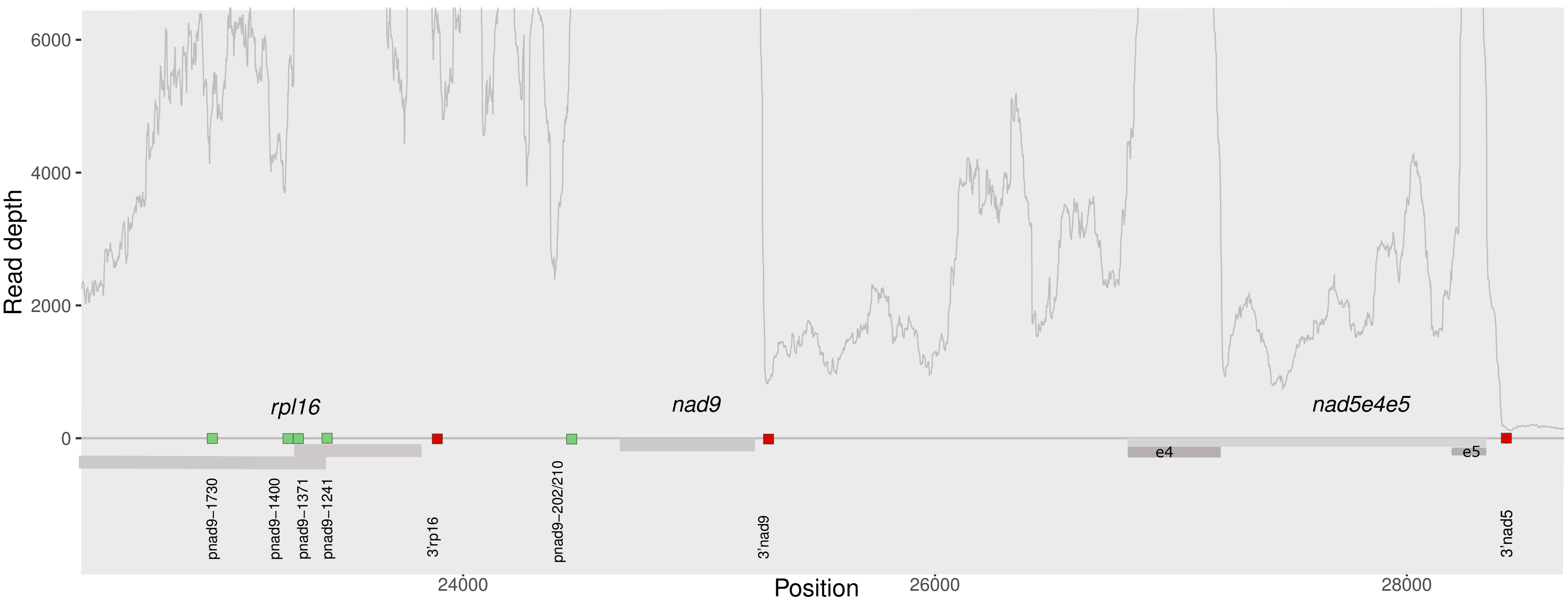

Read depth
6000
4000
2000
0
rpl16
nad9
nad5e4e5
e4
e5
pnad9−1730
pnad9−1400
pnad9−1371
pnad9−1241
3'rp16
pnad9−202/210
3'nad9
3'nad5
24000
26000
28000
Position

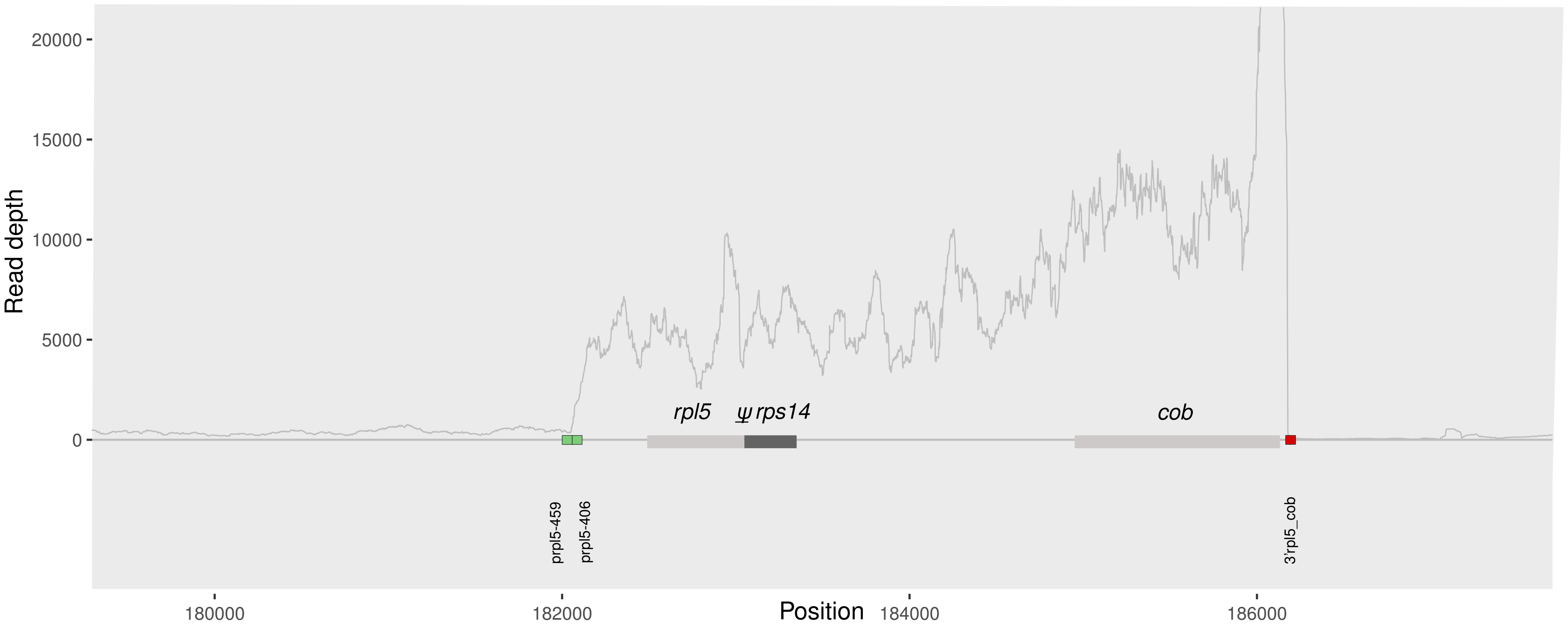

Read depth
20000
15000
10000
5000
0
rpl5
ψ rps14
cob
prpl5-459
prpl5-406
3'rpl5_cob
180000
182000
184000
186000
Position

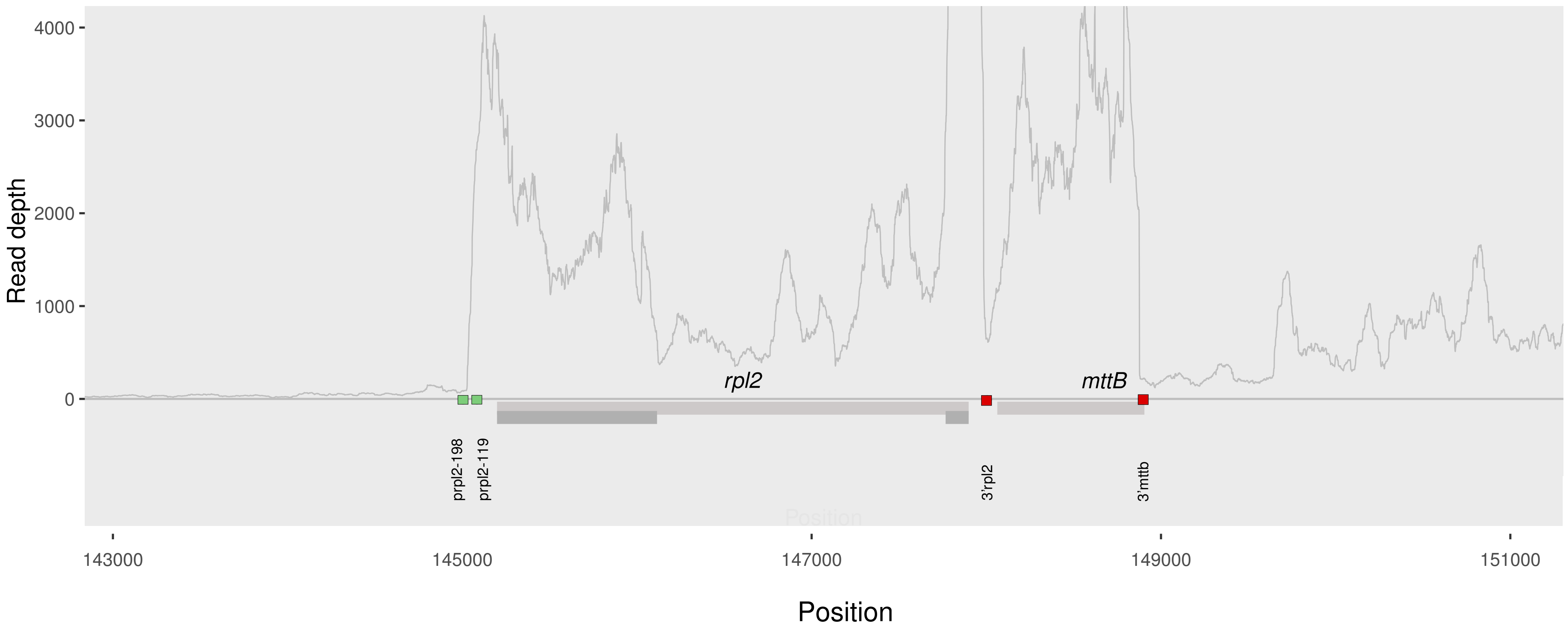

Read depth
4000
3000
2000
1000
0
rpl2
mttB
prpl2-198
prpl2-119
3'rpl2
3'mttb
143000
145000
147000
149000
151000
Position

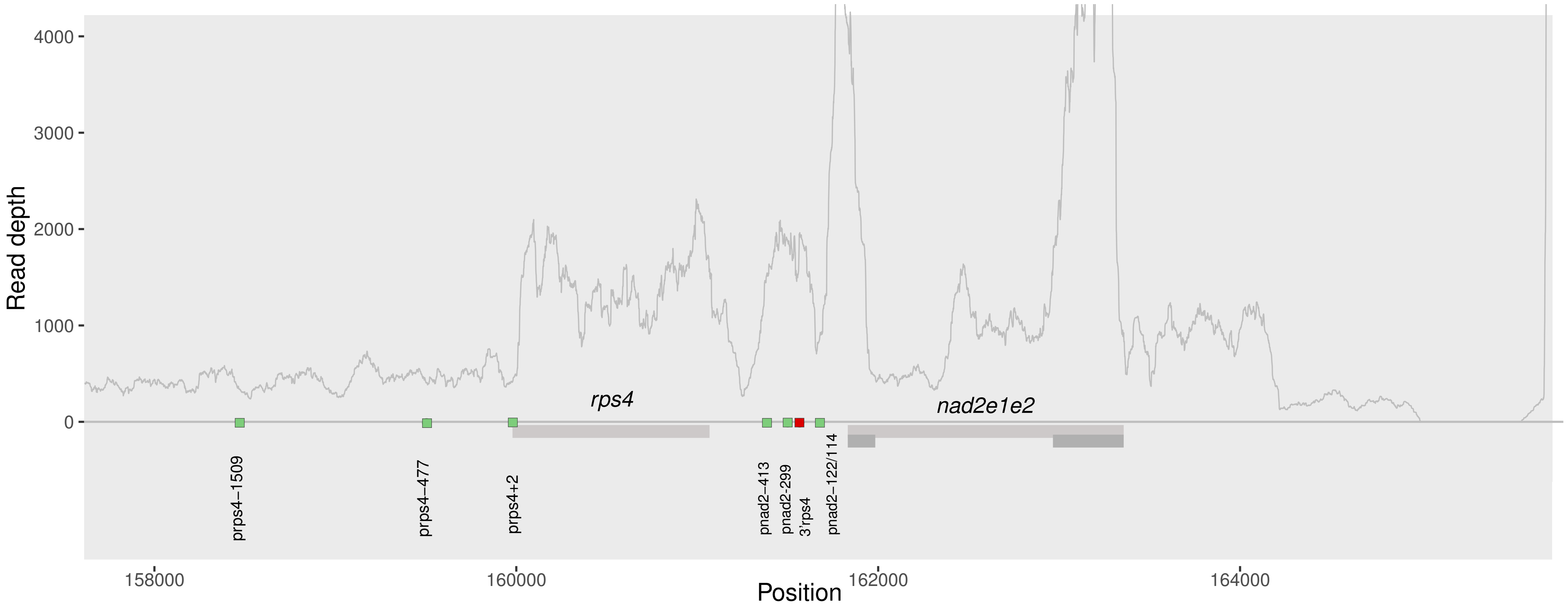

Read depth
4000
3000
2000
1000
0
rps4
nad2e1e2
prps4−1509
prps4−477
prps4+2
pnad2−413
pnad2−299
3'rps4
pnad2−122/114
158000
160000
162000
164000
Position
